# Genome-wide CRISPR screening identifies tyrosylprotein sulfotransferase-2 as a target for augmenting anti-PD1 efficacy

**DOI:** 10.1186/s12943-024-02068-x

**Published:** 2024-08-02

**Authors:** Yumi Oh, Sujeong Kim, Yunjae Kim, Hyun Kim, Dongjun Jang, Seungjae Shin, Soo-Jin Lee, Jiwon Kim, Sang Eun Lee, Jaeik Oh, Yoojin Yang, Dohee Kim, Hae Rim Jung, Sangjin Kim, Jihui Kim, Kyungchan Min, Beomki Cho, Hoseok Seo, Dohyun Han, Hansoo Park, Sung-Yup Cho

**Affiliations:** 1 Medical Research Center, Genomic Medicine Institute, Seoul National University College of Medicine, Seoul, 03080 Korea; 2 Department of Biomedical Science and Engineering, Gwangju Institute of Science and Technology (GIST), Gwangju, 61005 Korea; 3 Department of Biomedical Sciences, Seoul National University College of Medicine, Seoul, 03080 Korea; 4 Department of Translational Medicine, Seoul National University College of Medicine, Seoul, 03080 Korea; 5 Department of Transdisciplinary Medicine, Seoul National University Hospital, Seoul, 03080 Korea; 6 Interdisciplinary Program in Neuroscience, College of Natural Sciences, Seoul National University, Seoul, 08826 Korea; 7 Department of Medicine, Seoul National University College of Medicine, Seoul, 03080 Korea; 8 Genome&Company, Suwon, 16229 Korea; 9 Cancer Research Institute, Seoul National University, Seoul, 03080 Korea

**Keywords:** Immune checkpoint therapy, CRISPR screening, Tyrosylprotein sulfotransferase-2, Interferon-γ, Antigen presentation

## Abstract

**Background:**

Immune checkpoint therapy (ICT) provides durable responses in select cancer patients, yet resistance remains a significant challenge, prompting the exploration of underlying molecular mechanisms. Tyrosylprotein sulfotransferase-2 (TPST2), known for its role in protein tyrosine O-sulfation, has been suggested to modulate the extracellular protein-protein interactions, but its specific role in cancer immunity remains largely unexplored.

**Methods:**

To explore tumor cell-intrinsic factors influencing anti-PD1 responsiveness, we conducted a pooled loss-of-function genetic screen in humanized mice engrafted with human immune cells. The responsiveness of cancer cells to interferon-γ (IFNγ) was estimated by evaluating IFNγ-mediated induction of target genes, STAT1 phosphorylation, HLA expression, and cell growth suppression. The sulfotyrosine-modified target gene of TPST2 was identified by co-immunoprecipitation and mass spectrometry. The in vivo effects of TPST2 inhibition were evaluated using mouse syngeneic tumor models and corroborated by bulk and single-cell RNA sequencing analyses.

**Results:**

Through in vivo genome-wide CRISPR screening, TPST2 loss-of-function emerged as a potential enhancer of anti-PD1 treatment efficacy. TPST2 suppressed IFNγ signaling by sulfating IFNγ receptor 1 at Y397 residue, while its downregulation boosted IFNγ-mediated signaling and antigen presentation. Depletion of TPST2 in cancer cells augmented anti-PD1 antibody efficacy in syngeneic mouse tumor models by enhancing tumor-infiltrating lymphocytes. RNA sequencing data revealed TPST2’s inverse correlation with antigen presentation, and increased TPST2 expression is associated with poor prognosis and altered cancer immunity across cancer types.

**Conclusions:**

We propose TPST2’s novel role as a suppressor of cancer immunity and advocate for its consideration as a therapeutic target in ICT-based treatments.

**Supplementary Information:**

The online version contains supplementary material available at 10.1186/s12943-024-02068-x.

## Introduction

Immune checkpoint therapy (ICT) including anti-PD1 antibody has demonstrated durable responses of tumors and cures for subset of cancer patients [1]. One of obstacles in ICT is the development of resistance due to tumor cell intrinsic and extrinsic factors, which is observed in a significant proportion of patients [2]. To overcome this resistance, numerous ICT-based combination approaches have been suggested, and many of them have been tested in clinical trials [3]. However, the exact underlying mechanisms that determine resistance to ICT remain poorly understood, and the development of rational combination strategies based on the molecular mechanisms of resistance is required [1].

Tyrosylprotein sulfotransferase-2 (TPST2) is a Golgi enzyme that catalyzes the posttranslational modifications of proteins by transferring sulfate from 3’-phosphoadenosine-5’-phosphosulfate to the hydroxyl group of protein-bound tyrosine residue, resulting in protein tyrosine O-sulfation [4, 5]. Targeted knockout of TPST2 caused male infertility due to reduced motility of sperms [6] and moderate primary hypothyroidism due to the lack of exocrine secretory granules [7]. Because the subcellular localization of TPST2 is trans-Golgi network, TPST2 exerts its enzyme activity to membrane and secretory proteins, and has been suggested to modulate the extracellular protein-protein interactions [8]. Therefore, it is plausible that TPST2 plays a role in the regulation of immune response by modulating extracellular protein-protein interactions [8].

In this study, we demonstrated that TPST2 modulates the responsiveness to ICT in cancer cells. To identify the tumor intrinsic factors that are associated with responsiveness to cancer immunotherapy, we performed genome-wide CRISPR-Cas9 loss-of-function screens of cancer cells using mice bearing human immune system and identified TPST2 as a therapeutic target to enhance anti-PD1 efficacy. Our data suggest a novel role of TPST2 in cancer immunity and provide an additional strategy for combination therapeutics based on ICT.

## Materials and methods

### In vivo genome-wide CRISPR screen

To generate lentiviral library for genome-wide knock-out screen, we adopted Human GeCKOv2 pooled library in 1 vector system, which was a gift from Feng Zhang (Addgene #1,000,000,048) [9]. MDA-MB-231 cells were infected with the lentivirus library with MOI of 0.3–0.5 for 24 h and selected by puromycin treatment (1 µg/mL) for 3 days. Puromycin-resistant cells (a minimum of 1 × 10^7^ cells for each mouse) were injected into the flanks of 4-week-old NOD/SCID/IL-2γ-receptor null (NSG) female mice with reconstituted human immune system. The sgRNA distribution in the injected cells was analyzed by amplicon sequencing (Fig. S1). Drug treatments began after tumors reached approximately 100 mm^3^. Mice were randomly divided into two treatment groups consisting of 2 mice in each group: control IgG and pembrolizumab (5 mg/kg, every 5 days), which were administered intraperitoneally for 27 days.

From the residual tumors, genomic DNA was purified with a Blood & Cell Culture Midi kit (Qiagen). The amplification of sgRNA target sequences for sequencing was performed as previous described with minor modifications [10]. For the first PCR, total 130 μg DNA per tumor was amplified using Herculase II Fusion DNA Polymerase (Agilent). Primer sequences to amplify lentiCRISPR sgRNAs for the first PCR are: v2Adaptor_F 5’-AATGGACTATCATATGCTTACCGTAACTTGAAAGTATTTCG-3’ and v2Adaptor_R 5’-TCTACTATTCTTTCCCCTGCACTGTTGTGGGCGATGTGCGCTCTG-3’. A second PCR was performed using 5 µl of the first PCR product to attach Illumina adaptors and barcodes. Primer sequences for second PCR were adopted as previously suggested [11]. The PCR amplicons were gel-extracted and sequenced using a HiSeq 2500 instrument (Illumina) in single-end mode.

Raw sequencing data were preprocessed using the FASTX toolkit (http://hannonlab.cshl.edu/fastx_toolkit/) which remove low quality reads using fastq_quality_filter. The resulting reads were trimmed to remove the constant portion of the sgRNA sequences with CRISPR.sgRNA_read_trimmer in the GenePattern Module Archive (http://www.gparc.org/), then aligned to the GeCKO v2 sgRNA library using bowtie 1 (v1.1.1) under default settings. After alignment, the number of uniquely aligned reads for each library sequence was calculated by CRISPR.single_sgRNA_count in GParc. The raw read counts were normalized as follows: normalized reads per sgRNA = (reads per sgRNA / total reads for all sgRNAs in sample) x 10^6^ + 1. The differentially enriched sgRNAs for each treatment group were calculated by *t*-test.

### Cell culture and generation of knock-down cells using CRISPR method

Human breast cell lines (MDA-MB-231 and MDA-MB-468) and mouse colon cancer cell line (MC38), authenticated using DNA fingerprint analysis, were provided by the Korean Cell Line Bank. Cells were passaged for fewer than 6 months after resuscitation. Cells were cultured in RPMI 1640 medium (Life Technologies) with 10% fetal bovine serum (Life technologies), penicillin (100 units/ml; Life Technologies) and streptomycin (100 units/ml; Life Technologies). All cells were maintained in a humidified incubator with 5% CO_2_ at 37 °C.

Knock-down cell lines for human TPST2 and mouse Tpst2 were generated using CRISPR method. The single guide RNAs for target genes were cloned into lentiCRISPR v2 (a gift from Feng Zhang, Addgene plasmid # 52,961). Lentivirus were generated by transfecting lentiCRISPR v2, pCMV-VSV-G, and psPAX2 into 293FT cells. Target cells were transduced with lentivirus, which were concentrated using conditioned media from transfected 293FT cells, for 48 h and selected by puromycin treatment (1 µg/mL for MDA-MB-231 and MDA-MB-468, 2 µg/mL for MC38) for 3 days. The knock-down of target genes were evaluated by quantitative real-time PCR. The sgRNA sequences for each target gene were summarized in Supplementary Table S1.

### Real-time PCR

Total RNA from cells was purified using RNeasy Plus Mini Kit (Qiagen). Reverse transcription with 1 µg of total RNA was performed using Maxime RT PreMix (Intron Biotechnology). We performed real-time PCR using SYBR^®^ Green Master Mix (Bio-rad) and estimated mRNA level normalized by GAPDH used as internal control. The sequences of primers were summarized in Supplementary Table S1.

### Western blotting

Cells were lysed using RIPA buffer (Thermo Scientific) containing protease inhibitor cocktail (Roche) and phosphatase inhibitor cocktail (Roche), incubated for 15 min in ice and centrifuged at 16,800 g for 10 min at 4 °C. BCA assay (Thermo Scientific) was used to estimate the protein concentration. Proteins were resolved by SDS-PAGE and transferred to nitrocellulose membrane. After blocking with 5% skim milk, membranes were probed with anti-phospho-STAT1 (Cell Signaling Technology, Cat. No. 7649), anti-STAT1 (Cell Signaling Technology, Cat. No.14,994), anti-TPST2 (Atlas Antibodies, Cat. No. HPA021054), anti-sulfotyrosine (Abcam, Cat. No. ab136481), anti-myc-tag (Cell Signaling Technology, Cat. No. 2276), anti-flag (Sigma-Aldrich Corporation, Cat. No. F1804), anti-IFNGR1 (Cell Signaling Technology, Cat. No. 10,405), and anti-actin (Sigma-Aldrich Corporation, Cat. No. 5441) antibody. The membranes were probed with horseradish peroxidase-conjugated secondary antibody. The proteins were visualized by enhanced chemiluminescence development according to the manufacturer’s instructions (Pierce).

### Cell proliferation assay

Cell proliferation in response to IFNγ treatment was estimated by trypan blue staining assay. Cells were harvested at indicated times and incubated with 0.4% solution of trypan blue dye (Thermo Scientific). The numbers of viable cells were counted using hemocytometer.

### Gene set enrichment analysis (GSEA)

GSEA was performed using javaGSEA desktop application (GSEA v2.1.0) [12]. The hallmark gene sets were used to investigate the enriched gene sets for each group. The *P*-values were calculated by permuting the data 1000 times for finding enriched gene sets. The GSEA software produces enrichment score (ES), normalized ES (NES), nominal P-value, and false discovery rate (FDR; Q-value). Gene sets that were up- or down-regulated with a P-value < 0.05 were considered significant.

### Immunoprecipitation

Cells were washed twice with ice-cold PBS and lysed in lysis buffer (50 mM Tris-Cl, pH 8.0, 150 mM NaCl, 1% NP-40, protease inhibitor cocktail (Roche), phosphatase inhibitor cocktail (PhosSTOP; Roche)) for 30 min. Cell lysates were centrifuged at 12,000 g for 20 min at 4 °C, and supernatants were incubated with anti-myc-tag (Cell Signaling Technology, Cat. No. 2276), anti-flag (Sigma-Aldrich Corporation, Cat. No. F1804) or normal IgG antibody (Cell Signaling Technology, Cat. No. 61,656), which were previously coupled with protein G-conjugated magnetic beads (Dynabeads; Thermo Scientific), at 4 °C for 16 h. The beads were washed five times with lysis buffer. Proteins were eluted in sample loading buffer by boiling for 10 min and detected by western blot analysis.

### Site-directed mutagenesis

Site-directed mutagenesis of IFNGR1 was performed according to QuikChange site-directed mutagenesis kit (Stratagene, La Jolla, CA, USA) instruction manual. The primers for IFNGR1 Y397F mutation were as follows; 5’-CGCTTTAAACTCGT*T*TCACTCCAGAAATTG-3’ and 5’-CAATTTCTGGAGTGA*A*ACGAGTTTAAAGCG-3’. The mutated sequences are italicized.

### Syngeneic mouse tumor model

All animal experiments were carried out in accordance with protocols approved by the Institutional Animal Care and Use Committee of GIST (IACUC no. GIST-2020-085 and GIST-2023-011). All animals used in this study were maintained and handled according to the policies approved by GIST. Female C57B6/N mice or nude mice (5 weeks old) were provided by Orient Bio (Gapyeong, Gyeonggi, Korea). For tumor growth experiments, mice were injected subcutaneously with 2 × 10^5^ MC38 colon cancer cells. One week after inoculating the tumors, we injected the tumor-bearing mice intraperitoneally with 2 mg/kg anti-PD-1 mAb (clone RMP1-14, BioXCell, USA) in PBS on days 3, 7, 10, 14, and 17. Tumor size was measured three times a week until the endpoint, and tumor volume was calculated as length × width^2^ × 0.5.

### Flow cytometry analysis

Tumor tissues and tumor-draining lymph nodes were harvested at day 15 after MC38 tumor cell inoculation. Tumor tissues were cut into small pieces and transferred in RPMI 1640 media (Corning Incorporated, Cat. No. 10-040-CV, Corning, New York, USA) supplemented with 2.5 mg/ml collagenase type 1 (Gibco, Cat. No. 17018-029), 1.5 mg/ml collagenase type 2 (Gibco, Cat. No. 17101-015), 1 mg/ml collagenase type 4 (Gibco, Cat. No. 17104-019), 50 µg/ml DNase type 1 (Merck, Cat. No. 11,284,932,001), and 0.25 mg/ml hyaluronidase Type IV-S (Sigma Aldrich, Cat. No. H3884). These tumor samples were incubated for 40 min at 37 °C with 150 rpm, and then filtered through a 70-µm cell strainer (Falcon, Cat. No. 352,350). Tumor-draining lymph node was grinded using 3 mL syringe plunge and filtered through 70-µm cell strainer. These samples were stained by anti-mouse CD16/32 antibody for Fc receptor blocking. Anti-mouse CD45, CD3, CD4, CD8a, CD44, CD62L, CD25, Foxp3, NK1.1, CD11c, CD11b, B220, F4/80, iNOS, and CD206 antibodies (Biolegend or BD Bioscience) were used for cell staining. Stained cell acquisition was performed with CANTO II (BD Bioscience), and data analysis was performed using the FlowJo software (TreeStar, San Carlos, CA, USA). Gating strategy for immune cell profiling is described in Fig. S2.

### Analysis of single cell RNA sequencing data of public lung cancer and glioma cohorts

We conducted a comprehensive single-cell RNA sequencing (scRNA-seq) analysis on human lung tumor tissues to investigate TPST2 expression and its related changes at the single-cell level. Using data from a public lung cancer cohort (Code Ocean capsule: 10.24433/CO.0121060.v1), we loaded scRNA-seq data for 12 patients into Seurat, ensuring data quality by filtering cells with less than 10% mitochondrial gene content. After normalization, identification of variable features, and integration using Seurat’s FindIntegrationAnchors and IntegrateData functions, we identified 13 unique clusters through unsupervised clustering and annotated these clusters based on their most distinct markers (Supplementary Table S2). We then categorized cells into immune (e.g., B cells, T cells, NK cells, and dendritic cells) and non-immune (e.g., endothelial cells, epithelial cells, and fibroblasts) types. For detailed analysis, non-immune cells were split into TPST2-negative and TPST2-positive groups. We used the FindMarkers function to identify differentially expressed genes and performed GSEA with the WebGestalt tool to understand enriched pathways in TPST2-negative non-immune cells. Additionally, we performed gene scoring with tumor markers (EPCAM, KRT8, KRT18, MUC1, TP53, CEACAM5, and SOX2) and lung cancer-specific markers (TTF1, NAPSA, and CDH1) to distinguish tumor cells from non-tumor cells. Finally, we used CellChat to examine interactions between tumor and non-tumor cells within the TPST2-negative non-immune cell population. We also analyzed scRNA-seq data from a glioma cohort (10.1038/s41467-022-28372-y), focusing on non-immune cells and categorizing them into TPST2-negative and TPST2-positive groups. We identified differentially expressed genes between these groups using the FindMarkers function and conducted GSEA with the WebGestalt tool. Additionally, we performed over-representation analysis using the clusterProfiler, ReactomePA, and org.Hs.eg.db packages.

### Statistical analysis

Statistical analyses were performed using Prism 9.3.1 (GraphPad) or R software (version 4.2.1). Differences between two variables and multiple variables were assessed using the Student’s t-test and ANOVA with Tukey’s multiple comparison test, respectively. Differences were considered significant if the P-value was less than 0.05, except sgRNA selection from genome-wide CRISPR screening (*P* < 0.1) and KEGG pathway analysis with selected genes from genome-wide CRISPR screening (*P* < 0.2). All statistical methods and significance thresholds are described in the corresponding figure legends.

## Results

### In vivo CRISPR screens identified tumor cell-intrinsic pathways associated with anti-PD1 responsiveness

**Fig. 1:**
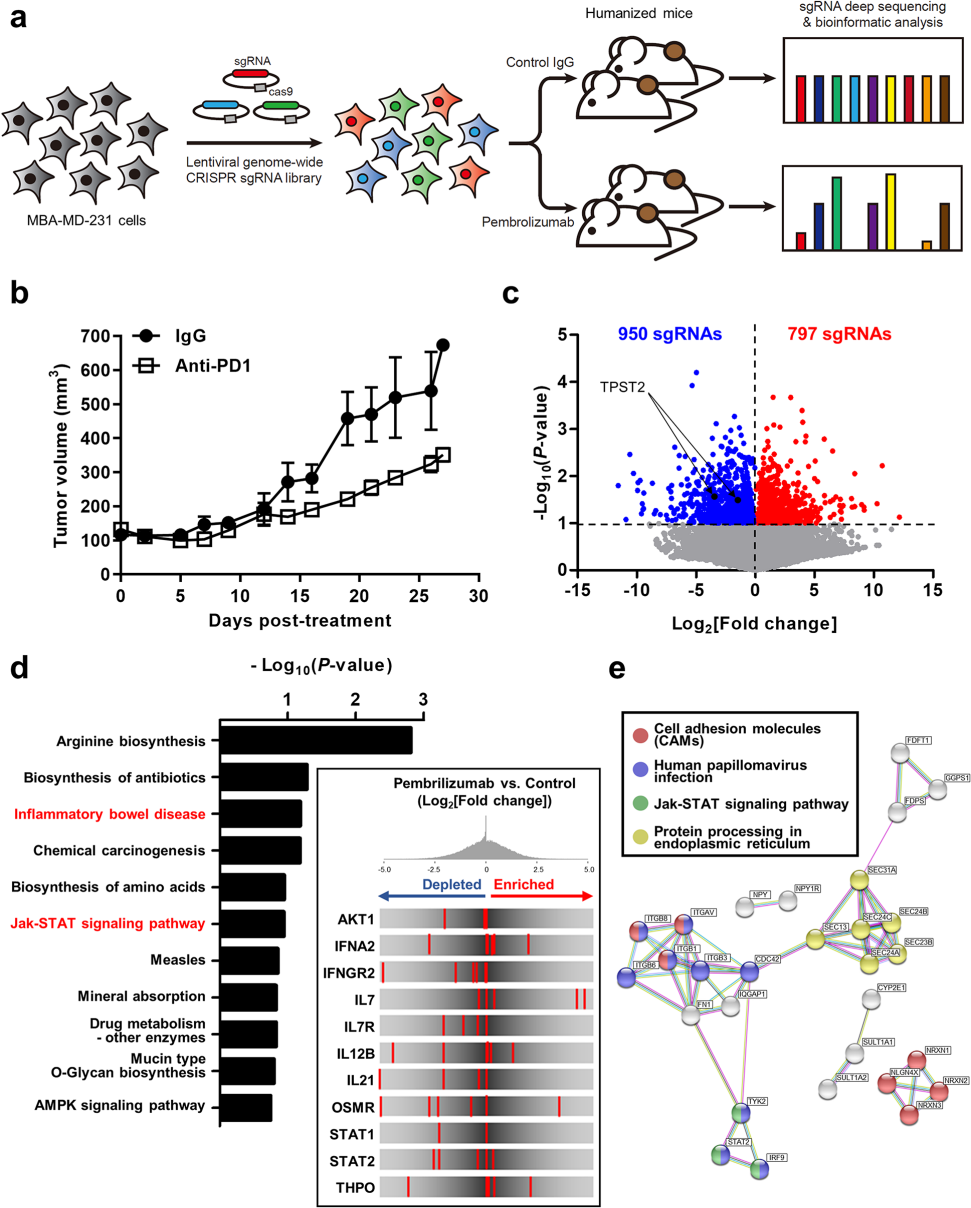
In vivo genome-wide CRISPR/Cas9 knockout screening for anti-PD1 responsiveness. **a**, Schematic of the in vivo genome-wide CRISPR/Cas9 knockout screens to identify genes associated with anti-PD1 responsiveness. MDA-MB-231 cells were infected with the lentivirus with human GeCKO v2 sgRNA library, injected into humanized NOD/SCID/IL-2γ-receptor null (NSG) mice, and treated with control IgG or pembrolizumab. The abundance of each sgRNA in each residual tumor was determined by next-generation sequencing. **b**, The in vivo efficacy of pembrolizumab in humanized NSG mice with xenografts of MDA-MB-231 cells. The mice were treated with control IgG or pembrolizumab (*n* = 2, 5 mg/kg, every 5 days) for 27 days, and average tumor sizes for each group are plotted. **c**, Volcano plot illustrating the relative enrichment of sgRNAs in the genome-wide CRISPR/Cas9 knockout screen for anti-PD1 responsiveness. The combined results of two biological replicates from the screening are represented. Total 950 sgRNAs and 797 sgRNAs were depleted and enriched in anti-PD1-treated tumors (*P* < 0.1), respectively. **d**, KEGG pathway analysis of 918 genes, sgRNAs of which were depleted in anti-PD1-treated tumors. The KEGG pathways that were significantly enriched in the 918 genes (*P* < 0.2) are shown. The inlet represents the rank distribution diagram of sgRNAs targeting genes associated with Jak-STAT signaling pathway from 918 genes. **e**, STRING network analysis with 22 genes with multiple depleted sgRNAs in anti-PD1-treated mice. Genes involved in each pathway are marked with the corresponding color

To investigate tumor cell-intrinsic factors that determine anti-PD1 responsiveness, we performed a pooled loss-of-function genetic screen using humanized mice in which human immune cells were recapitulated via engraftment of CD34^+^ hematopoietic stem cells [13] (Fig. 1a). We transduced breast cancer cells (MDA-MB-231) with a library of lentivirus encoding Cas9 and 123,411 sgRNAs targeting 19,050 genes [9], and transplanted the lentivirus-infected cells into humanized mice. When we treated the mice with anti-PD1 antibody (pembrolizumab), in vivo tumor growth was retarded compared to control mice (Fig. 1b). Because the presence of sgRNA results in the inactivation of a matched gene and the prevalence of sgRNA inducing the resistance to anti-PD1 increases in residual tumors, we analyzed the frequencies of sgRNAs in control and anti-PD1-treated residual tumors (Fig. 1a). Compared to control tumors, total 797 sgRNAs for 777 genes were enriched in anti-PD1-treated tumors (*P* < 0.1), indicating that loss-of-function of these genes contributed to anti-PD1 resistance. Conversely, total 950 sgRNAs for 918 genes were depleted in anti-PD1-treated tumors (*P* < 0.1), suggesting that loss-of-function of these genes increased sensitivity to anti-PD1 (Fig. 1c). KEGG pathway analyses using 918 genes showed the enrichment of several immune-related pathways including ‘Inflammatory bowel disease’ and ‘Jak-STAT signaling pathway’ (*P* < 0.2), and especially, 11 genes were found in ‘Jak-STAT signaling pathway’ (Fig. 1d). We selected 22 genes with multiple depleted sgRNAs in anti-PD1-treated mice (Fig. S3) and mapping these genes onto the STRING protein-protein interaction networks also demonstrated a highly connective network enriched in immune-related pathways, including ‘Cell adhesion molecules (CAMs)’, ‘Human papillomavirus infection’, and ‘Jak-STAT signaling pathway’ (*P* < 0.05, Fig. 1e). In addition, ‘Protein processing in endoplasmic reticulum’ pathway was also enriched in protein-protein interaction network (Fig. 1e). Pathway analyses using 777 genes, whose loss-of-function increased anti-PD1 resistance, also showed the enrichment of ‘Jak-STAT signaling pathway’ and ‘Cytokine-cytokine receptor interaction’ (Fig. S4a-c). These data suggest that ‘Jak-STAT signaling pathway’ and ‘Cytokine-cytokine receptor interaction’ are highly associated pathways with the anti-PD1 responsiveness.

### Targeting TPST2 enhanced IFNγ signaling pathway in breast cancer cells

In in vivo screening for anti-PD1 responsiveness, TPST2 is one of 22 genes with multiple depleted sgRNAs in anti-PD1-treated mice and the frequency of sgRNAs targeting TPST2 tended to decrease in anti-PD1-treated mice compared to control mice (Fig. S5a), which suggests that inactivation of TPST2 rendered cells more susceptible to anti-PD1 treatment. In addition, mRNA expression levels of TPST2 were significantly reduced in anti-PD1-treated melanoma patients with complete response compared to patients with progressive disease or partial response [14] (Fig. S5b), and in anti-PD-L1 (atezolizumab)-treated urothelial cancer patients with partial or complete response compared to progressive or stable disease [15] (*P* = 0.004; Fig. S5c).

**Fig. 2:**
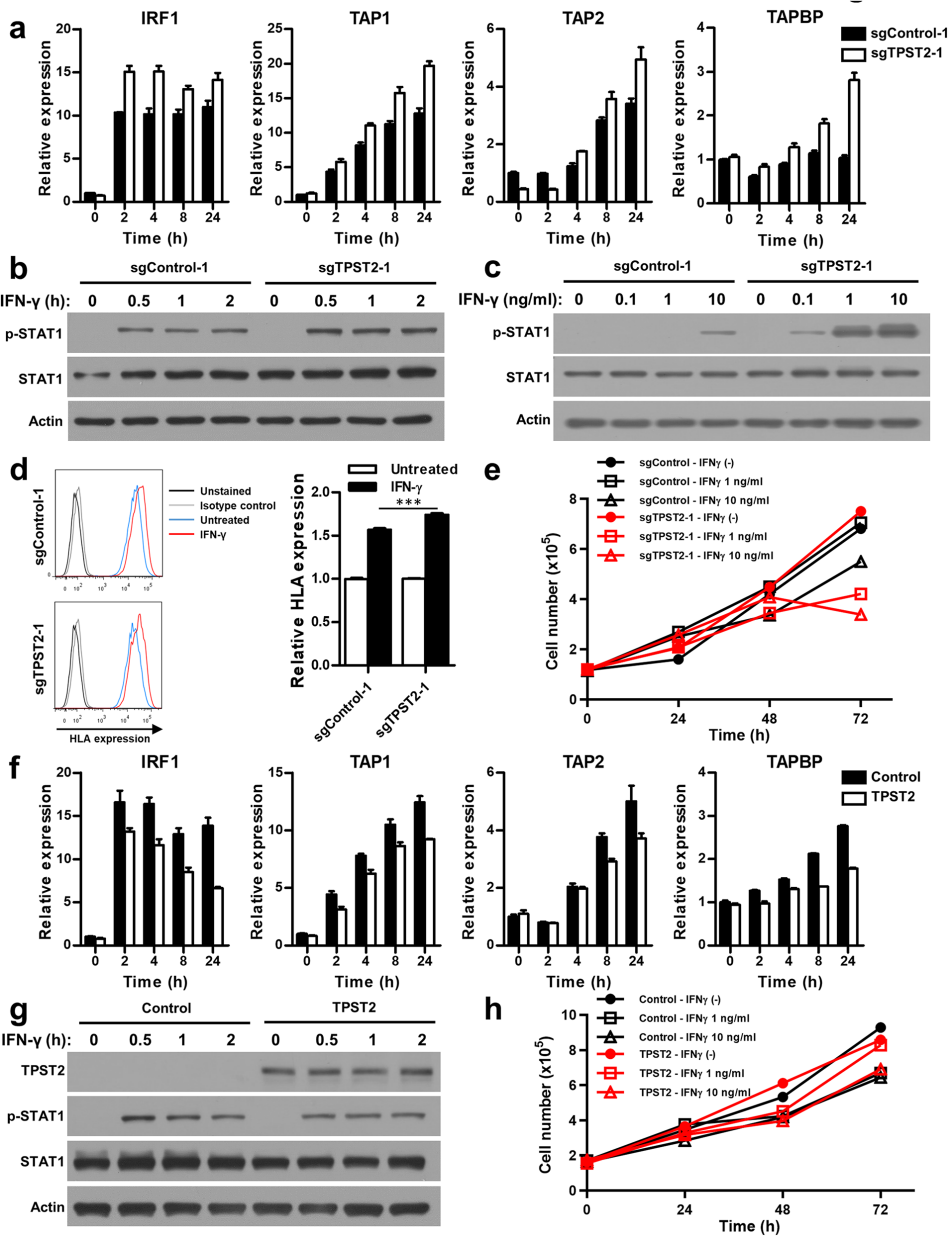
TPST2-mediated suppression of IFNγ signaling pathway in breast cancer cells. **a**, Enhanced expression of IFNγ-responsive genes in TPST2-depleted breast cancer cells. TPST2 was knocked down using CRISPR/Cas9 in MDA-MB-231 cells. After serum starvation for 24 h, cells were treated with 10 ng/ml IFNγ and the expression levels of IFNγ-responsive genes were estimated by real-time PCR at indicated time points. **b**, **c**, Enhanced phosphorylation of STAT1 in TPST2-depleted breast cancer cells. After serum starvation for 24 h, cells were treated with IFNγ for indicated time (**b**) and with indicated concentration (**c**). The phosphorylation levels of STAT1 were evaluated by western blotting. **d**, Enhanced expression of human leukocyte antigen (HLA) by IFNγ treatment in TPST2-depleted breast cancer cells. After serum starvation for 24 h, cells were treated with 10 ng/ml IFNγ for 24 h and the expression levels of IFNγ-responsive HLA were estimated by flow cytometry. **e**, Enhanced suppression of cell proliferation by IFNγ treatment in TPST2-depleted breast cancer cells. After serum starvation for 24 h, cells were treated with 1 or 10 ng/ml IFNγ for indicated time points and the cell numbers were estimated by trypan blue staining assay. **f**, Reduced expression of IFNγ-responsive genes in TPST2-overexpressed breast cancer cells. TPST2 was overexpressed in MDA-MB-231 cells for 24 h. After serum starvation for 24 h, cells were treated with 10 ng/ml IFNγ and the expression levels of IFNγ-responsive genes were estimated by real-time PCR at indicated time points. **g**, Reduced phosphorylation of STAT1 in TPST2-overexpressed breast cancer cells. After serum starvation for 24 h, cells were treated with 1 ng/ml IFNγ for indicated time. The phosphorylation levels of STAT1 were evaluated by western blotting. **h**, Reduced suppression of cell proliferation by IFNγ treatment in TPST2-overexpressed breast cancer cells. After serum starvation for 24 h, cells were treated with 1 or 10 ng/ml IFNγ for indicated time points and the cell numbers were estimated by trypan blue staining assay

Our genome-wide CRISPR screens suggest that ‘Jak-STAT signaling pathway’ is highly associated with anti-PD1 responsiveness, and altered IFNγ signaling pathway has been implicated as a molecular mechanism underlying resistance to cancer immunotherapy [16]. Therefore, we investigated the knock-down effect of TPST2 on IFNγ signaling pathway. We generated TPST2 knock-down MDA-MB-231 cells using CRISPR/Cas9 method (Fig. S5d). Down-regulation of TPST2 enhanced the expression of IFNγ-responsive genes including *IRF1*, *TAP1*, *TAP2*, and *TAPBP* [17] in response to IFNγ treatment (Fig. 2a, Fig. S5e), and increased the phosphorylation of STAT1 after IFNγ stimulation (Fig. 2b, c). In addition, down-regulation of TPST2 augmented IFNγ-induced human leukocyte antigen (HLA) expression (Fig. 2d), which is associated with antigen presentation, and IFNγ-mediated growth inhibition (Fig. 2e). However, knock-down of TPST2 had little effect on the cell proliferation in the absence of IFNγ (Fig. S5f). Concurrently with these data, overexpression of TPST2 in MDA-MB-231 cells inhibited the expression of IFNγ-responsive genes and phosphorylation of STAT1 after IFNγ treatment (Fig. 2f, g, Fig. S5g). Overexpression of TPST2 also diminished IFNγ-induced HLA expression (Fig. S5h), and IFNγ-mediated growth inhibition, especially in response to 1 ng/ml IFNγ treatment (Fig. 2h). However, active site mutant of TPST2 (R101A) [5] failed to inhibit IFNγ signaling, which was estimated by the phosphorylation of STAT1 (Fig. S5i). The inhibitory role of TPST2 in IFNγ signaling was verified in another breast cancer cell line, MDA-MB-486 (Fig. S6a-e). Collectively, these findings indicate that TPST2 exerts inhibitory effects on IFNγ signaling in breast cancer cells.

In humans, two TPST isoforms, designated as TPST1 and TPST2, are expressed [18]. TPST1 and TPST2 show 64% amino acid sequence similarity and differential substrate specificity and tissue-specific expressions [19, 20]. When we evaluated the roles of TPST1 in IFNγ signaling, knock-down of TPST1 did not increase, but slightly inhibited cellular responsiveness to IFNγ, in terms of the expression of IFNγ-responsive genes, the phosphorylation of STAT1, HLA expression, and cell growth inhibition in response to IFNγ treatment (Fig. S7a-e). These data suggest distinct roles of TPST1 in IFNγ signaling compared to TPST2.

### Targeting TPST2 induced transcriptomic reprogramming in the IFNγ responses of breast cancer cells

To investigate the role of TPST2 in the global gene expressions in response to IFNγ treatment, we examined the transcriptome of TPST2 knock-down cells following IFNγ stimulation using RNA sequencing. In the presence of IFNγ, knock-down of TPST2 significantly up- and down-regulated 313 genes and 225 genes compared with control cells, respectively (*P* < 0.05, Log_2_[fold change] ≥ 1; Fig. S8a). The gene set analyses using the up-regulated 313 genes demonstrated that several immune-related gene sets such as ‘INFLAMMATORY_RESPONSE’ from hallmarks gene set (https://www.gsea-msigdb.org/gsea/msigdb/index.jsp) [21] and ‘CYTOKINE_CYTOKINE_RECEPTOR_INTERACTION’ from KEGG pathway gene set (https://www.genome.jp/kegg/) [22] were enriched in TPST2 knock-down cells (Fig. S8b, c). In addition, the genes in the hallmarks gene set ‘Apoptosis’ were enriched in TPST2 knock-down cells (Fig. S8b), which is suggestive of enhanced IFNγ signaling, because IFNγ signaling is known to exert pro-apoptotic effects on cancer cells [23].

Gene set enrichment analysis (GSEA) [12] also showed that IFNγ signaling-related gene sets from hallmarks gene sets, including ‘INFLAMMATORY_RESPONSE’, ‘INTERFERON_GAMMA_RESPONSE’, and ‘APOPTOSIS’ were enriched in TPST2 knock-down cells compared to control cells (Fig. S8d). In GSEA, TPST2 knock-down resulted in the depletion of cell cycle-related genes, such as ‘E2F_TARGETS’, ‘G2M_CHECKPOINT’, ‘MYC_TARGETS’, and ‘MITOTIC_SPINDLE’ (Fig. S8e), which is suggestive of enhanced IFNγ signaling, because IFNγ signaling is involved in the anti-proliferative effect on the cancer cells [23].

Antigen presentation by HLA molecules is required for the proper execution of ICT [24], and IFNγ increases the amount and efficiency of antigen presentation via HLA complexes [25]. Our transcriptome analyses demonstrated that, in the presence of IFNγ, a series of major histocompatibility complex (MHC) class I genes, including HLA-A, HLA-B, HLA-C, HLA-E, and HLA-H, were up-regulated in the TPST2 knock-down cells (Fig. S8f). MHC class I molecules expressed by cancer cells are recognized by CD8^+^ cytotoxic T lymphocytes (CTLs), which are major players in ICT [26]. Taken together, knock-down of TPST2 enhanced the IFNγ-induced reprogramming of gene expression at the transcriptome level.

### TPST2-mediated tyrosine sulfation of IFNγ receptor 1 (IFNGR1) modulates IFNγ signaling in breast cancer cells

**Fig. 3:**
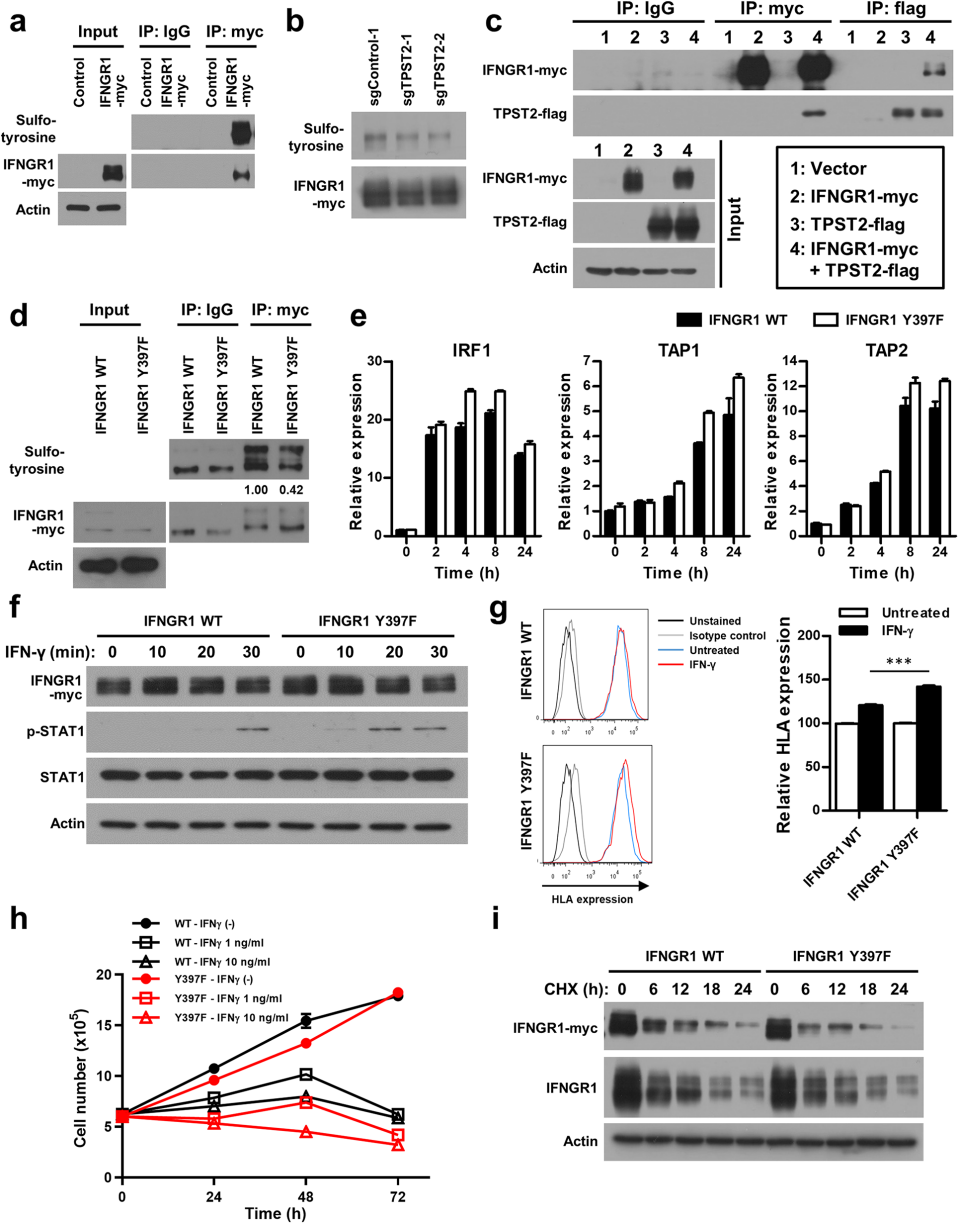
Modulation of IFNγ signaling via TPST2-mediated tyrosine sulfation of IFNγ receptor 1 (IFNGR1) in breast cancer cells. **a**, Detection of IFNGR1 tyrosine sulfation in MBA-MD-231 breast cancer cells. Myc-tagged IFNGR1 was overexpressed in MBA-MD-231 cells. IFNGR1 was immunoprecipitated by Myc antibody and tyrosine sulfation of IFNGR1 was detected by antibody for sulfotyrosine. **b**, Reduced sulfotyrosine levels of IFNGR1 in TPST2 knock-down cells. Myc-tagged IFNGR1 was overexpressed in control and TPST2 knock-down MBA-MD-231 cells. IFNGR1 was immunoprecioitated by Myc antibody and tyrosine sulfation of IFNGR1 was detected by antibody for sulfotyrosine. **c**, Protein-protein interaction between TPST2 and IFNGR1. Flag-tagged TPST2 and/or myc-tagged IFNGR1 was overexpressed in MBA-MD-231 cells. Complex formation of TPST2 and IFNGR1 was evaluated by co-immunoprecipitation of flag-tagged TPST2 and myc-tagged IFNGR1, which was detected by reciprocal immunoprecipitation and western blotting. **d**, Reduced tyrosine sulfation of mutant IFNGR1. Myc-tagged wild-type and Y397F mutant IFNGR1 was overexpressed in MBA-MD-231 cells. IFNGR1 was immunoprecioitated by Myc antibody and tyrosine sulfation of IFNGR1 was detected by antibody for sulfotyrosine. The relative sulfotyrosine western band intensity was evaluated by image J software and depicted under the sulfotyrosine western data. **e**, Enhanced expression of IFNγ-responsive genes in mutant IFNGR1-overexpressed breast cancer cells. Wild-type and mutant (Y397F) IFNGR1 were overexpressed in MDA-MB-231 cells for 24 h. After serum starvation for 24 h, cells were treated with 10 ng/ml IFNγ and the expression levels of IFNγ-responsive genes were estimated by real-time PCR at indicated time points. **f**, Enhanced phosphorylation of STAT1 in mutant IFNGR1-overexpressed breast cancer cells. Wild-type and mutant (Y397F) IFNGR1 were overexpressed in MDA-MB-231 cells for 24 h. After serum starvation for 24 h, cells were treated with IFNγ for indicated time. The phosphorylation levels of STAT1 were evaluated by western blotting. **g**, Enhanced expression of human leukocyte antigen (HLA) by IFNγ treatment in mutant IFNGR1-overexpressed breast cancer cells. Wild-type and mutant (Y397F) IFNGR1 were overexpressed in MDA-MB-231 cells for 24 h. After serum starvation for 24 h, cells were treated with 10 ng/ml IFNγ for 24 h and the expression levels of IFNγ-responsive HLA were estimated by flow cytometry. **h**, Enhanced suppression of cell proliferation by IFNγ treatment in mutant IFNGR1-overexpressed breast cancer cells. Wild-type and mutant (Y397F) IFNGR1 were overexpressed in MDA-MB-231 cells for 24 h. After serum starvation for 24 h, cells were treated with 1 or 10 ng/ml IFNγ for indicated time points and the cell numbers were estimated by trypan blue staining assay. **i**, Protein stability of mutant IFNGR1. Wild-type and mutant (Y397F) IFNGR1 were overexpressed in MDA-MB-231 cells for 24 h. After treatment of cycloheximide (100 µg/ml) to inhibit protein translation, IFNGR1 protein levels of were evaluated by western blotting at indicated time points

Next, we investigated the target substrate of TPST2 in the regulation of IFNγ signaling. By analyzing two prediction models for protein tyrosine sulfation, Sufinator (https://web.expasy.org/sulfinator/) [27] and SulfoSite (http://sulfosite.mbc.nctu.edu.tw/) [28], we found that interferon gamma receptor 1 (IFNGR1) was a candidate target for tyrosine sulfation (Fig. S9a, b), but no such sites were predicted for IFNGR2. The purified IFNGR1 proteins via immunoprecipitation were detected by anti-sulfotyrosine antibodies (Fig. 3a), and knock-down of TPST2 reduced the sulfotyrosine levels of IFNGR1 (Fig. 3b). In addition, the interaction between TPST2 and IFNGR1 was verified by co-immunoprecipitation experiment (Fig. 3c), suggesting that IFNGR1 is a direct substrate of TPST2.

We explored the IFNGR1 target residue of TPST2-mediated tyrosine sulfation by mass spectrometry. When we analyzed peptides from purified IFNGR1 by anti-myc antibody, sulfation of Y397 and phosphorylation of several residues were detected (Fig. S10a-c). To validate the effect of sulfated Y397 on IFNγ signaling, we generated IFNGR1 knock-out cells using CRISPR method and overexpressed wild-type or Y397F mutant IFNGR1 (Fig. S10d). The level of sulfotyrosine decreased in Y397F mutant IFNGR1 compared to wild-type, but was not completely abolished in mutant IFNGR1 (Fig. 3d), probably due to other sulfotyrosines predicted by prediction models (Fig. S9a, b). Compared to wild-type IFNGR1, overexpression of Y397F mutant IFNGR1 enhanced cellular responsiveness to IFNγ, estimated by the expression of IFNγ-responsive genes (Fig. 3e), the phosphorylation of STAT1 (Fig. 3f), induction of HLA expression (Fig. 3g), and IFNγ-mediated growth inhibition (Fig. 3h). However, the protein half-life was not significantly different between wild-type and Y397F mutant IFNGR1 (Fig. 3i). These data suggest that TPST2-mediated sulfation of IFNGR1 at Y397 impedes the responsiveness to IFNγ in cancer cells.

### Depletion of TPST2 augmented the anti-cancer immunity mediated by anti-PD1 antibodies

**Fig. 4:**
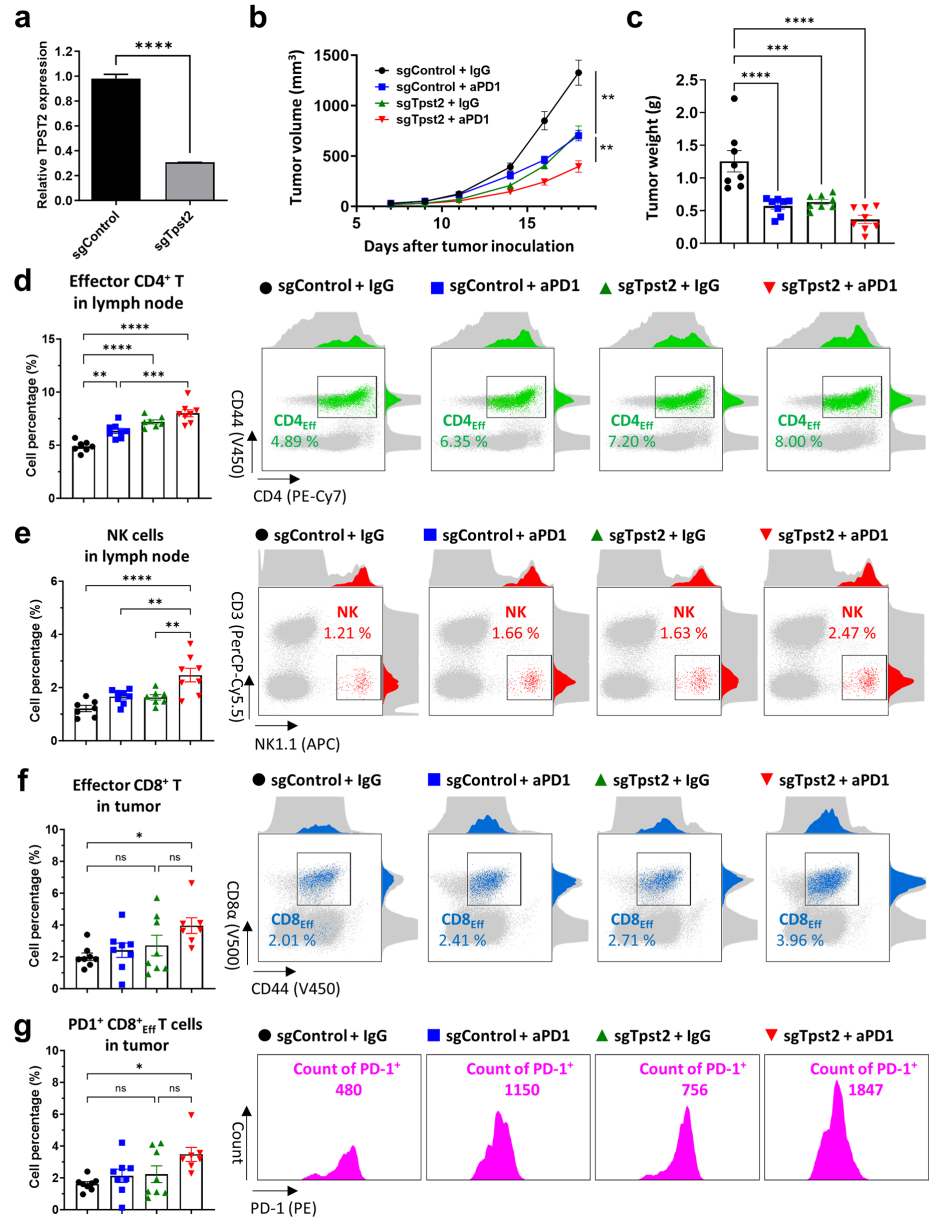
TPST2 knock-down enhances anti-PD1 efficacy via activating T cell immunity. **a**, Relative mouse *Tpst2* gene expression in control (sgControl) and Tpst2 knock-down MC38 cells (sgTpst2) estimated by RT-PCR. **b**, Representative control MC38 or Tpst2 knock-down MC38 tumor growth curves with or without anti-PD1; *n* = 8 mice per group. **c**, Tumor weight at 18 days after tumor injection in syngeneic mouse model; *n* = 8 mice per group. **d**, Percentage of effector CD4^+^ T cell in tumor-draining lymph nodes by flow cytometry analysis from control MC38 or Tpst2 knock-down MC38-bearing mice with or without anti-PD1; *n* = 8 mice per group (left). The dot-plot represents population of effector CD4^+^ T cell through CD4 and CD44 expression in each group (right). Gray background represents total immune cells in tumor-draining lymph node and green dot represents effector CD4^+^ T cell. The above-attached and right-attached histograms represent CD4 expression and CD44 expression of each group, respectively. **e**, Percentage of NK cell in tumor-draining lymph nodes by flow cytometry analysis from control MC38 or Tpst2 knock-down MC38-bearing mice with or without anti-PD1; *n* = 8 mice per group (left). The dot-plot represents population of NK cell through CD3 and NK1.1 expression in each group (right). Gray background represents total immune cells in tumor-draining lymph nodes and red dot represents NK cell (CD3^-^ NK1.1^+^). The above-attached and right-attached histograms represent NK1.1 expression and CD3 expression of each group, respectively. **f**, Percentage of effector CD8^+^ T cell in tumor tissues by flow cytometry analysis from control MC38 or Tpst2 knock-down MC38-bearing mice with or without anti-PD1; *n* = 8 mice per group (left). The dot-plot represents population of effector CD8^+^ T cell through CD8 and CD44 expression in each group (right). Gray background represents total immune cells and blue dot represents effector CD8^+^ T cell. The above-attached and right-attached histograms represent CD8 expression and CD44 expression of each group, respectively. **g**, Percentage of PD1^+^ effector CD8^+^ T cell in tumor tissues by flow cytometry analysis from control MC38 or Tpst2 knock-down MC38-bearing mice with or without anti-PD1; *n* = 8 mice per group (left). The histogram represents PD1 expression in effector CD8^+^ T cell in each group (right)

We explored the effects of TPST2 knock-down on tumor growth and the host immune system in a syngeneic mouse model using MC38 cells (mouse colon cancer cells), which were reportedly sensitive to anti-PD1 treatment [29]. The Tpst2 knock-down cells exhibited similar proliferative capacity compared with control cells (Fig. 4a, Fig. S11a). However, down-regulation of Tpst2 enhanced the expression of IFNγ-responsive genes including Cxcl9, Cxcl10, and Tapbp in response to IFNγ treatment (Fig. S11b) and increased the phosphorylation of Stat1 after IFNγ stimulation (Fig. S11c). The effect of Tpst2 down-regulation on IFNγ responsiveness was also observed in mouse breast cancer 4T1 cells (Fig. S11d, e). We then assessed tumor growth changes in C57BL/6 mice injected subcutaneously with MC38 cell lines, following treatment with either anti-PD1 or IgG isotype control. Tpst2 knock-down alone significantly reduced tumor growth and notably enhanced the efficacy of anti-PD1 therapy (Fig. 4b), suggesting Tpst2 inhibition combined with anti-PD1 as a novel therapeutic strategy in oncology. Further analysis showed a significant reduction in tumor weight with Tpst2 knock-down and a trend towards decreased tumor weight with the combination treatment (Fig. 4c). These effects were abolished in cell xenograft models in nude mice (Fig. S11f), in which treatment of anti-PD1 also showed little effect on the tumor growth due to the depletion of mature T cells [30].

Given TPST2’s known impact on IFN-γ signaling in vitro experiments, we attributed the observed in vivo tumor growth reduction to immune-mediated mechanisms. We observed a significant increase in effector CD4^+^ T cells in the tumor-draining lymph nodes, particularly in the group treated with both Tpst2 knock-down and anti-PD1 (Fig. 4d). Concurrently, NK cells, critical for their cytotoxic capabilities, also peaked in this combination group compared to others in tumor-draining lymph nodes (Fig. 4e). Anti-PD1 therapy was found to increase the overall T cell population as a percentage of the total immune cell count within tumor tissues, with a pronounced increase in the Tpst2 knock-down + anti-PD1 group (Fig. S12a). While CD4^+^ T cell populations showed no significant differences across groups, CD8^+^ T cells increased with anti-PD1 treatment, further augmented by Tpst2 knock-down in the tumor tissues (Fig. S12b-d). The combined treatment group showed a significant rise in effector CD8^+^ T cell percentages compared to controls (Fig. 4f), along with a marked increase in PD1 expression levels within this group in the tumor tissues (Fig. 4g). The augmentation of anti-PD1 treatment efficacy by Tpst2 inhibition was confirmed through supplementary independent experiments, validating its impact on tumor reduction and enhancement of immune response. Tpst2 knock-down combined with anti-PD1 treatment led to decreased tumor volume and weight versus anti-PD1 monotherapy (Fig. S13a, b). Flow cytometry analysis revealed an increase in total T cells, including both CD4^+^ and CD8^+^ T cells, as well as effector CD4^+^ and CD8^+^ T cells as percentages of the total immune cell population in the Tpst2 knock-down + anti-PD1 group compared to the anti-PD1 monotherapy group (Fig. S13c-g). Overall, these findings highlight that Tpst2 knock-down enhances immune activation, particularly augmenting T cell immunity.

Beyond T cell immunity, we explored changes in myeloid lineage immune cells within tumor tissues. The Tpst2 knock-down + anti-PD1 combination group showed an increase in conventional type 1 dendritic cell (cDC1) population and a decrease in plasmacytoid dendritic cell (pDC) population (Fig. S14a-c). With cDC1 known to support anti-tumor responses and pDC involved in viral pathogen defense, these results align with previous findings that a higher cDC1/pDC ratio correlates with better anti-PD1 responsiveness [31, 32]. Furthermore, macrophage population analysis revealed no significant differences in overall percentage, but did show an increase in iNOS expression, indicating enhanced M1 polarization in the combination group (Fig. S14d, e). This polarization is crucial for anti-tumor effects and anti-PD1 therapy efficacy, reinforcing the potential of Tpst2 knock-down to boost anti-PD1 treatment outcomes.

### RNA sequencing and single-cell RNA sequencing revealed the enhanced antigen processing in TPST2 knock-down tumors

**Fig. 5:**
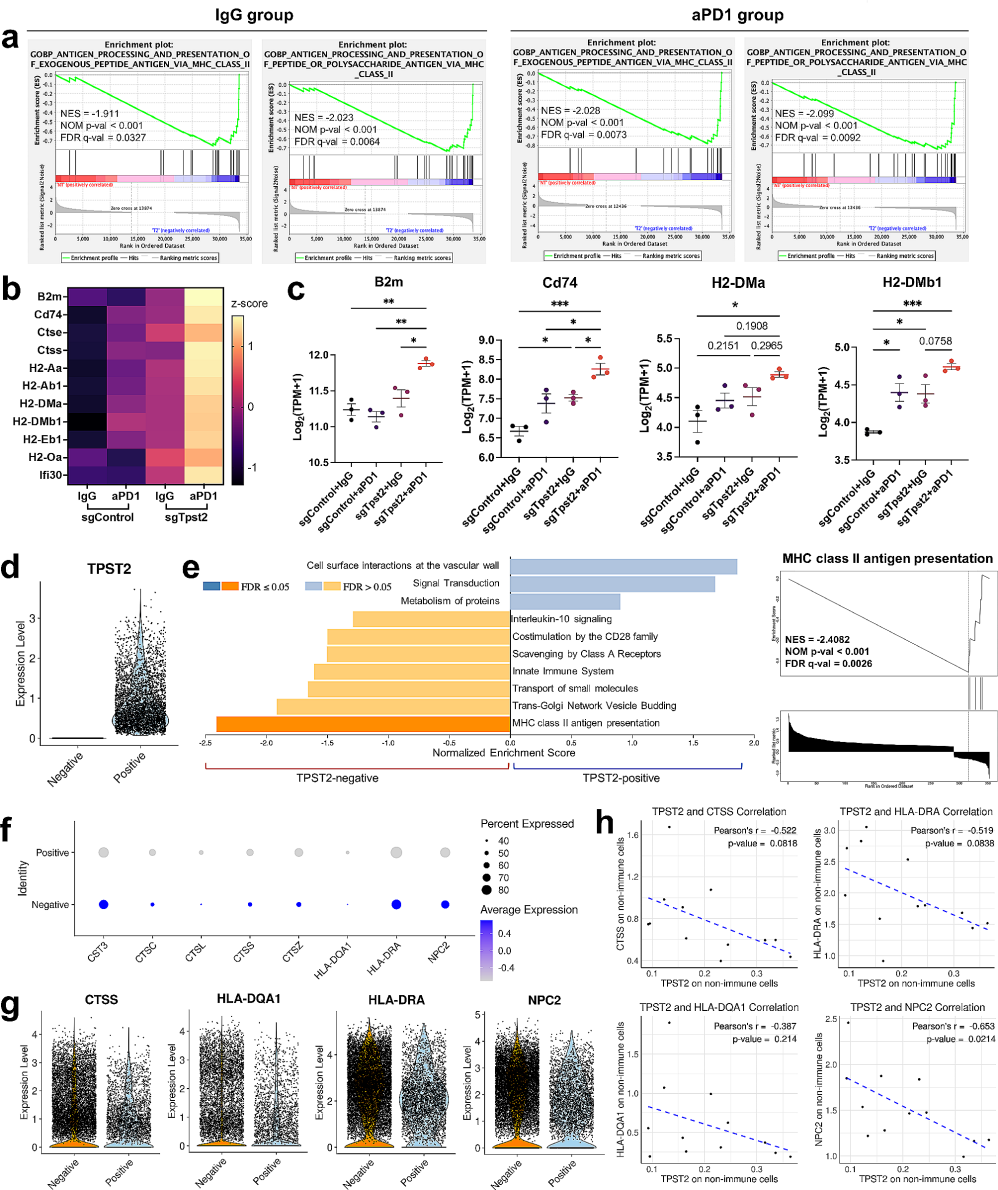
Tumor RNA sequencing in a syngeneic mouse model and single-cell RNA sequencing of human tumor tissues. **a**, Gene Set Enrichment Analysis (GSEA) of Tpst2 knock-down tumors from a syngeneic mouse model. Utilizing RNA sequencing data from mouse tumor tissues, this analysis compares the genetic profiles of Tpst2 knock-down tumors to control tumors. Tumor samples were collected from the syngeneic mouse model depicted in Fig. 4b. The comparison was made between the control + IgG group and the Tpst2 knock-down + IgG group, as well as the control + anti-PD1 group and the Tpst2 knock-down + anti-PD1 group, to highlight the distinctions between control and Tpst2 knock-down effects; *n* = 3 per group. **b**, Heatmap displays the expression levels of 11 genes related to the antigen processing and presentation process, as commonly identified in the GSEA results from (**a**). Each gene’s expression level is normalized to z-scores for comparative visualization. **c**, Dot plots showing key genes in antigen processing and presentation. Data are presented as mean ± SEM, with significance determined through one-way ANOVA. Asterisks indicate significant differences between the samples (*: *P* < 0.05; **: *P* < 0.01; ***: *P* < 0.001). **d**, Single-cell violin plot for comparing TPST2 expression between TPST2-positive and TPST2-negative non-immune cells. Conducting single-cell RNA sequencing analysis using public lung adenocarcinoma datasets (12 patients), we distinguished 74,888 cells into immune and non-immune categories based on the annotation results, and further focused on non-immune cells, identifying TPST2-positive and TPST2-negative populations. **e**, GSEA of TPST2-associated genomic alterations. GSEA, conducted with the WebGestalt tool, identifies significant pathways among 63 upregulated and 297 downregulated genes in TPST2-negative non-immune cells compared to TPST2-positive non-immune cells (left panel). A specific GSEA plot highlights the enrichment of MHC class II antigen presentation pathway in TPST2-negative cells (right panel). **f**, Comparative gene expression related to MHC class II antigen presentation. This analysis compares the expression of genes, as identified in the right panel of (**e**), between TPST2-positive and TPST2-negative non-immune cells. Dot size represents the percentage of cells expressing each gene, while dot color indicates the average expression levels. **g**, Single-cell violin plots for MHC class II antigen presentation-related genes. Violin plots compare the expression of genes, as identified in the right panel of (**e**), between TPST2-positive and TPST2-negative non-immune cells. Each dot within the plots represents an individual cell. **h**, Correlation analysis between TPST2 expression and MHC class II antigen presentation-related genes. Plots depict the correlation between TPST2 levels and the expression of representative genes in non-immune cells, with each dot representing the average expression per patient. Significance is determined using Pearson’s correlation test

To further elucidate the mechanistic basis of TPST2 knock-down on enhancing anti-tumor immunity and the efficacy of anti-PD1 therapy, we performed RNA sequencing on residual tumor tissues from syngeneic mouse model (Fig. 4b). GSEA directly compared control tumors with Tpst2 knock-down tumors in either IgG or anti-PD1-treated groups. Using the Gene Ontology Biological Processes (GO_BP) as a reference, we discovered enrichment of antigen processing and presentation pathways in Tpst2 knock-down tumors under both IgG and anti-PD1 treatments (FDR Q-value < 0.05; Fig. 5a, Fig. S15a, b), underscoring Tpst2’s critical role in modulating immune recognition mechanisms. An in-depth examination of 11 central genes in antigen processing and presentation process revealed a significant upregulation in Tpst2 knock-down tumors compared to controls (Fig. 5b, c, Fig. S15c). This upregulation was also significantly pronounced in the anti-PD1 treated groups versus IgG controls (Fig. 5b, c, Fig. S15c), highlighting a synergistic effect of Tpst2 inhibition and anti-PD1 therapy on these immune-related genes. Differential gene expression analysis highlighted 83 upregulated and 68 downregulated genes in Tpst2 knock-down tumors post anti-PD1 treatment (P_adj_ < 0.05) (Fig. S16a). A subsequent STRING analysis grouped the upregulated genes into two main clusters: one related to electron transport and the other to immune interactions, with the latter emphasizing antigen processing and presentation (Fig. S16b).

To extend our findings to a cellular resolution, we employed single-cell RNA sequencing data from public lung cancer and glioma cohorts. For the lung cancer cohort, we initially annotated all clusters based on the most distinct markers for each cluster (Fig. S16c, d) and confirmed that TPST2 is ubiquitously expressed across multiple cell populations (Fig. S16e). Based on the annotation results, we divided all clusters into immune cells and non-immune cells. Among non-immune cells, we then categorized these cells into TPST2-positive (3,717 cells) and TPST2-negative (14,391 cells) populations (Fig. 5d). We identified 360 genes exhibiting significant differential expression and found that MHC class II antigen presentation pathways were significantly more prominent in TPST2-negative cells (Fig. 5e). Examination of the expression levels of eight genes involved in this process confirmed significantly higher expression in TPST2-negative cells (Fig. 5f, g). Further analysis on an individual patient level also highlighted a negative correlation between TPST2 levels and the expression of these pivotal genes (Fig. 5h). Further investigating TPST2-negative non-immune cells, we divided them into tumor and non-tumor cells using gene scoring with representative tumor markers (Fig. S16f). More than 95% of tumor cells consisted of epithelial cells, while non-tumor cells included epithelial cells, endothelial cells, fibroblasts, and proliferating cells (Fig. S16g). Among the TPST2 negative non-immune cells, 6,743 (46.86%) were tumor cells, and 7,648 (53.14%) were non-tumor cells (Fig. S16g). Through cell-cell communication analysis, we examined the interactions between TPST2-negative tumor and non-tumor cells. The interactions where non-tumor cells influenced tumor cells included NAMPT → INSR, MIF → CD74 and CXCR4, GRN → SORT1, and CD99 → CD99 (Fig. S16h). These interactions illustrate how non-tumor cells in the tumor microenvironment can significantly influence tumor behavior and progression, highlighting potential therapeutic targets. Interestingly, the interaction with the highest probability was observed in the tumor → non-tumor direction for the APP → CD74 interaction (Fig. S16h). APP is involved in various cellular processes, including cell adhesion, neurite outgrowth, and synapse formation [33], while CD74 acts as a chaperone for MHC class II molecules and is involved in antigen presentation [34]. This interaction suggests that TPST2-negative tumor cells play a role in immune modulation, potentially enhancing the immune response within the tumor microenvironment. Similarly, in our analysis of the glioma cohort, we observed that antigen processing and presentation were significantly enriched in TPST2-negative non-immune cells compared to TPST2-positive cells (Fig. S17a-h). Since IFN-γ significantly boosts the expression of genes critical for antigen processing and presentation, these in vivo results directly align with the in vitro increase in IFN-γ signaling, indicating that TPST2 knock-down’s regulation of antigen processing and presentation significantly boosts immune recognition and the tumor immune response.

### Increased TPST2 expression is associated with prognosis and tumor immunity in cancer patients

**Fig. 6:**
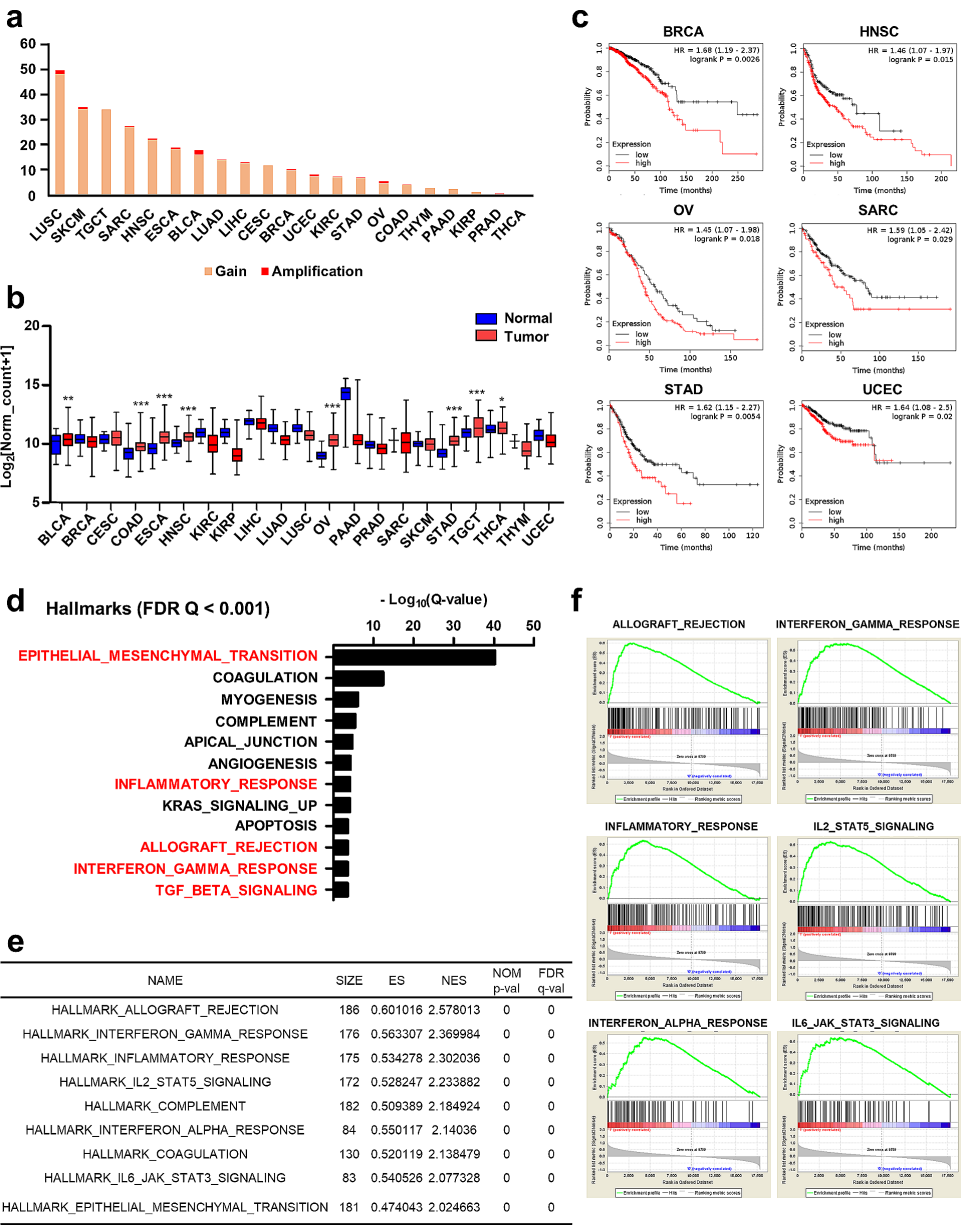
Effect of TPST2 expression on cancer immunity and prognosis in transcriptomic analysis of cancer patient samples. **a**, The proportion of patients with copy number alterations of TPST2 gene in tumor samples across the cancer types. Data were extracted from putative GISTIC copy number variation (CNV) of The Cancer Genome Atlas (TCGA) PanCancer Atlas studies in cBioPortal (https://www.cbioportal.org/). **b**, The mRNA expressions of TPST2 in normal and cancer tissues across the cancer types. Data were extracted from normalized gene expression (Log_2_[normalized count + 1]) of TCGA PanCancer Atlas studies in USCS Xena (https://xena.ucsc.edu/). Asterisks indicate significant differences between normal and cancer tissues (*: *P* < 0.05; **: *p* < 0.01; ***: *p* < 0.001). **c**, Survival analysis according to TPST2 expression across the cancer types. Kaplan-Meier plots for overall survival of patients with high and low TPST2 mRNA expressions were demonstrated. Red and black lines represent samples with high and low TPST2 expressions, respectively. Each hazard ratio (HR) of high TPST2 expression and P-value, determined by log rank test, is shown. **d**, Hallmark gene set analysis of genes with positive expression correlation with TPST2 in breast cancer. Hallmark gene set analysis was performed using 482 genes, of which expressions were positively correlated with TPST2 expression (Spearman’s correlation coefficient ρ ≥ 0.3), and enriched gene sets are demonstrated (Q < 0.001). **e**, Gene set enrichment analysis (GSEA) for breast cancer tissue microarray data according to the TPST2 expression. Microarray data of 238 triple-negative breast cancer patients (GSE103091) were downloaded from the Gene Expression Omnibus (GEO) database (https://www.ncbi.nlm.nih.gov/geo). GSEA was performed between 20 samples with highest expression levels of TPST2 (TPST2_H) and 20 samples with lowest expression levels of TPST2 (TPST2_L). The significantly enriched gene sets in TPST2_H (Q < 0.001) were listed. ES: enrichment score, NES: normalized enrichment score, NOM p-val: nominal P-value, FDR q-val: false discovery rate Q-value. **f**, Enrichment plots of representative gene sets that were significantly enriched in TPST2_H group. On the x-axis, genes are ranked from the most upregulated to the most downregulated between TPST2_H (left end) and TPST2_L (right end) groups. The y-axis shows a running enrichment score for TPST2 expression

To understand the clinical significance of TPST2 in cancer patients, we analyzed the genomic alterations and expressions of TPST2 using dataset from The Cancer Genome Atlas (TCGA) PanCancer Atlas studies [35] (http://www.cbioportal.org). The copy number gains or amplifications of TPST2 gene were observed in several types of cancers (Fig. 6a), and the mRNA expressions of TPST2 significantly increased compared to normal tissues in several types of tumor tissues (*P* < 0.05, Fig, 6b). In addition, a subset of patients in each tumor type exhibited up-regulation of TPST2 (z-score threshold of 2 from cBioPortal database (http://www.cbioportal.org; Fig. S18a). Patients with high expression of TPST2 demonstrated poorer prognosis compared to patients with low expression of TPST2 in breast invasive carcinoma (BRCA), head and neck squamous cell carcinoma (HNSC), ovarian serous cystadenocarcinoma (OV), sarcoma (SARC), stomach adenocarcinoma (STAD), and uterine corpus endometrial carcinoma (UCEC) (Fig. 6c).

Next, we investigated the roles of TPST2 in breast cancer patients by analyzing the transcriptome data. From the cohort of TCGA breast invasive carcinoma (PanCancer Atlas, *n* = 1084), we selected 482 genes, of which expressions were positively correlated with TPST2 expression (Spearman’s correlation coefficient ρ ≥ 0.3), and 323 genes, of which expressions were negatively correlated with TPST2 expression (Spearman’s correlation coefficient ρ ≤ -0.3) (Supplementary Table S3, S4). The positively correlated genes with TPST2 were enriched in several immune-related hallmarks gene sets, including ‘EPITHELIAL_MESENCHYMAL_TRANSITION’, ‘INFLAMMATORY_RESPONSE’, ‘ALLOGRAFT_REJECTION’, ‘INTERFERON_GAMMA_RESPONSE’, and ‘TGF_BETA_SIGNALING’ (FDR Q < 0.001; Fig. 6d). The negatively correlated genes were enriched in several cell cycle-related hallmarks gene sets, including ‘G2M_CHECKPOINT’, ‘E2F_TARGETS’, and ‘MITOTIC_SPINDLE’ (FDR Q < 0.001; Fig. S18b). We also investigated another data set of breast cancer cohort from the Gene Expression Omnibus (GEO) database (https://www.ncbi.nlm.nih.gov/geo), which consists of microarray data from 238 triple-negative breast cancer patients (GSE103091). We selected 20 samples with highest expression levels of TPST2 (TPST2_H) and 20 samples with lowest expression levels of TPST2 (TPST2_L), and performed GSEA between these two groups. When gene sets from gene ontology (GO) biological process were applied, gene sets associated with cancer immunity such as ‘ALLOGRAFT_REJECTION’, ‘INTERFERON_GAMMA_RESPONSE’, ‘INFLAMMATORY_RESPONSE’, ‘IL2_STAT5_SIGNALING’, ‘INTERFERON_ALPHA_RESPONSE’, and ‘IL6_JAK_STAT3_SIGNALING’ were highly enriched in TPST2_H group (Fig. 6e, f). In TPST2_L group, several gene sets associated with cell cycle regulation including ‘E2F_TARGETS’, ‘G2M_CHECKPOINT’, and ‘MITOTIC_SPINDLE’ were enriched (Fig. S18c, d). While these findings may seem contradictory to previous experimental results, they suggest that TPST2 is associated with tumor immunity including IFNγ signaling, and cell cycle regulation in breast cancer tissues.

## Discussion

To overcome the insufficient success of ICT in cancer patients, several regimens for ICT-based combination treatments are being attempted [3], and one of the molecular mechanisms of these combination therapies is the induction of IFNγ signaling. Combination of anti-CTLA4 and anti-PD1 antibodies were effective in melanoma, renal cell carcinoma, and microsatellite instability-high (MSI-H) colorectal cancers [36], and concomitant blockade of CTLA4 and PD1 increased production of IFNγ from CD8^+^ cells [37]. Targeting other immune inhibitory molecules such as TIM3 and LAG3 demonstrated synergistic effect with anti-PD1 antibodies and increased the proportion of IFNγ-producing CD8^+^ cells [38, 39]. Several targeted agents including a receptor tyrosine kinase inhibitor, lenvatinib and a PARP inhibitor, niraparib potentiated the efficacy of ICT via activation of IFNγ signaling [40, 41]. However, tumor-specific T cells highly expressing IFNγ receptor were more susceptible to apoptosis and clonal deletion of these cells confer resistance to ICT [42]. Therefore, optimal activation of IFNγ signaling is one of key determinants for successful combination immunotherapy.

Human IFNGR1 is a single pass membrane receptor with 489 amino acids and expressed in both cancer and immune cells [43]. The activity and stability of IFNGR1 protein is largely regulated via several kinds of post-translational modifications. Complex glycosylation of IFNGR1 occurs in Golgi apparatus during protein targeting, and maturely glycosylated IFNGR1 participates in IFNγ signaling on plasma membrane [44]. Phosphorylation of IFNGR1 at Y457 by JAK1 and JAK2 provide docking sites for STAT1, which is required for proper IFNγ signaling [45]. In addition, phosphorylation of IFNGR1 by glycogen synthase kinase 3 beta (GSK3β) inhibited the ubiquitination of IFNGR1 and increased the protein stability [43]. Regulation of IFNGR1 plays a critical role in IFN-γ responsiveness, as our data demonstrated that overexpression of IFNGR1 increased IFN-γ-mediated growth inhibition (Figs. 2e and 3h). Additionally, the proliferation of MDA-MB-468 cells was significantly more suppressed by IFN-γ treatment compared to MDA-MB-231 cells in wild-type conditions (Fig. 2e), probably due to increased expression of genes involved in IFN-γ receptor-mediated pathways, including IFNGR1, STAT1, and IRF1 (Fig. S19). Our study is a first report for the modulation of IFNGR1 activity by tyrosine sulfation. Tyrosine sulfation of IFNGR1 was validated by anti-sulfotyrosine antibody (Fig. 3b) and mass spectrometry (Fig. S10a, b). Because tyrosine sulfation has been suggested to modulate the extracellular protein-protein interactions [8], one possible molecular mechanism is that the TPST2-mediated tyrosine sulfation of IFNGR1 suppresses IFNγ signaling by altering the interaction between IFNγ and the IFNγ receptor. The more detailed structural analyses and molecular mechanisms of IFNGR1 tyrosine sulfation in IFNγ signaling need to be further investigated.

Our data demonstrated that TPST2 knockdown enhanced IFNγ signaling in human breast cancer cell lines (MDA-MB-231 (Fig. 2 and Fig. S5) and MDA-MB-468 (Fig. S6)) and mouse breast (4T1 (Fig. S11d, e) and colon (MC38 (Fig. S11b, c)) cancer cell lines. Since MDA-MB-231, MDA-MB-468, and 4T1 cells are models for triple-negative breast cancer (TNBC), these findings strongly indicate that TPST2 regulates IFNγ signaling, at least in TNBC. Single-cell RNA sequencing data from public lung cancer and glioma cohorts demonstrated that the expression of TPST2 was negatively correlated with genes associated with antigen presentation (Fig. 5d-h, Fig. S16, S17), which is one of the representative down-stream targets for IFNγ signaling. Furthermore, we observed copy number gains or amplifications of the TPST2 gene (Fig. 6a) and significantly increased TPST2 mRNA expression (Fig. 6b) in various cancers, with higher TPST2 expression correlating with poorer prognosis in several cancer types, including BRCA, HNSC, OV, SARC, STAD, and UCEC (Fig. 6c). These findings suggest that TPST2 regulates cancer immunity in multiple cancer types.

TPST2 mediates tyrosine O-sulfation of membrane and secretory proteins in trans-Golgi network, and has been suggested to regulate the extracellular protein-protein interactions [8]. Tyrosine sulfation of glycoprotein hormone receptors such as thyrotrophin receptor (TSHR), luteinizing hormone/chorionic gonadotrophin receptor (LH/CGR), and follicle-stimulating hormone receptor (FSHR) is required for high-affinity binding of ligands to receptors [46]. Several chemokine receptors including CCR5, CCR2b, CCR8, CXCR3, CXCR4, and CX3CR1 were reported to be modified by tyrosine sulfation in N-terminal domains, which is required for chemokine binding [47]. Our data suggested a novel role of TPST2 in the regulation of cancer immunity, in which TPST2-mediated tyrosine sulfation of IFNGR1 at Y397 constrained IFNγ signaling (Fig. 3e-h). TPST2-mediated tyrosine sulfation of IFNGR1 may affect its binding affinity to IFNγ or its dimerization with IFNGR1 or IFNGR2 within the IFNγ receptor complex, but the precise molecular effects of this modification require further investigation. Interestingly, TPST1 and TPST2, which share about 64% amino acid similarity [20], demonstrated different effects on IFNγ signaling (Fig. 2, Fig. S7). These results were consistent with a previous report demonstrating that substrate specificity and tissue distribution was different between TPST1 and TPST2 [19]. In addition, knock-down mice of TPST1 and TPST2 showed a distinct phenotype for each gene, suggesting little functional redundancy between these two genes [6, 48]. The detailed molecular mechanisms by which TPST1 regulates IFNγ signaling need to be further studied.

In breast cancer tissue samples from TCGA, the expression of TPST2 is significantly associated with increased expressions of IFNγ signaling-related genes (Fig. 6e, f). However, these findings are inconsistent with our cell line data suggesting that TPST2 inhibits the IFNγ signaling pathway. One plausible explanation is that the elevated expression of TPST2 serves as a negative feedback mechanism for cancer immunosuppression in tumors exhibiting heightened IFNγ. Therefore, targeting TPST2 represents a promising approach for combination therapy with anti-PD1. In addition to roles in the regulation of IFNγ signaling, TPST2 probably plays additional roles in cancer cells. Copy numbers and gene expression levels of TPST2 significantly increased in several cancer types (Fig. 6a, b). Breast cancer tissues with high TPST2 expression demonstrated the enriched expression of cancer aggressiveness-related gene sets such as ‘EPITHELIAL_MESENCHYMAL_TRANSITION’ (Fig. 6d-f) and depleted expression of cell cycle checkpoint-related gene sets such as ‘E2F_TARGETS’, ‘G2M_CHECKPOINT’, and ‘MITOTIC_SPINDLE’ (Fig. S18b-d). These alterations of gene expression by TPST2 highly associated with poor prognosis in certain types of cancers (Fig. 6C). These data suggest TPST2 as an attractive target for cancer therapeutics, governing both intrinsic characteristics of cancer cells and cancer immunity.

### Electronic supplementary material

Below is the link to the electronic supplementary material.

Supplementary Material 1

Supplementary Material 2

Supplementary Material 3

## Data Availability

The RNA sequencing data have been deposited in the European Nucleotide Archive (accession no. PRJEB73786).
