# Supplementary material for: Genome-wide CRISPR screening identifies tyrosylprotein sulfotransferase-2 as a target for augmenting anti-PD1 efficacy
Source: Mol Cancer. 2024 Aug 2;23:155. doi: 10.1186/s12943-024-02068-x (PMC11295332; doi:10.1186/s12943-024-02068-x)
Supplement: Supplementary file 1 — Supplementary Material 1 [file 12943_2024_2068_MOESM1_ESM.pdf]

Supplementary Materials and Methods

Lentiviral genome-scale CRISPR knock-out (GeCKO) library production

For lentivirus generation, 293FT cells (2.5×10^6) were co-transfected with 6 μg of GeCKOv2 library, 3 μg of pCMV-VSV-G (a gift from Bob Weinberg; Addgene plasmid #8454), and 4.5 ug of psPAX2 (a gift from Didier Trono; Addgene plasmid #12260). Plasmid transfection was performed using Lipofectamine 2000 reagent (Invitrogen) according to the manufacturer's instructions. Next day, the culture medium was changed, and cells were grown for additional 24 h. Culture media from transfected 293FT cells were harvested and clarified through centrifugation at 500 g for 10 min. The virus was concentrated using Lenti-X concentrator (Clontech) according to the manufacturer's instructions.

Transcriptomic analysis using RNA sequencing

In RNA sequencing for cell lines, total RNA from cells after 1 ng/ml IFNγ treatment for 8 h was extracted using RNeasy Plus Mini Kit (Qiagen). Whole-transcriptome expression profiles were generated by RNA sequencing using Mapsplice and RSEM with TCGA RNASeq v2 pipeline (https://wiki.nci.nih.gov/display/TCGA/RNASeq+Version+2) [1, 2]. Differentially expressed genes were estimated utilizing the edgeR packages [3]. To perform mouse tumor RNA sequencing analysis, we used the kallisto software for pseudoalignment and quantification of transcript abundances. The process was divided into two main steps: index creation and quantification. Firstly, we constructed a kallisto index for the Mus musculus genome (GRCm39) to facilitate efficient mapping of RNA-seq reads. This was achieved by downloading the primary assembly fasta file from the Ensembl database (release 108). The kallisto index was then built with a k-mer size of 31. We quantified each sample's paired-end reads against the crafted index, employing a bootstrapping value of 100 to ensure robust

statistical analysis. The RNA sequencing data have been deposited in the European Nucleotide Archive (accession no. PRJEB73786).

Mass spectrometry analysis for tyrosine sulfation of protein

For mass spectrometric analysis, immunoprecipitated myc-tagged IFNGR1 sample was prepared as described previously with some modifications [4]. Briefly, elution buffer (2% SDS, 5 mM tris(2-carboxyethyl)phosphine (TCEP), 20 mM chloroacetamide (CAA) in 50 mM ammonium bicarbonate (ABC)) was added to the washed beads. The mixture was boiled for 15 min at 95°C to elute the immunoprecipitated proteins. Eluted proteins were digested by filter aided sample preparation (FASP) procedure as previously described [5]. Briefly, the eluate was loaded onto a 30K amicon filter (Millipore, Burlington, MA, USA). Buffer exchanges were performed with the UA solution (8 M urea in 0.1 M Tris pH 8.5) via centrifugation at 14,000 × g for 15 min. Following an exchange of the buffer with 40 mM ammonium bicarbonate, protein digestion was performed at 37°C overnight using a trypsin/LysC mixture (Promega, Madison, WI, USA) at a 100:1 protein-to-protease ratio. The digestion-generated peptides were collected by centrifugation. After the filter units were washed with 40 mM ammonium bicarbonate, a second digestion was performed at 37°C for 2 h with trypsin (enzyme-to-substrate ratio [w/w] of 1:1000). All resulting peptides were acidified with 10% of TFA and desalted using homemade C18-SDB-RPS-StageTip column as previously described [5]. Desalted samples were completely dried in a vacuum dryer and stored at -80 °C.

LC-MS/MS analysis was performed using a Q Exactive Plus mass spectrometer (Thermo Fisher Scientific, Waltham, MA, USA), coupled to Ultimate 3000 RSLC system (Dionex, Sunnyvale, CA, USA) via an Easy-Spray source, as previously described [6]. Prior to sample injection, the dried peptide samples were re-dissolved in solvent A (2% [v/v] acetonitrile and 0.1% [v/v] formic acid). Peptide samples were separated on a two-column system, consisting

of a trap column (300 µm ID x 5mm, C18, 5µm) and an analytic column (75 µm ID x 50 cm, C18, 1.9 µm, 100 Å) with a 120 min gradient from 6% to 30% acetonitrile at 300 nl/min and anlyzed by mass spectrometry. Column temperature was maintained at 60°C using an Easy-spray column heater. Survey scans (350 to 1650 m/z) were acquired with a resolution of 60,000 at m/z 200. A top-15 method was used to select precursor ions with an isolation window of 2 m/z. MS/MS spectra were acquired at an HCD with normalized collision energies of 28. The maximum ion injection time for the MS1 scan and the MS2 scan was 25 and 125 ms, respectively.

MS spectra were processed using Maxquant softwere version 1.6.1.0 [7]. MS/MS spectra were searched against the myc-tagged IFNGR1 sequence and the Uniprot Human protein sequence database (version 12.2014, 88,657 entries) including forward and reverse sequence and common contaminants. Primary searches were performed using a 6-ppm precursor ion tolerance for total protein level analysis. The MS/MS ion tolerance was set to 20 ppm. Cysteine (C) carbamidomethylation was set as a fixed modification. N-acetylation of protein, sulfation of tyrosine (Y), phosphorylation of serine (S), Threonine (T), and Tyrosine (Y), and oxidation of methionine (M) were set as variable modifications. Enzyme specificity was set to full tryptic digestion. Peptides with a minimum length of six amino-acids and up to two missed cleavages were considered as search parameters.

Analysis of data from The Cancer Genome Atlas (TCGA)

The profiles of genomic alterations and gene expressions of The Cancer Genome Atlas (TCGA) PanCancer Atlas studies [8] were downloaded from cBioPortal for cancer genomics (http://www.cbioportal.org). The survival analysis for each cancer type from TCGA PanCancer Atlas studies with respect to the expression of TPST2 were performed using the online bioinformatics tool Kaplan-Meier Plotter (https://kmplot.com/analysis/).

References for Supplementary Materials and Methods

1. Li B, et al. RNA-Seq gene expression estimation with read mapping uncertainty. Bioinformatics. 2010; 26(4):493-500.
2. Wang K, et al. MapSplice: Accurate mapping of RNA-seq reads for splice junction discovery. Nucleic Acids Research. 2010;38(18):e178.
3. Robinson MD, McCarthy DJ, Smyth GK. edgeR: a Bioconductor package for differential expression analysis of digital gene expression data. Bioinformatics. 2010;26(1):139-40.
4. Moon S, et al. Interactome analysis of AMP-activated protein kinase (AMPK)-alpha 1 and -beta 1 in INS-1 pancreatic beta-cells by affinity purification-mass spectrometry. Sci Rep. 2014;4:4376.
5. Han D, et al. Proteomic analysis of mouse astrocytes and their secretome by a combination of FASP and StageTip-based, high pH, reversed-phase fractionation. Proteomics. 2014;14(13-14):1604-9.
6. Park JH, et al. Moesin (MSN) as a novel proteome-based diagnostic marker for early detection of invasive bladder urothelial carcinoma in liquid-based cytology. Cancers (Basel) 2020;12(4):1018.
7. Tyanova S, Temu T, Cox J. The MaxQuant computational platform for mass spectrometry-based shotgun proteomics. Nat Protoc. 2016;11(12):2301-19.
8. Weinstein JN, et al. The Cancer Genome Atlas Pan-Cancer analysis project. Nature Genetics. 2013;45(10):1113-20.
