# Supplementary material for: Genome-wide CRISPR screening identifies tyrosylprotein sulfotransferase-2 as a target for augmenting anti-PD1 efficacy
Source: Mol Cancer. 2024 Aug 2;23:155. doi: 10.1186/s12943-024-02068-x (PMC11295332; doi:10.1186/s12943-024-02068-x)
Supplement: Supplementary file 2 — Supplementary Material 2 [file 12943_2024_2068_MOESM2_ESM.pdf]

Supplementary Figure S1

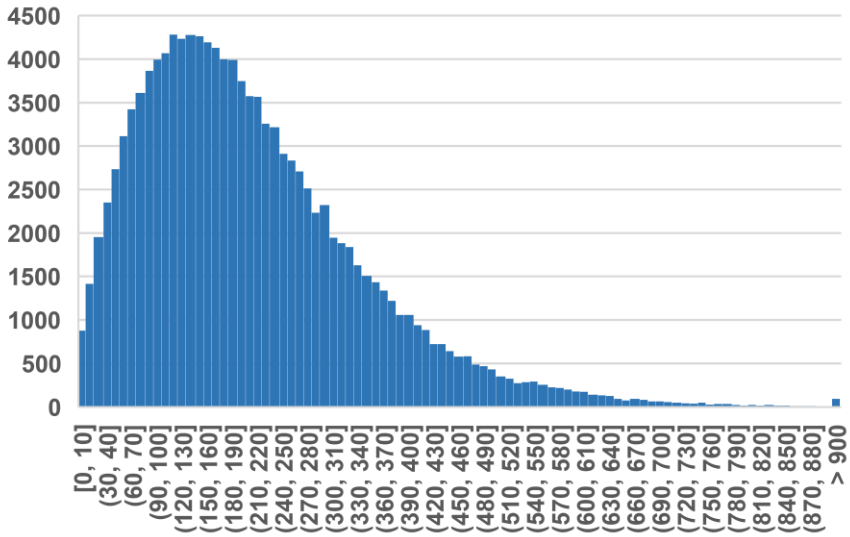

Supplementary Fig. S1. Distribution of sgRNA raw counts for the puromycin-resistant CRISPR library infected MDA-MB-231 cells. MDA-MB-231 cells were infected with the Human GeCKOv2 lentivirus library with MOI of 0.3–0.5 for 24 h and selected by puromycin treatment (1 μg/mL) for 3 days. The sgRNA distribution in the puromycin-resistant cells was analyzed by amplicon sequencing and demonstrated by histogram for sgRNA raw counts.

Supplementary Figure S2

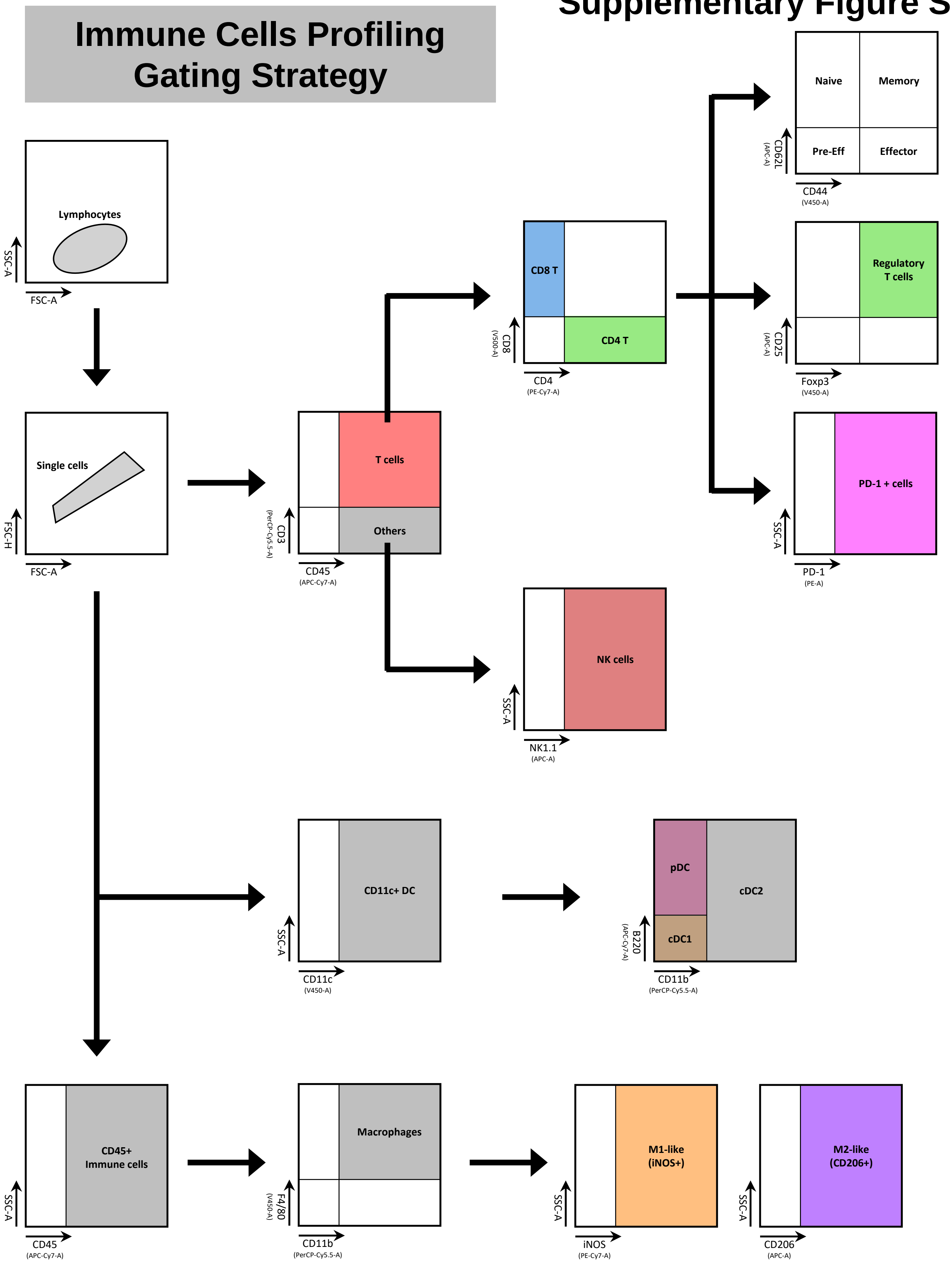

Supplementary Fig. S2. Gating strategy operated in flow cytometry analysis to identify various cell types .

Supplementary Figure S3

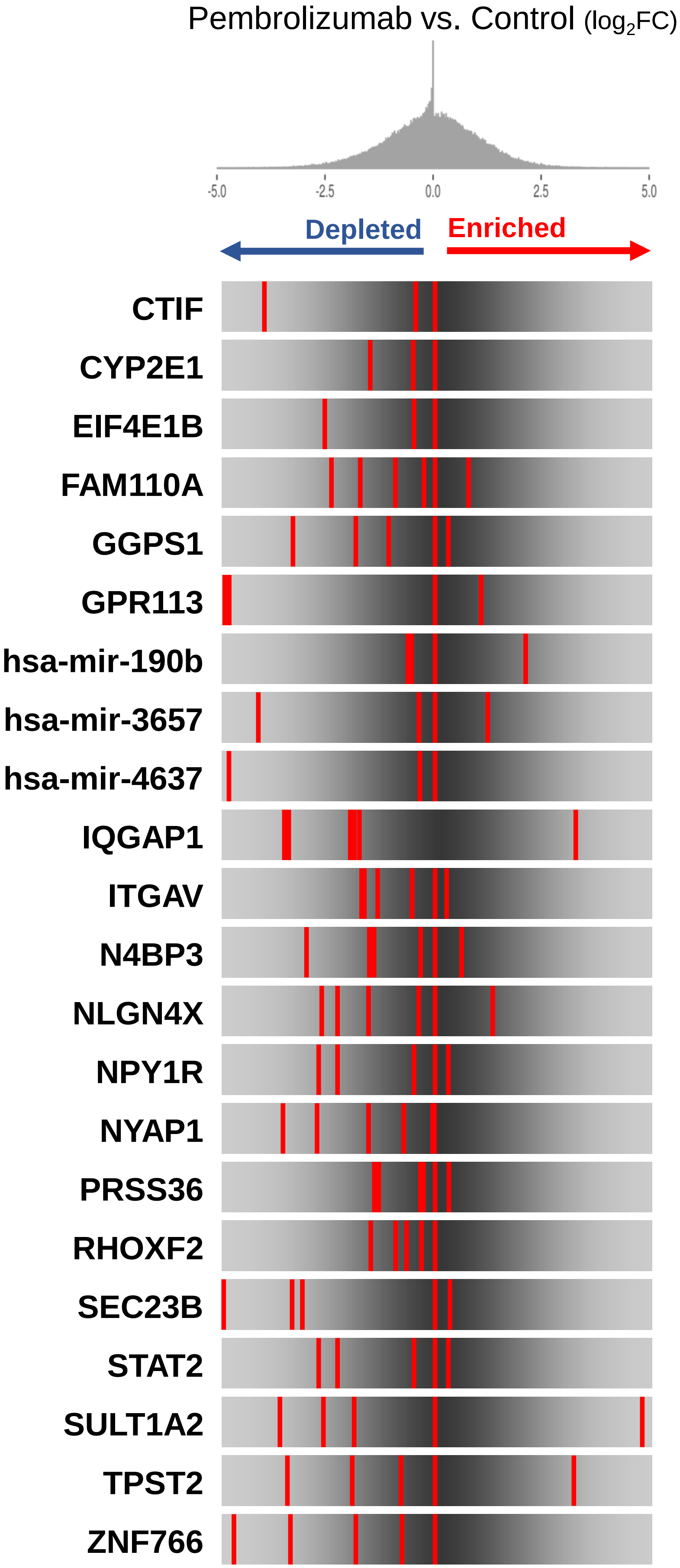

Supplementary Fig. S3. Rank distribution diagram of sgRNAs targeting 22 genes with multiple depleted sgRNAs in *in vivo* genome-wide CRISPR/Cas9 knockout screening for anti-PD1 responsiveness.

Supplementary Figure S4

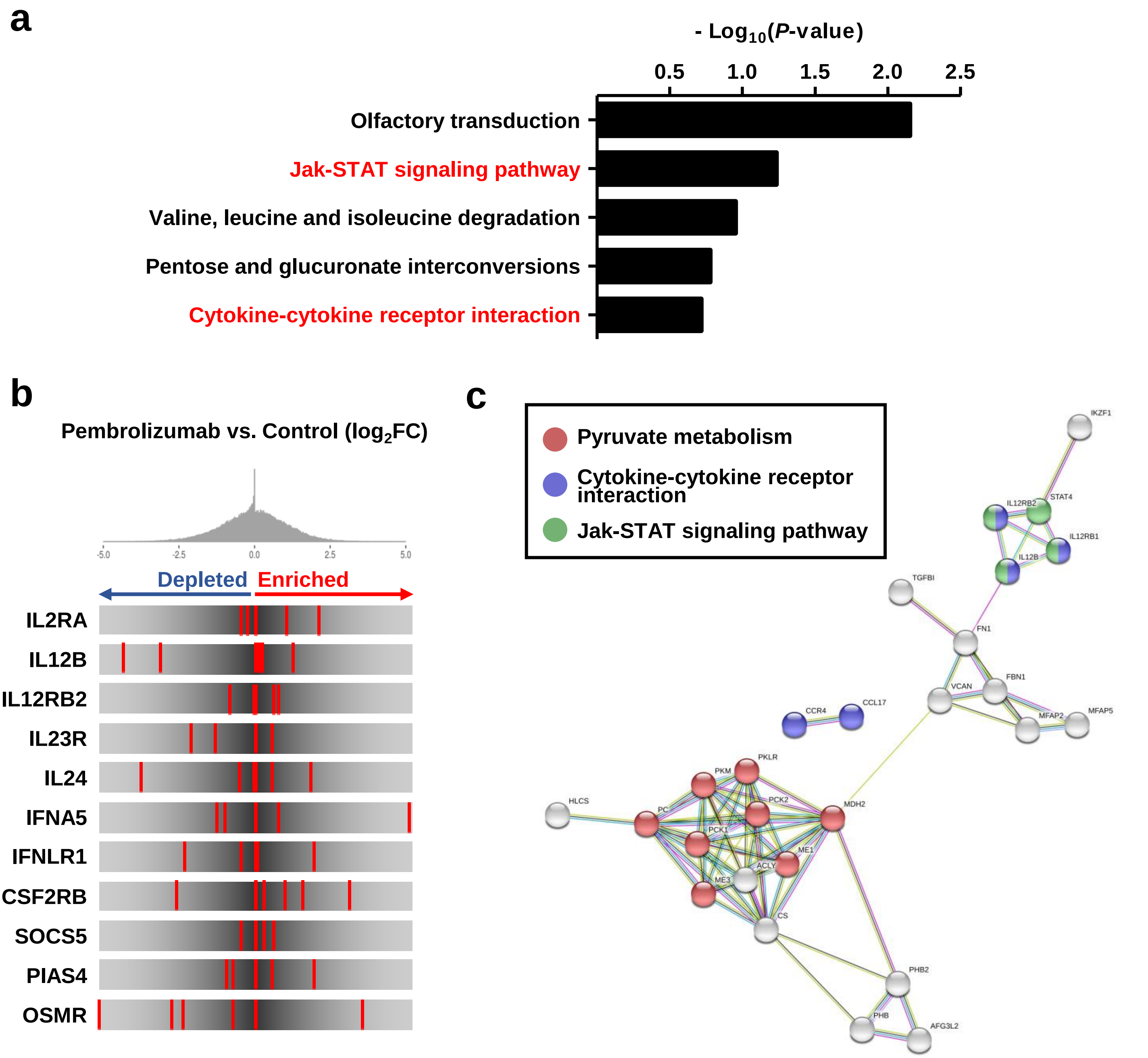

Supplementary Fig. S4. Pathway analyses of genes from *in vivo* genome-wide CRISPR/Cas9 knockout screening. a, KEGG pathway analysis of 777 genes, sgRNAs of which were enriched in anti-PD1-treated tumors. The KEGG pathways that were significantly enriched in the 777 genes ($P < 0.2$) are shown. **b**, Rank distribution diagram of sgRNAs targeting genes associated with Jak-STAT signaling pathway from 777 genes. **c**, STRING network analysis with genes with multiple enriched sgRNAs in anti-PD1-treated mice. Genes involved in each pathway are marked with the corresponding color.

Supplementary Figure S5

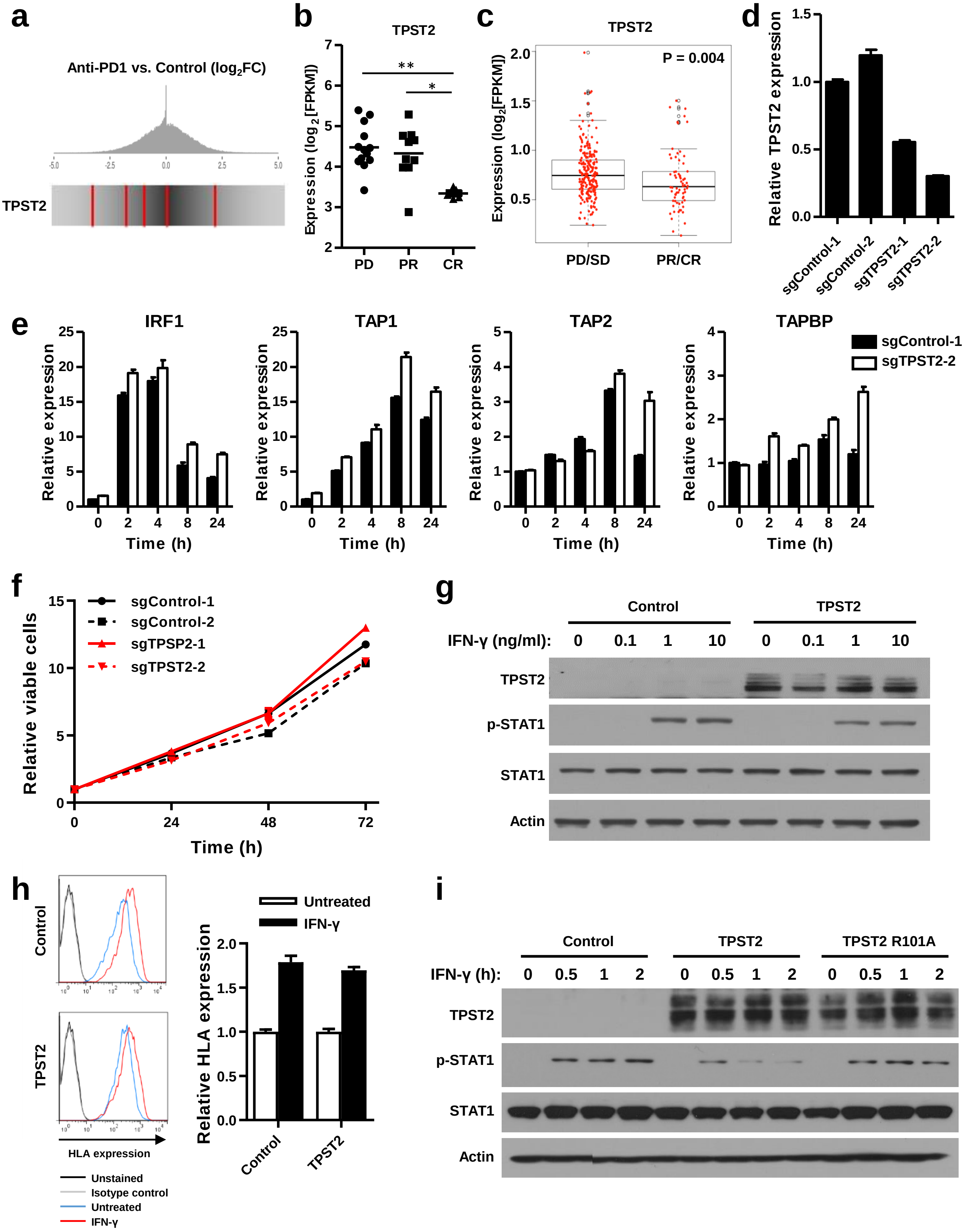

Supplementary Fig. S5. Effect of TPST2 on the IFNγ signaling pathway in breast cancer cells. a, Rank distribution diagram of sgRNAs targeting TPST2 in *in vivo* genome-wide CRISPR/Cas9 knockout screening for anti-PD1 responsiveness. **b**, TPST2 mRNA expressions in anti-PD1-treated melanoma patients according to treatment responsiveness. TPST2 expressions levels were estimated as fragments per kilobase of transcript per million (FPKM) values from RNA sequencing data of melanoma biopsies (GSE78220 from Gene expression omnibus (GEO)). PD: progressive disease, PR: partial response, CR: complete response. **c**, TPST2 mRNA expressions in anti-PD-L1-treated urothelial cancer patients according to treatment responsiveness. TPST2 expressions levels were estimated as FPKM values from RNA sequencing data of urothelial cancer patient tissues. PD: progressive disease, SD: stable disease, PR: partial response, CR: complete response. **d**, Reduced expression of TPST2 in TPST2 knock-down MDA-MB-231 cells. The expression levels of TPST2 were estimated by real-time PCR. **e**, Enhanced expression of IFNγ-responsive genes in TPST2-depleted breast cancer cells. TPST2 was knocked down using CRISPR/Cas9 and second single guide RNA in MDA-MB-231 cells. After serum starvation for 24 h, cells were treated with 10 ng/ml IFNγ and the expression levels of IFNγ-responsive genes were estimated by real-time PCR at indicated time points. **f**, Effect of TPST2 knock-down in cell proliferation. Cell numbers of control and TPST2 knock-down cells were estimated by trypan blue staining assay at indicated time points. **g**, Reduced phosphorylation of STAT1 in TPST2-overexpressed breast cancer cells. After serum starvation for 24 h, cells were treated with IFNγ at indicated concentration for 24 h. The phosphorylation levels of STAT1 were evaluated by western blotting. **h**, Reduced expression of human leukocyte antigen (HLA) by IFNγ treatment in TPST2-overexpressed breast cancer cells. After serum starvation for 24 h, cells were treated with 10 ng/ml IFNγ for 24 h and the expression levels of IFNγ-responsive HLA were estimated by flow cytometry. **i**, Effect of active site mutant of TPST2 in IFNγ-induced phosphorylation of STAT1. Wild type and active site mutant TPST2 were transfected to MDA-MB-231 cells for 24 h. After serum starvation for 24 h, cells were treated with IFNγ at indicated concentration for 24 h. The phosphorylation levels of STAT1 were evaluated by western blotting.

Supplementary Figure S6

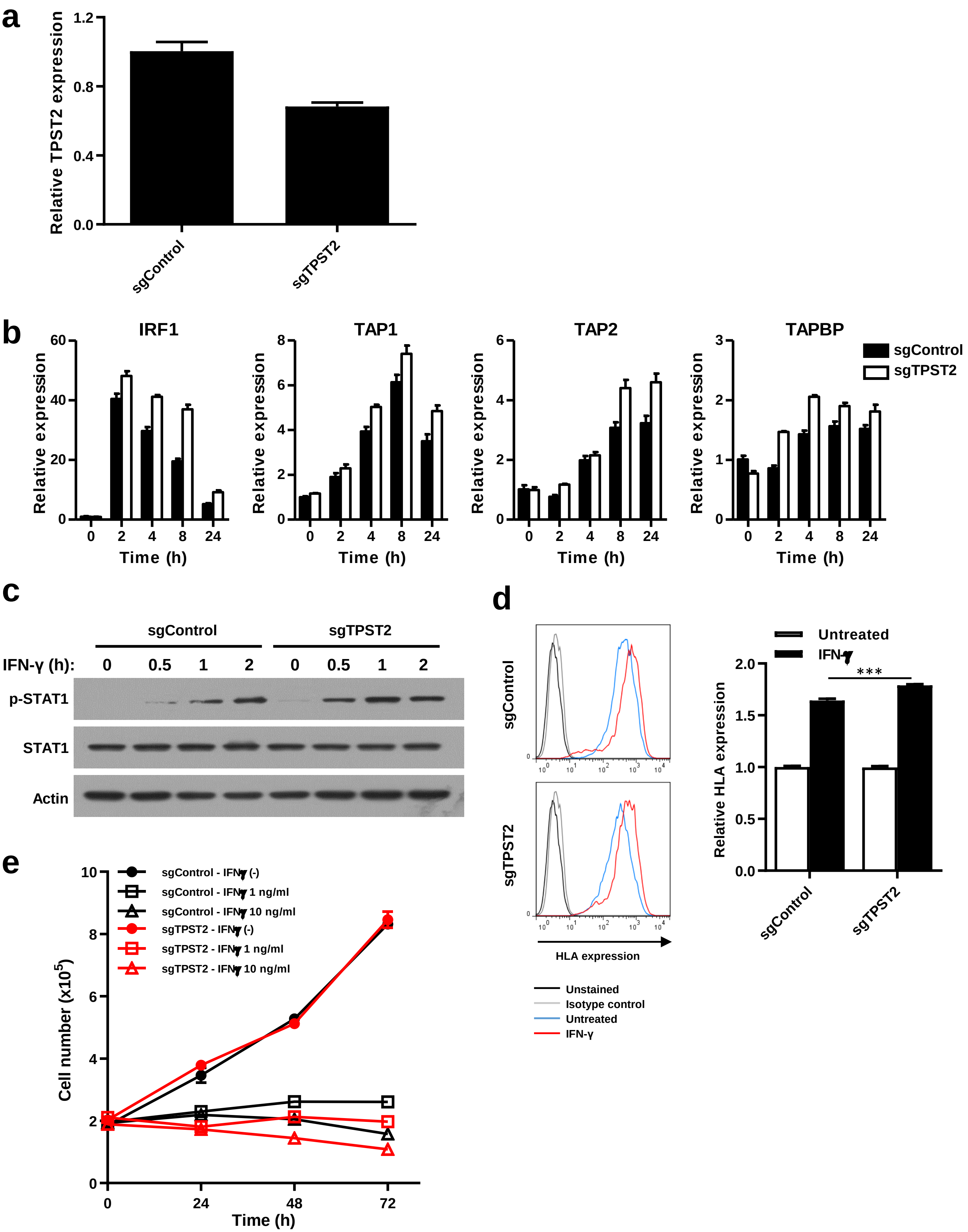

Supplementary Fig. S6. TPST2-mediated suppression of IFNγ signaling pathway in MDA-MB-468 breast cancer cells. **a**, Reduced expression of TPST2 in TPST2 knock-down MDA-MB-468 cells. The expression levels of TPST2 were estimated by real-time PCR. **b**, Enhanced expression of IFNγ-responsive genes in TPST2-depleted breast cancer cells. TPST2 was knocked down using CRISPR/Cas9 in MDA-MB-468 cells. After serum starvation for 24 h, cells were treated with 1 ng/ml IFNγ and the expression levels of IFNγ-responsive genes were estimated by real-time PCR at indicated time points. **c**, Enhanced phosphorylation of STAT1 in TPST2-depleted MDA-MB-468 cells. After serum starvation for 24 h, cells were treated with 0.1 ng/ml IFNγ for indicated time. The phosphorylation levels of STAT1 were evaluated by western blotting. **d**, Enhanced expression of human leukocyte antigen (HLA) by IFNγ treatment in TPST2-depleted MDA-MB-468 cells. After serum starvation for 24 h, cells were treated with 1 ng/ml IFNγ for 24 h and the expression levels of IFNγ-responsive HLA were estimated by flow cytometry. **e**, Enhanced suppression of cell proliferation by IFNγ treatment in TPST2-depleted MDA-MB-468 cells. After serum starvation for 24 h, cells were treated with 1 or 10 ng/ml IFNγ for indicated time points and the cell numbers were estimated by trypan blue staining assay.

Supplementary Figure S7

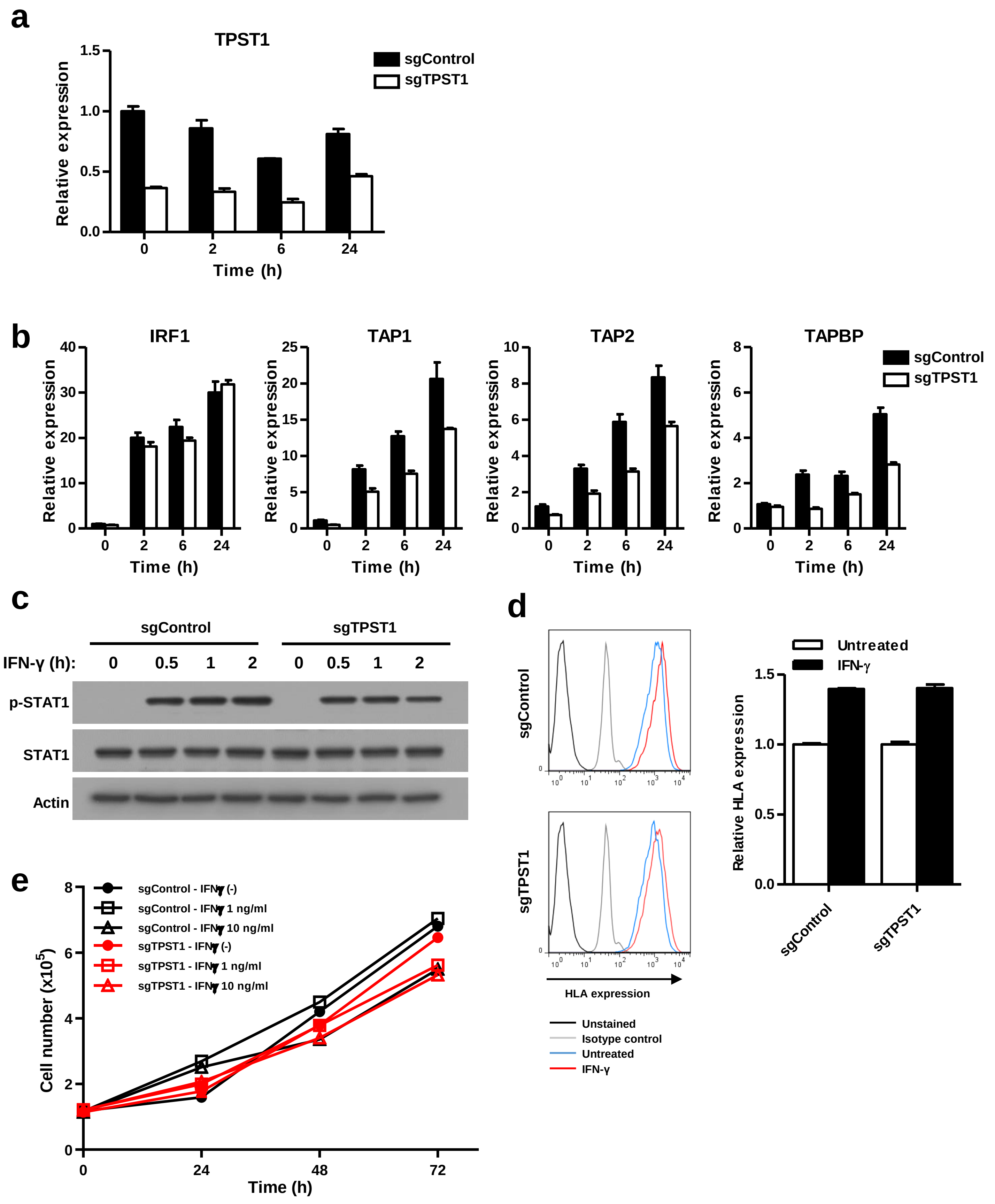

Supplementary Fig. S7. Effect of TPST1 knock-down on IFNγ signaling pathway in breast cancer cells. **a**, Reduced expression of TPST1 in TPST1 knock-down MDA-MB-231 cells. After treatment of IFNγ (10 ng/ml), the expression levels of TPST1 were estimated by real-time PCR. **b**, Expression of IFNγ-responsive genes in TPST1-depleted breast cancer cells. TPST1 was knocked down using CRISPR/Cas9 in MDA-MB-231 cells. After serum starvation for 24 h, cells were treated with 10 ng/ml IFNγ and the expression levels of IFNγ-responsive genes were estimated by real-time PCR at indicated time points. **c**, Phosphorylation of STAT1 in TPST1-depleted MDA-MB-231 cells. After serum starvation for 24 h, cells were treated with 1 ng/ml IFNγ for indicated time. The phosphorylation levels of STAT1 were evaluated by western blotting. **d**, Expression of human leukocyte antigen (HLA) by IFNγ treatment in TPST1-depleted MDA-MB-231 cells. After serum starvation for 24 h, cells were treated with 10 ng/ml IFNγ for 24 h and the expression levels of IFNγ-responsive HLA were estimated by flow cytometry. **e**, Suppression of cell proliferation by IFNγ treatment in TPST1-depleted MDA-MB-231 cells. After serum starvation for 24 h, cells were treated with 1 or 10 ng/ml IFNγ for indicated time points and the cell numbers were estimated by trypan blue staining assay.

Supplementary Figure S8

a

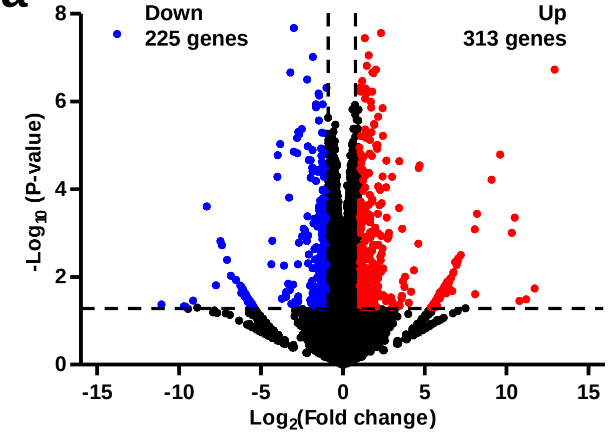

b

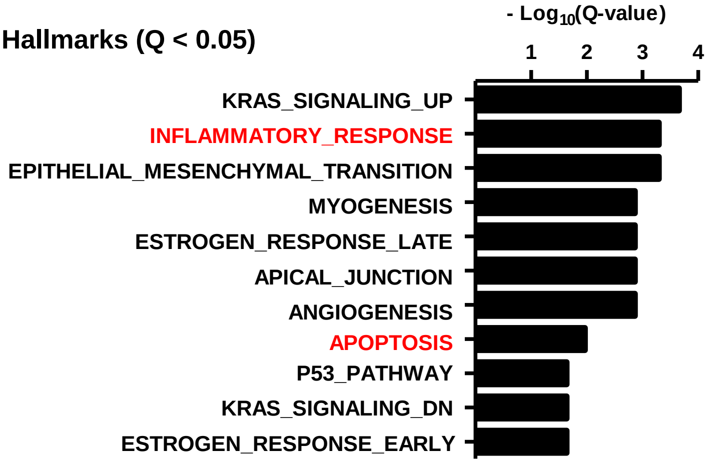

c

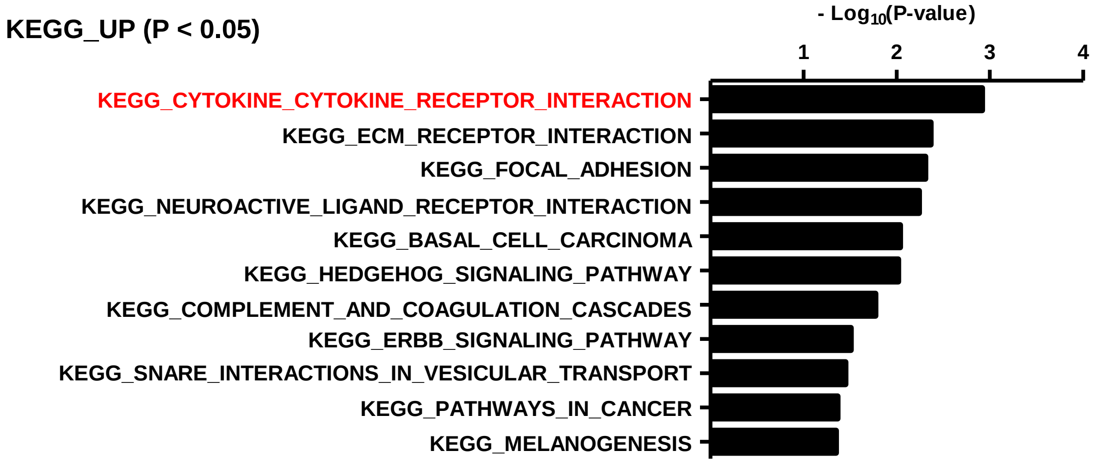

d

NAME	SIZE	ES	NES	NOM p-val	FDR q-val	FWER p-val
HALLMARK_TNFA_SIGNALING_VIA_NFKB	198	0.519986	1.608772	0	0.04322	0.041
HALLMARK_KRAS_SIGNALING_UP	195	0.483792	1.491591	0	0.090924	0.161
HALLMARK_INFLAMMATORY_RESPONSE	197	0.452219	1.400651	0.003289	0.116762	0.369
HALLMARK_HYPOXIA	194	0.434157	1.347449	0.009868	0.140468	0.563
HALLMARK_INTERFERON_GAMMA_RESPONSE	198	0.446564	1.381654	0.01248	0.117237	0.44
HALLMARK_APOPTOSIS	159	0.423734	1.292178	0.045902	0.209356	0.782
HALLMARK_ANGIOGENESIS	36	0.595047	1.409916	0.047101	0.141116	0.339

TNFA_SIGNALING_VIA_NFKB

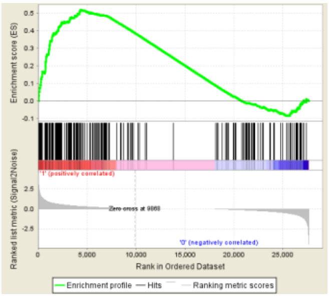

INFLAMMATORY_RESPONSE

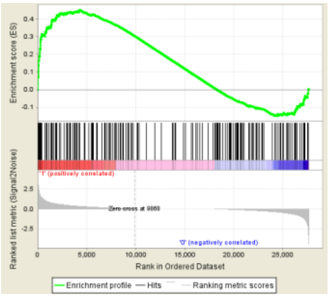

INTERFERON_GAMMA_RESPONSE

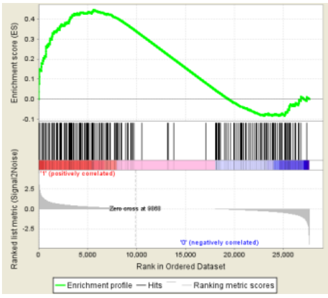

APOPTOSIS

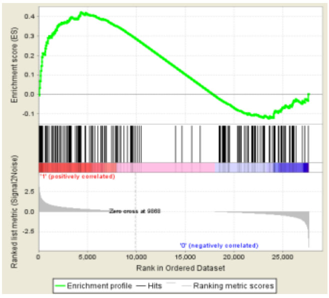

e

NAME	SIZE	ES	NES	NOM p-val	FDR q-val	FWER p-val
HALLMARK_E2F_TARGETS	196	-0.69755	-2.25581	0	0	0
HALLMARK_G2M_CHECKPOINT	196	-0.6202	-1.99825	0	0	0
HALLMARK_MYC_TARGETS_V2	58	-0.67903	-1.87902	0	0	0
HALLMARK_MYC_TARGETS_V1	197	-0.47315	-1.52051	0	0.018139	0.076
HALLMARK_DNA_REPAIR	142	-0.48056	-1.51752	0	0.015466	0.081
HALLMARK_MITOTIC_SPINDLE	199	-0.39022	-1.25594	0.01897	0.144262	0.695
HALLMARK_UV_RESPONSE_UP	154	-0.39203	-1.2472	0.048593	0.140425	0.722

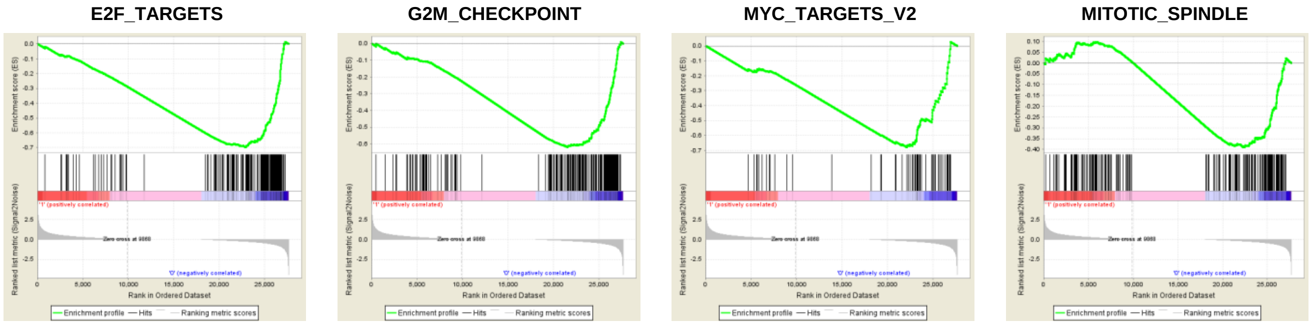

f

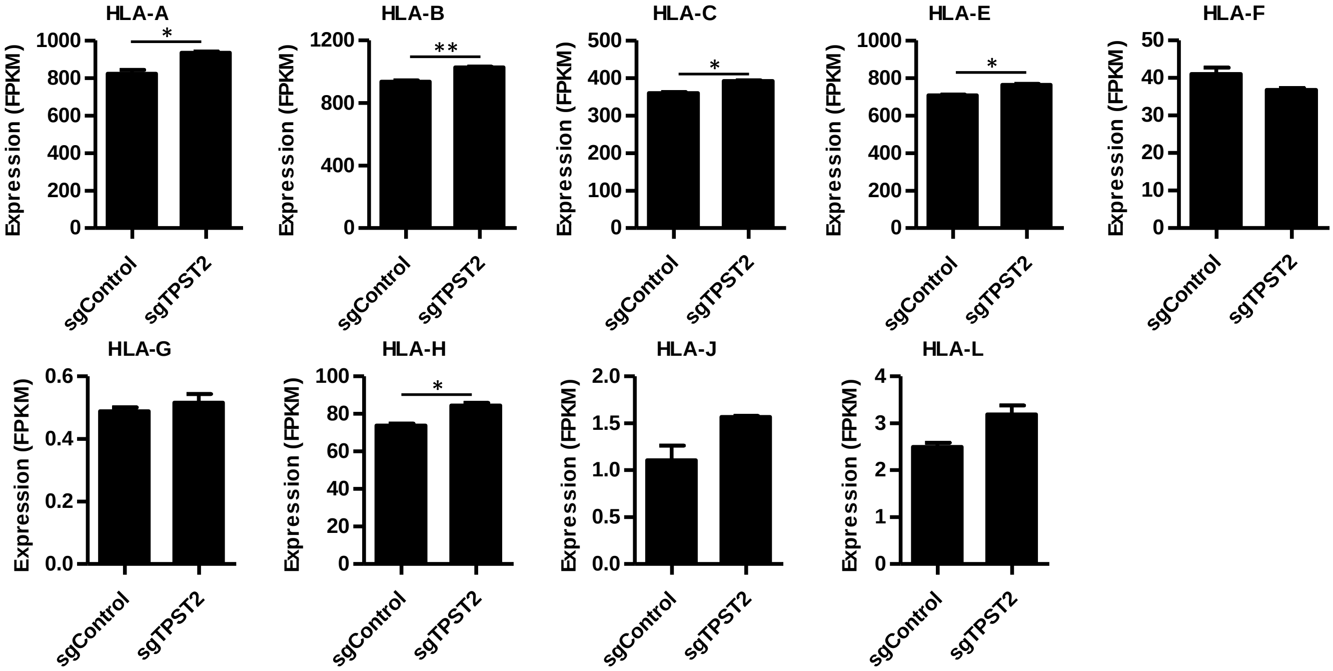

Supplementary Fig. S8. Transcriptomic analysis of TPST2-depleted breast cancer cells in the presence of IFNγ treatment. a, Volcano plot illustrating the relative mRNA expression levels of genes in RNA sequencing analysis of wild-type and TPST2 knock-down MBA-MD-231 cells. After treatment of 1 μg/ml IFNγ for 8 h, transcriptomic analysis was performed using RNA sequencing. Knock-down of TPST2 significantly up- and down-regulated 313 genes (red dots) and 225 genes (blue dots) compared with control cells, respectively ($P < 0.05$ and Log_2[fold change] ≥ 1). **b**, Hallmark gene set analysis of genes that were significantly increased by knock-down of TPST2. The hallmark gene sets that were significantly enriched in the 313 genes that were up-regulated by TPST2 knock-down in RNA sequencing are shown ($Q < 0.05$). **c**, KEGG pathway analysis of genes that were significantly increased by knock-down of TPST2. KEGG pathway gene sets that were significantly enriched in the 313 genes that were up-regulated by TPST2 knock-down in RNA sequencing are shown ($P < 0.05$). **d**, Gene set enrichment analysis (GSEA) for RNA sequencing from wild-type and TPST2 knock-down MBA-MD-231 cells in the presence of IFNγ. The upper table shows the significantly enriched gene sets in TPST2 knock-down cells ($P < 0.05$). ES: enrichment score, NES: normalized enrichment score, NOM p-val: nominal P-value, FDR q-val: false discovery rate Q-value, FWER q-val: family-wise error rate Q-value. The lower graphs show the enrichment plots of representative gene sets that were significantly enriched in TPST2-depleted cells ($P < 0.05$). On the x-axis, genes are ranked from the most upregulated to the most downregulated between TPST2 knock-down (left end; positively correlated) and control (right end; negatively correlated) cells. The y-axis shows a running enrichment score for TPST2 knock-down. **e**, Gene set enrichment analysis (GSEA) for RNA sequencing from wild-type and TPST2 knock-down MBA-MD-231 cells in the presence of IFNγ. The upper table shows the significantly enriched gene sets in TPST2 wild-type cells compared to TPST2 knock-down cells. The lower graphs show the enrichment plots of representative gene sets that were significantly enriched in TPST2 wild-type cells compared to TPST2 knock-down cells. On the x-axis, genes are ranked from the most upregulated to the most downregulated between TPST2 knock-down (left end; positively correlated) and control (right end; negatively correlated) cells. The y-axis shows a running enrichment score for TPST2 knock-down. **f**, Expression levels of major histocompatibility complex (MHC) class I genes in TPST2 knock-down MBA-MD-231 cells in the presence of IFNγ. The mRNA expressions levels were estimated as fragments per kilobase of transcript per million (FPKM) values from RNA sequencing data. Asterisks indicate significant differences (**, $P < 0.01$; *, $P < 0.05$).

Supplementary Figure S9

a

Sulfinator

E-cutoff value is 55

Protein / sequence name	Position	E-value	Sequence
INGR1_HUMAN P15260	84	[23]	NISHHYC-NISD----H<br>+++++Y+ + ++ +

Sequence(s) processed:**1**

Sulfated tyrosines detected: **1** (of 18)

Number of proteins with at least one hit: **1**

b

Protein name:INGR1 (504 aa)

Position	Flanking residue	ASA	SVM Probability
161	QEVDYDPET	0.3828	0.951276

Supplementary Fig. S9. Prediction of tyrosine sulfation sites in interferon gamma receptor 1 (IFNGR1) protein. a, **b**, Database search results of tyrosine sulfation of IFNGR1 protein from Sufinator (https://web.expasy.org/sulfinator/, **a**) and SulfoSite (http://sulfosite.mbc.nctu.edu.tw/, **b**).

Supplementary Figure S10

a

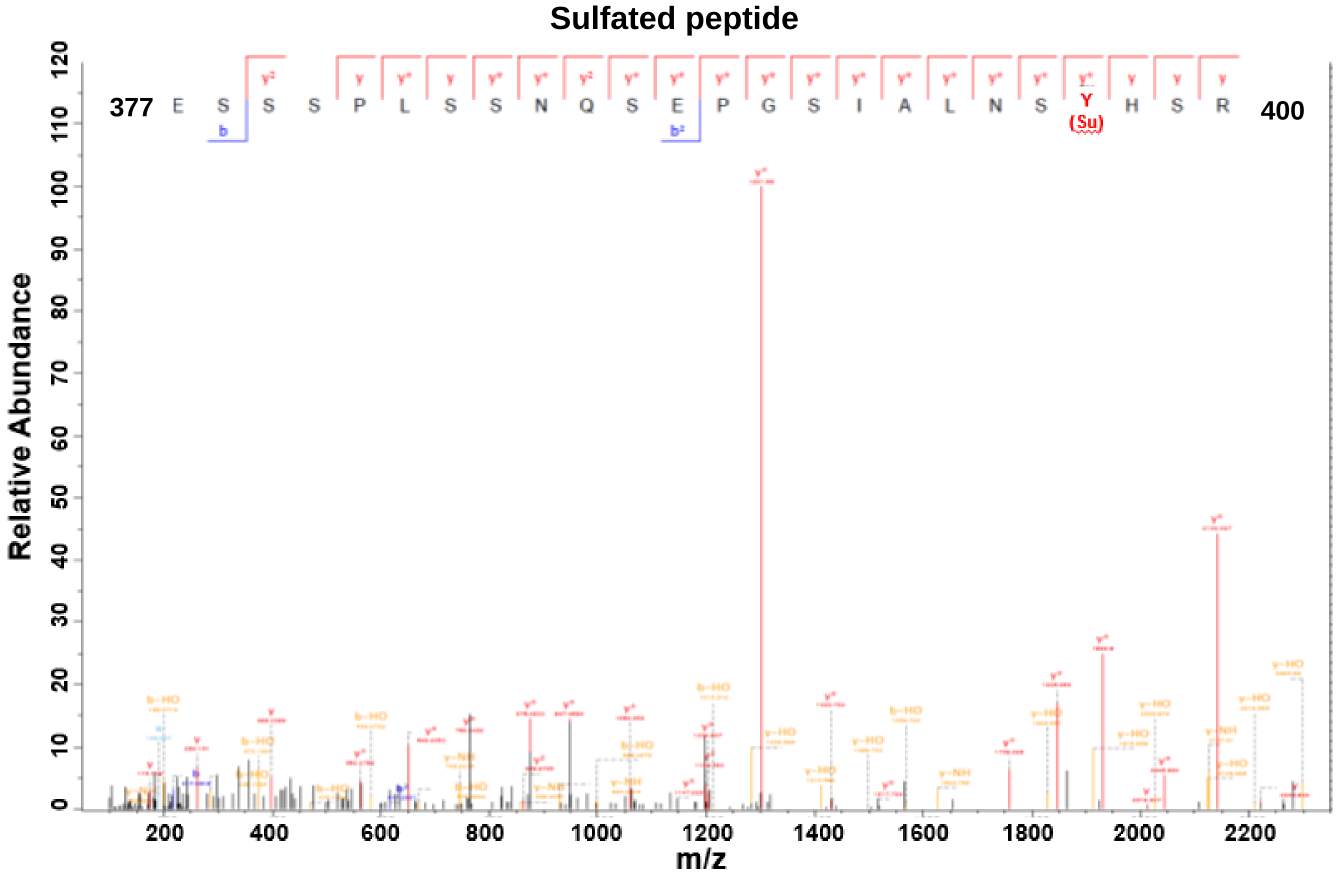

b

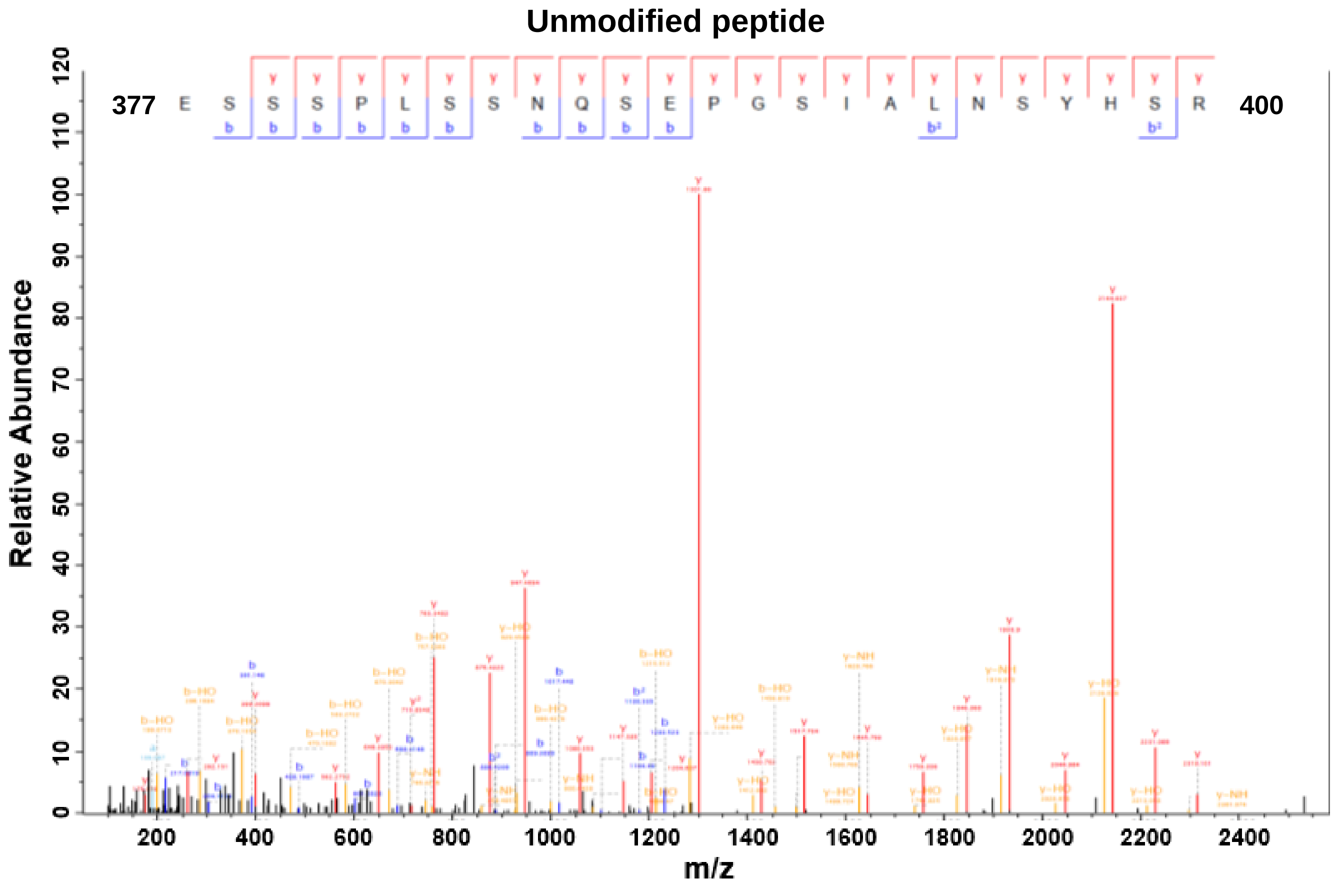

c

```
1    MALLFLLPLVMQGVSRAEMGTADLGPSSVPTPTNVTIESYNMNPIVYWEYQIMPQVPVFT  60
61   VEVKNYGVKNSEWIDACINISHHYCNISDHVGDPSNSLWVRVKARVGQKESAYAKSEEFA 120
121  VCRDGKIGPPKLDIRKEEKQIMIDIFHPSVFVNGDEQEVDYDPETTCYIRVYNVYVRMNG 180
181  SEIQYKILTQKEDDCDEIQCQLAIPVSSLNSQYCVSAEGVLHVWGVTTEKSKEVCITIFN 240
241  SSIKGSLWIPVVAALLLFLVLSLVFICFYIKKINPLKEKSIILPKSLISVVRSATLETKP 300
301  ESKYVSLITSYQPFSLEKEVVCEEPLSPATVPGMHTEDNPGKVEHTEELSSITEVVTTEE 360
361  NIPDVVPGSHLTPIERESSSPLSSNQSEPGSIALNSYHSRNCSESDHSRNGFDTDSSCLE 420
421  SHSSLSDSEFPPNNKGEIKTEGQELITVIKAPTSFGYDKPHVLVDLLVDDSGKESLIGYR 480
481  PTEDSKEFS
```

d

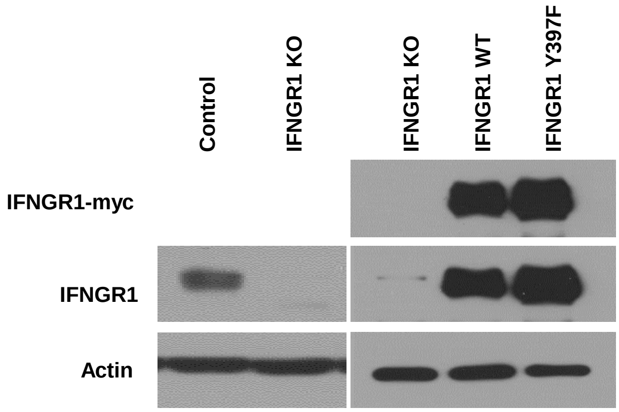

Supplementary Fig. S10. Identification of tyrosine sulfation sites in IFNGR1 protein. a, b, Determination of tyrosine sulfation sites in IFNGR1 protein by mass spectrometry. Mass spectrum of IFNGR1 peptides containing a tyrosine sulfation site in tyrosine-modified (**a**) and unmodified (**b**) protein were demonstrated. **c**, Amino acid sequence of IFNGR1 protein. Peptides detected by mass spectrometry were marked in green color. Phosphorylation and sulfation sites detected by mass spectrometry were marked as ‘P’ and ‘S’, respectively. **d**, Overexpression of wild-type and Y397F mutant IFNGR1 in IFNGR1 knock-out MBA-MD-231 cells. IFNGR1 knock-out cells were generated using CRISPR/Cas9. Wild-type and mutant IFNGR1 were overexpressed in MDA-MB-231 cells for 24 h and the expression of IFNGR1 was detected by western blot.

Supplementary Figure S11

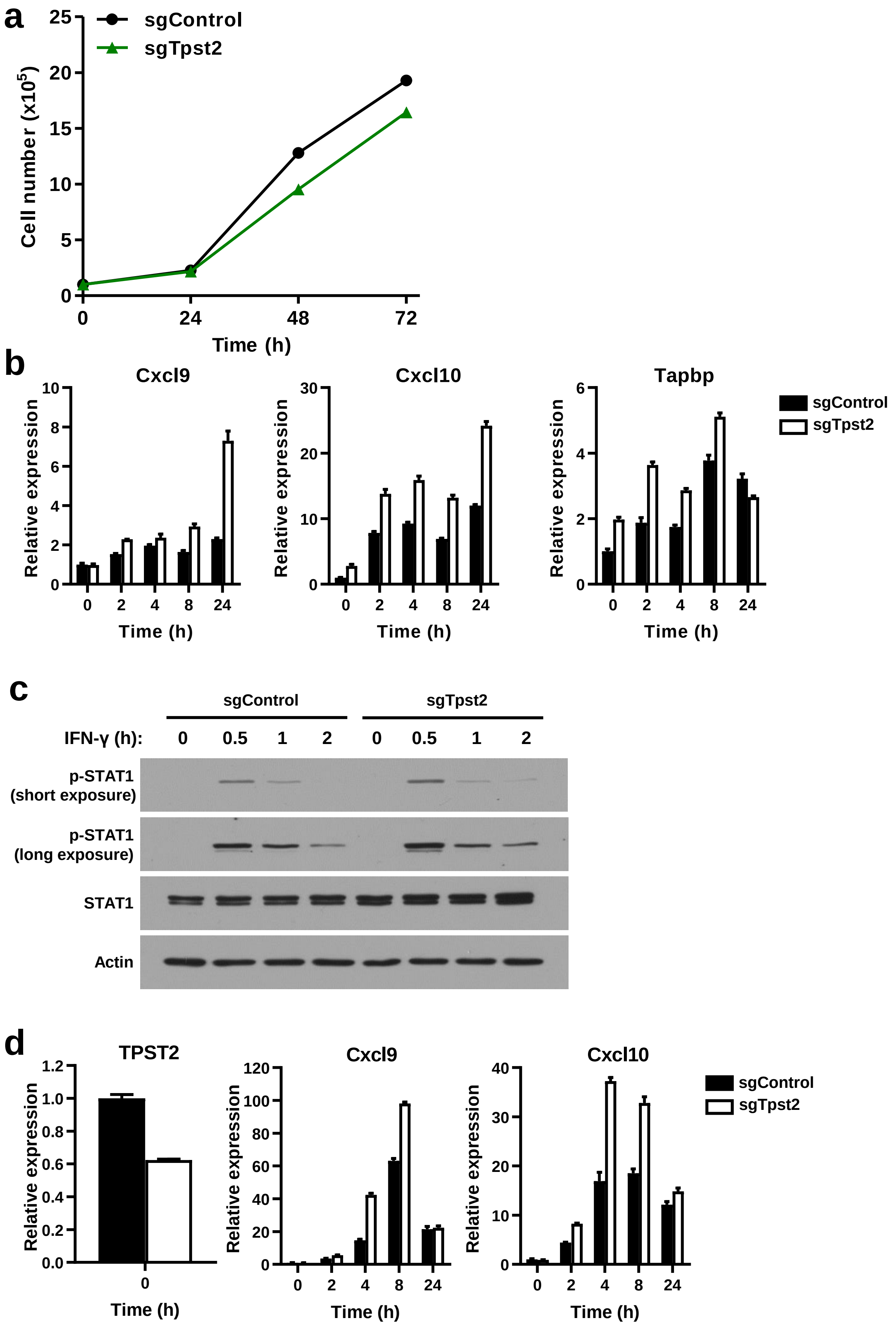

e

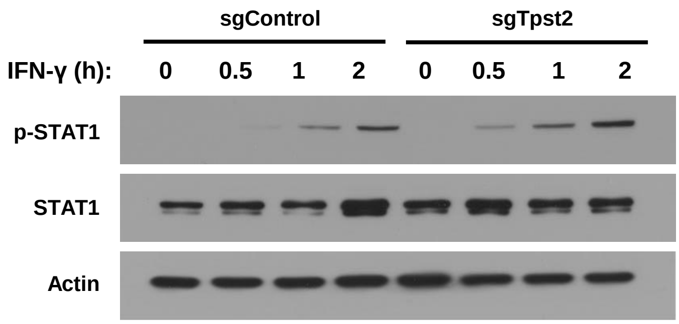

f

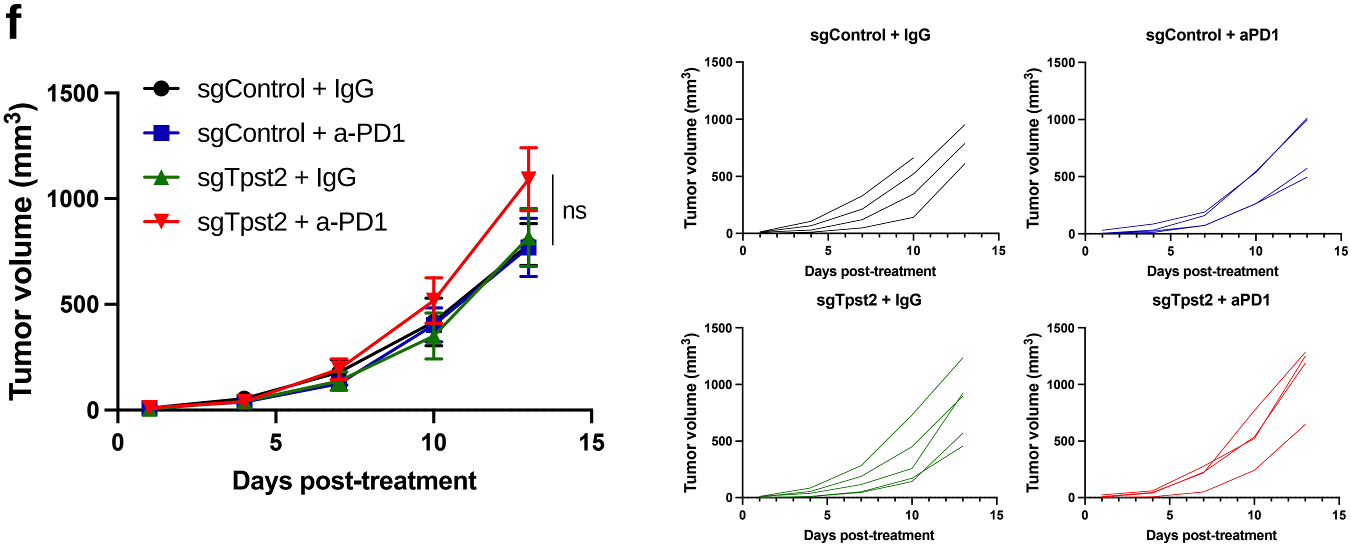

Supplementary Fig. S11. Tpst2 knock-down suppresses tumor growth through immune-mediated mechanism. a, Representative control MC38 or Tpst2 knock-down MC38 cell growth *in vitro*. **b**, Enhanced expression of IFNγ-responsive genes in Tpst2 knock-down MC38 cells. After serum starvation for 24 h, cells were treated with 10 ng/ml mouse IFNγ and the expression levels of IFNγ-responsive genes were estimated by real-time PCR at indicated time points. **c**, Enhanced phosphorylation of Stat1 in Tpst2 knock-down MC38 cells. After serum starvation for 24 h, cells were treated with 1 ng/ml IFNγ for indicated time. The phosphorylation levels of Stat1 were evaluated by western blotting. **d**, Enhanced expression of IFNγ-responsive genes in Tpst2 knock-down 4T1 cells. After serum starvation for 24 h, cells were treated with 10 ng/ml mouse IFNγ and the expression levels of IFNγ-responsive genes were estimated by real-time PCR at indicated time points. **e**, Enhanced phosphorylation of Stat1 in Tpst2 knock-down 4T1 cells. After serum starvation for 24 h, cells were treated with 1 ng/ml IFNγ for indicated time. The phosphorylation levels of Stat1 were evaluated by western blotting. **f**, Representative control MC38 or Tpst2 knock-down MC38 tumor growth curves with or without anti-PD1 in nude mice (CD8-depletion condition); n = 4-5 mice per group.

Supplementary Figure S12

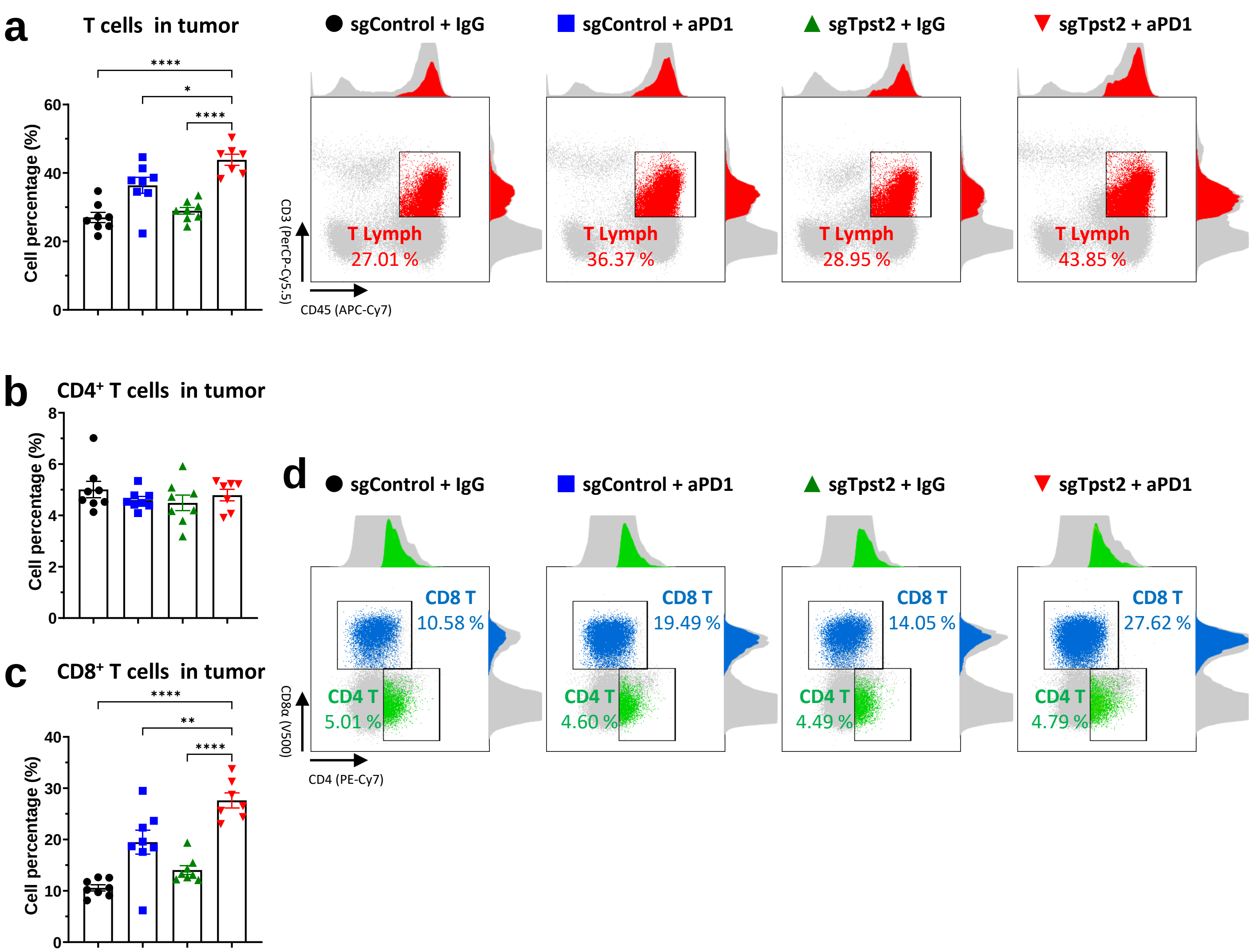

Supplementary Fig. S12. Immune cell profiling of tumor tissues from syngeneic mouse model. a, Percentage of total T cell in tumor tissues by flow cytometry analysis from control MC38 or TPST2 knock-down MC38-bearing mice with or without anti-PD1; n = 8 mice per group (left). The dot-plot represents population of total T cell (CD45$^+$ CD3$^+$) through CD45 and CD3 expression in each group (right). Gray background represents total immune cells and red dot represents T cell, and the above-attached and right-attached histograms represent CD45 expression and CD3 expression of each group, respectively. **b, c**, Percentage of total CD4$^+$ T cell (**b**) and CD8$^+$ T cell (**c**) in tumor tissues by flow cytometry analysis from control MC38 or TPST2 knock-down MC38-bearing mice with or without anti-PD1; n = 8 mice per group. **d**, The dot-plot represents population of CD4$^+$ T cells and CD8$^+$ T cells through CD4 and CD8 expression in each group. Gray background represents total immune cells, green dot represents CD4$^+$ T cell and blue dot represents CD8$^+$ T cell. The above-attached and right-attached histograms represent CD4 expression and CD8 expression of each group, respectively.

Supplementary Figure S13

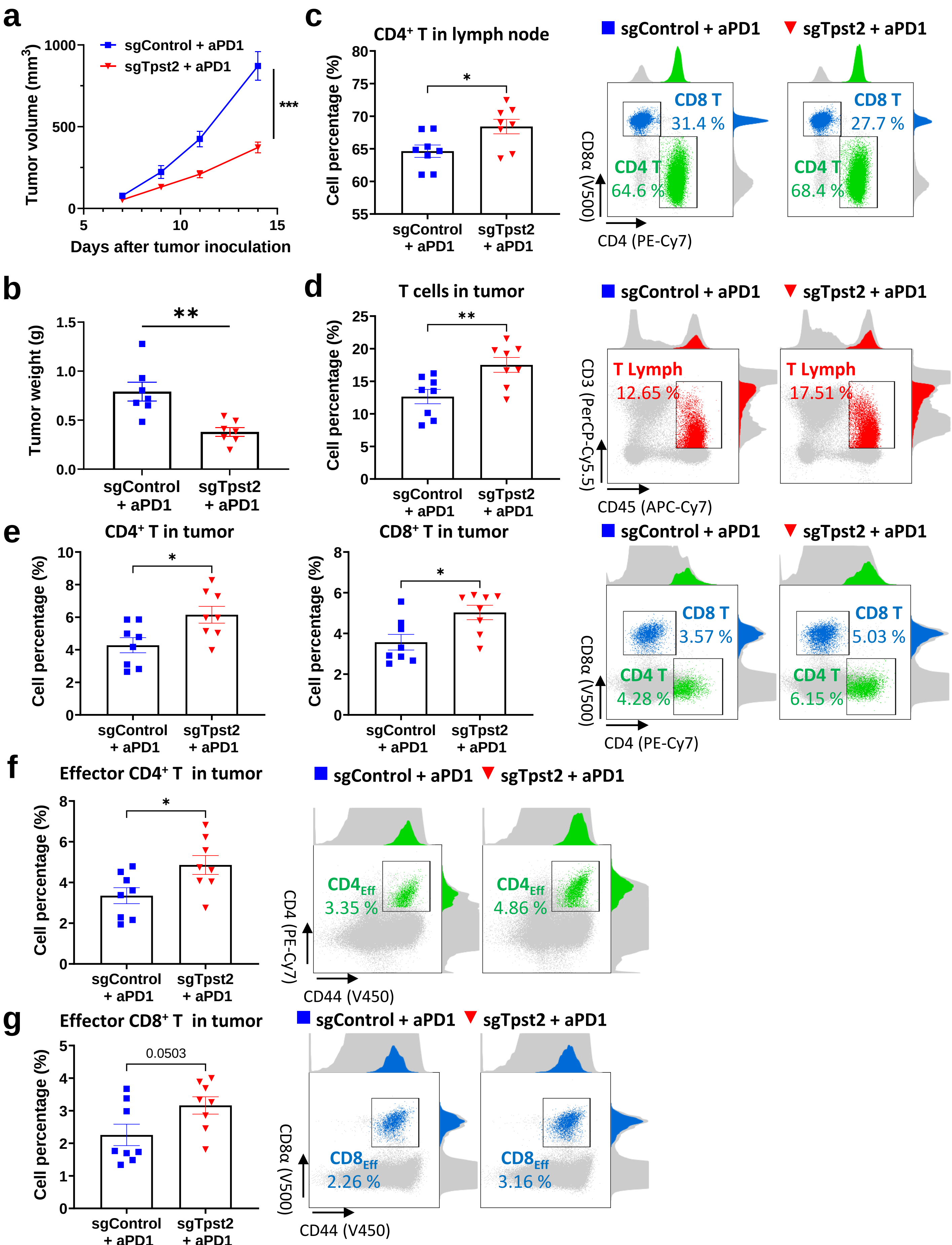

Supplementary Fig. S13. Synergistic enhancement of tumor reduction and immunity by TPST2 inhibition and anti-PD1 therapy. **a**, Growth curves of control MC38 and TPST2 knock-down MC38 tumors in mice treated with anti-PD1; n = 7 mice per group. **b**, Tumor weights at 18 days post-injection in a syngeneic mouse model; n = 7 mice per group. **c**, Flow cytometry analysis of the percentage of total $CD4^+$ T cells in tumor-draining lymph nodes from control and TPST2 knock-down MC38-bearing mice treated with anti-PD1; n = 8 mice per group (left). Dot plots show $CD4^+$ (green dots) and $CD8^+$ (blue dots) T cell populations, with histograms detailing CD4 and CD8 expression (right). The gray background indicates total lymphocytes. **d**, Percentage of total T cells ($CD45^+$ $CD3^+$) in tumor tissues analyzed by flow cytometry; n = 8 mice per group (left). Dot plots with red dots represent T cell populations, accompanied by histograms for CD45 and CD3 expression (right), against a gray background of total immune cells. **e**, Analysis of $CD4^+$ (left) and $CD8^+$ T cells (middle) percentages in tumor tissues; n = 8 mice per group. Right panel dot plots detail $CD4^+$ (green) and $CD8^+$ (blue) populations, with corresponding CD4 and CD8 expression histograms. **f**, **g**, Flow cytometry analysis of effector $CD4^+$ T cells (**f**) and $CD8^+$ T cells (**g**) percentages in tumor tissues; n = 8 mice per group (left). Dot plots illustrate effector $CD4^+$ (**f**) and $CD8^+$ (**g**) populations (green dots), with histograms for CD4 or CD8 and CD44 expression (right), set against a gray background of total lymphocytes.

Supplementary Figure S14

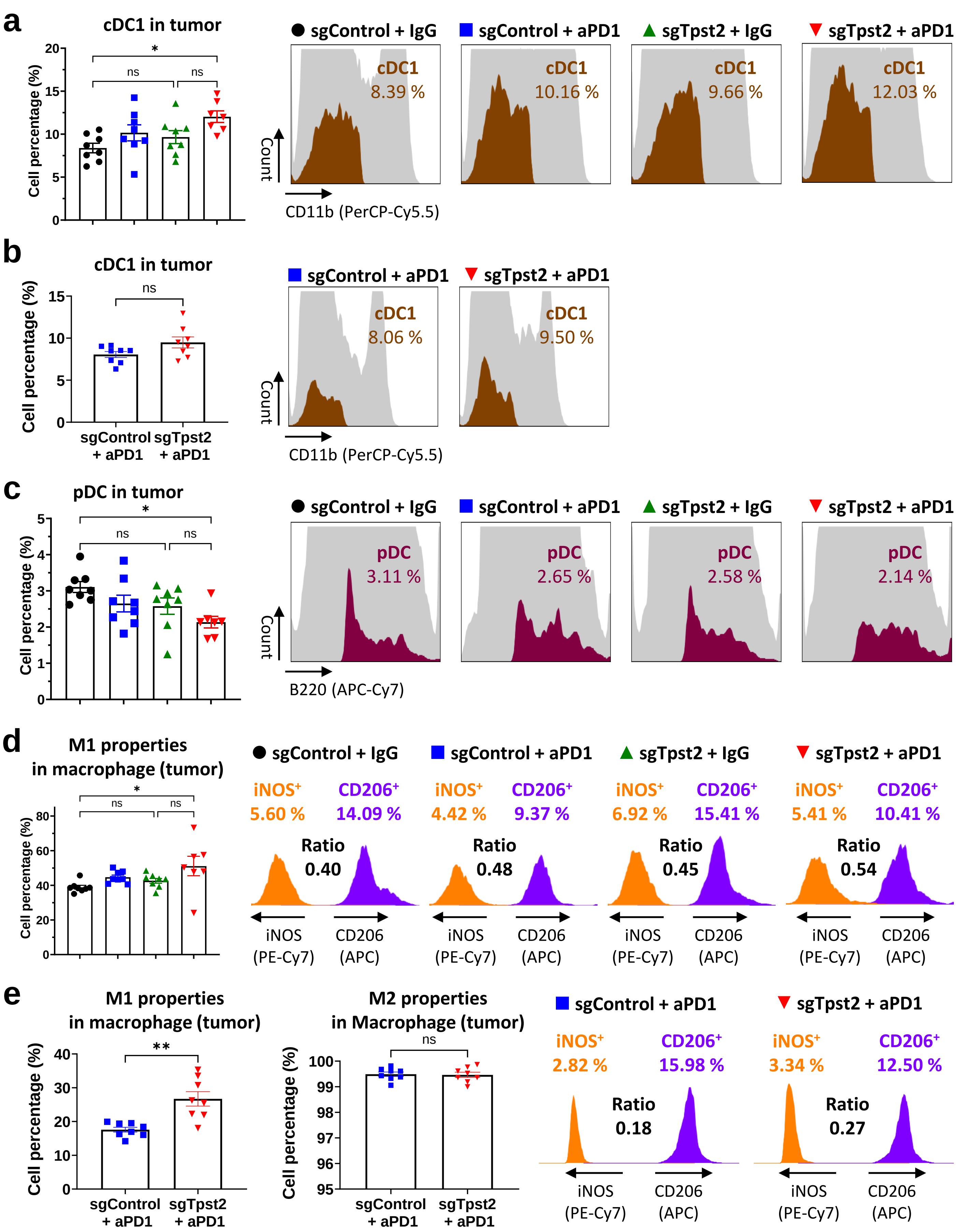

Supplementary Fig. S14. Myeloid immune cell profiling in tumor tissues. **a**, Percentage of cDC1 in tumor tissues by flow cytometry analysis from control MC38 or TPST2 knock-down MC38-bearing mice with or without anti-PD1 (4 groups); n = 8 mice per group (left). The histogram represents CD11b expression in cDC1 population in each group (right). Gray background represents CD11b expression of total immune cells and brown histogram represents CD11b expression of cDC1. **b**, Percentage of cDC1 tumor tissues by flow cytometry analysis from control MC38 + anti-PD1 versus TPST2 knock-down MC38 + anti-PD1 (2 groups); n = 8 mice per group (left). The histogram represents CD11b expression in cDC1 population in each group (right). Gray background represents CD11b expression of total immune cells and brown histogram represents CD11b expression of cDC1. **c**, Percentage of pDC in tumor tissues by flow cytometry analysis from control MC38 or TPST2 knock-down MC38-bearing mice with or without anti-PD1; n = 8 mice per group (right). The histogram represents B220 expression in pDC population in each group (left). Gray background represents B220 expression of total immune cells and violet histogram represents B220 expression of pDC. **d**, Percentage of macrophage with M1 properties in tumor tissues by flow cytometry analysis from control MC38 or TPST2 knock-down MC38-bearing mice with or without anti-PD1 (4 groups); n = 8 mice per group (right). The histograms represent ratio of M1-like and M2-like macrophages in each group (left). Orange histogram represents iNOS expression of M1-like macrophages and purple histogram represents CD206 expression of M2-like macrophages. **e**, Percentage of macrophage with M1 properties and macrophage with M2 properties in tumor tissues by flow cytometry analysis from control MC38 + anti-PD1 versus TPST2 knock-down MC38 + anti-PD1 (2 groups); n = 8 mice per group (right). The histograms represent ratio of M1-like and M2-like macrophages in each group (left). Orange histogram represents iNOS expression of M1-like macrophages and purple histogram represents CD206 expression of M2-like macrophages.

Supplementary Figure S15

a **sgControl vs sgTpst2 in IgG group**

NAME	SIZE	ES	NES	NOM p-val	FDR q-val	FWER p-val
GOBP_ATP_SYNTHESIS_COUPLED_ELECTRON_TRANSPORT	78	-0.65073234	-2.1917264	0	0.003090131	0.003
GOBP_OXIDATIVE_PHOSPHORYLATION	133	-0.5926224	-2.1357577	0	0.003075771	0.006
GOBP_AEROBIC_ELECTRON_TRANSPORT_CHAIN	64	-0.67306477	-2.1253555	0	0.002728244	0.008
GOBP_CELLULAR_GLUCURONIDATION	19	-0.85279286	-2.0998988	0	0.003310312	0.013
GOBP_PROTON_MOTIVE_FORCE_DRIVEN_ATP_SYNTHESIS	55	-0.67068374	-2.0983407	0	0.00264825	0.013
GOBP_URONIC_ACID_METABOLIC_PROCESS	24	-0.7789516	-2.037117	0	0.005786405	0.033
GOBP_DEFENSE_RESPONSE_TO_GRAM_NEGATIVE_BACTERIUM	117	-0.5725268	-2.0241702	0	0.007167457	0.048
**GOBP_ANTIGEN_PROCESSING_AND_PRESENTATION_OF_PEPTIDE_OR_POLYSACCHARIDE_ANTIGEN_VIA_MHC_CLASS_II**	27	-0.73856336	-2.0232542	0	0.006400488	0.049
GOBP_AEROBIC_RESPIRATION	182	-0.5247258	-1.9510074	0	0.020385452	0.168
GOBP_POSITIVE_REGULATION_OF_T_HELPER_1_TYPE_IMMUNE_RESPONSE	19	-0.78488284	-1.9227517	0	0.030291688	0.257
**GOBP_ANTIGEN_PROCESSING_AND_PRESENTATION_OF_EXOGENOUS_PEPTIDE_ANTIGEN_VIA_MHC_CLASS_II**	21	-0.75460345	-1.9119328	0	0.032717794	0.303
GOBP_ELECTRON_TRANSPORT_CHAIN	112	-0.53352284	-1.8809893	0	0.04703966	0.42
GOBP_RESPONSE_TO_PROTOZOAN	46	-0.6140074	-1.8752074	0	0.04664849	0.442
GOBP_POSITIVE_REGULATION_OF_HUMORAL_IMMUNE_RESPONSE	23	-0.7194715	-1.8716053	0.00295858	0.045261923	0.454
GOBP_NADH_DEHYDROGENASE_COMPLEX_ASSEMBLY	62	-0.5789351	-1.867148	0	0.04481482	0.479
GOBP_ANTIGEN_PROCESSING_AND_PRESENTATION_OF_PEPTIDE_ANTIGEN	76	-0.5593476	-1.8590354	0	0.048176628	0.531
GOBP_MITOCHONDRIAL_ELECTRON_TRANSPORT_NADH_TO_UBIQUINONE	29	-0.6796397	-1.8518362	0	0.050307885	0.568

b **sgControl vs sgTpst2 in aPD1 group**

NAME	SIZE	ES	NES	NOM p-val	FDR q-val	FWER p-val
GOBP_ATP_SYNTHESIS_COUPLED_ELECTRON_TRANSPORT	78	-0.59487903	-2.0867732	0	0.00544898	0.013
**GOBP_ANTIGEN_PROCESSING_AND_PRESENTATION_OF_EXOGENOUS_PEPTIDE_ANTIGEN_VIA_MHC_CLASS_II**	21	-0.78001964	-2.028164	0	0.007380776	0.042
GOBP_AEROBIC_ELECTRON_TRANSPORT_CHAIN	64	-0.61444134	-2.0335772	0	0.007961865	0.036
GOBP_CELLULAR_GLUCURONIDATION	19	-0.8105933	-2.0446868	0.002197802	0.009200143	0.032
**GOBP_ANTIGEN_PROCESSING_AND_PRESENTATION_OF_PEPTIDE_OR_POLYSACCHARIDE_ANTIGEN_VIA_MHC_CLASS_II**	27	-0.7455678	-2.099848	0	0.009202256	0.011
GOBP_MITOCHONDRIAL_ELECTRON_TRANSPORT_NADH_TO_UBIQUINONE	29	-0.70112354	-1.9996939	0	0.010377294	0.072
GOBP_URONIC_ACID_METABOLIC_PROCESS	24	-0.7262663	-1.984356	0	0.012150562	0.098
GOBP_OXIDATIVE_PHOSPHORYLATION	133	-0.5329519	-1.954472	0	0.018729968	0.162
GOBP_REGULATION_OF_ANTIGEN_RECEPTOR_MEDIATED_SIGNALING_PATHWAY	65	-0.5790279	-1.9335638	0	0.023957625	0.222
GOBP_ANTIGEN_PROCESSING_AND_PRESENTATION_OF_EXOGENOUS_ANTIGEN	58	-0.58177805	-1.909339	0	0.031708606	0.308
GOBP_PEPTIDE_ANTIGEN_ASSEMBLY_WITH_MHC_PROTEIN_COMPLEX	16	-0.76501113	-1.8833345	0.002272727	0.044602137	0.42
GOBP_NEGATIVE_REGULATION_OF_ANTIGEN_RECEPTOR_MEDIATED_SIGNALING_PATHWAY	31	-0.6631434	-1.8739814	0	0.047071133	0.469
GOBP_POSITIVE_REGULATION_OF_MYELOID_LEUKOCYTE_MEDIATED_IMMUNITY	53	-0.56337225	-1.8495286	0	0.050160818	0.608

c

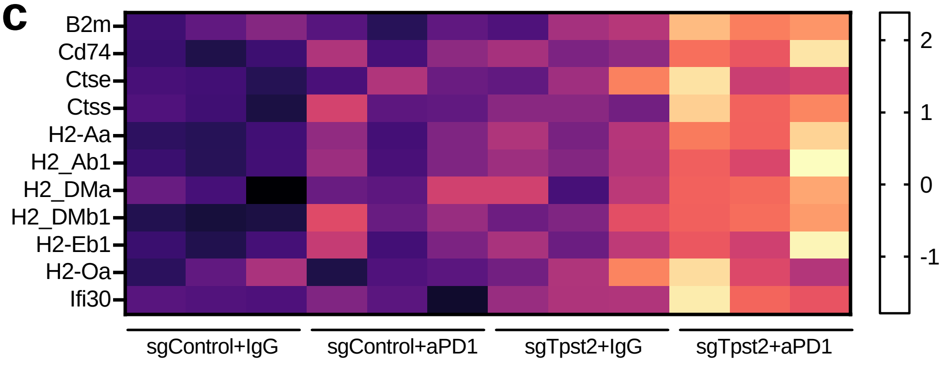

Supplementary Fig. S15. Tumor RNA sequencing analysis using syngeneic mouse model. a, **b**, Gene Set Enrichment Analysis (GSEA) outcomes based on Gene Ontology Biological Process. The tables show pathways enriched in TPST2 knock-down tumors compared to NT controls, detailing NES (Normalized Enrichment Score), NOM p-val (Nominal p-value), FDR q-val (False Discovery Rate q-value), and FWER p-val (Family-Wise Error Rate p-value). Panel (**a**) delineates comparisons between NT + IgG and TPST2 knock-down + IgG groups, while Panel (**b**) focuses on NT + anti-PD1 versus TPST2 knock-down + anti-PD1 groups, highlighting pathways with FDR q-val less than 0.05. **c**, Heatmap of antigen processing and presentation gene expression. Showcases expression levels of 11 genes associated with the antigen processing and presentation pathway, identified in the GSEA from Figure 6A. Expression levels, quantified in TPM, are normalized to z-scores to enable comparative analysis across genes.

Supplementary Figure S16

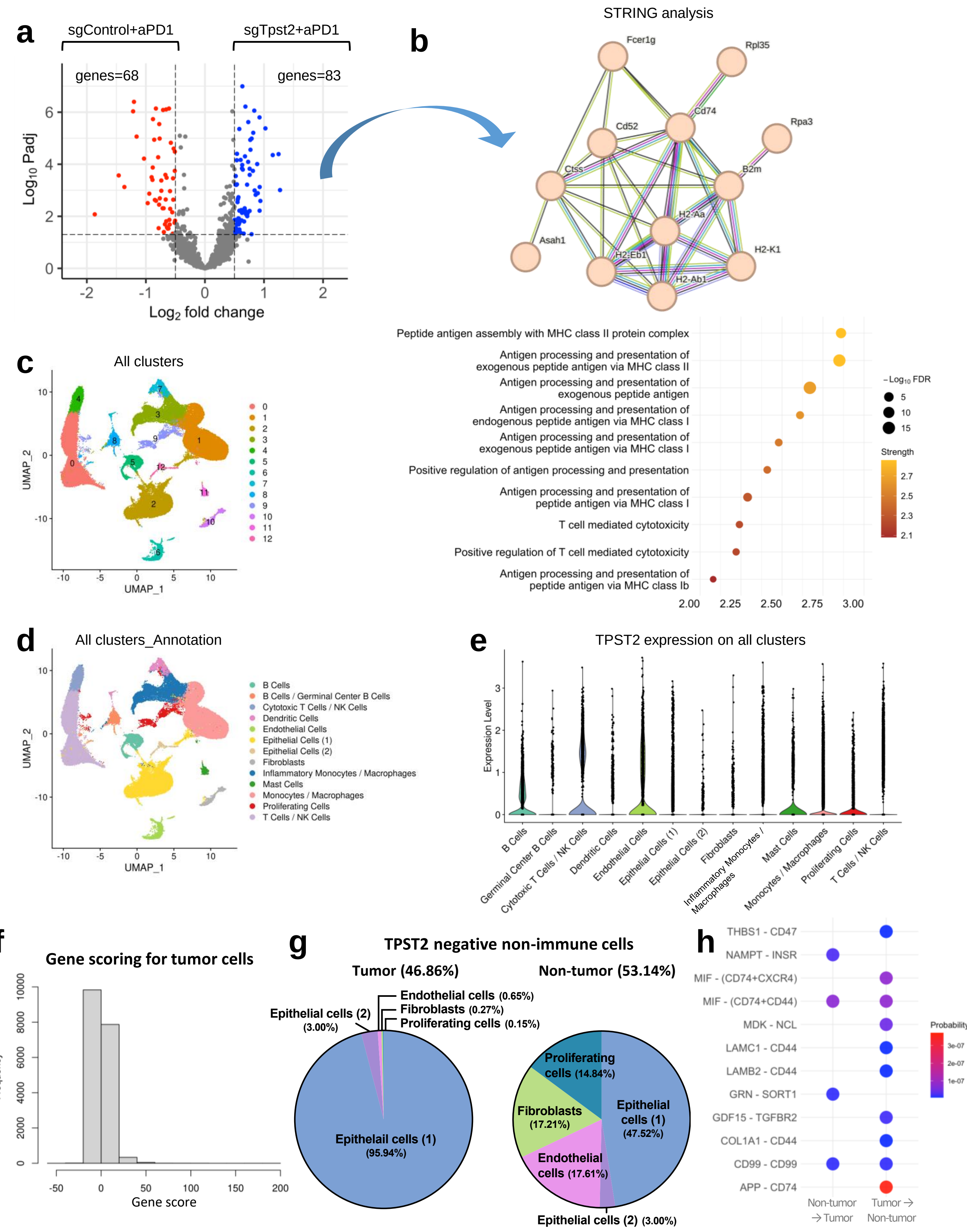

Supplementary Fig. S16. Tumor RNA sequencing of syngeneic mouse model and single-cell analysis of TPST2 expression of human lung adenocarcinoma. **a**, Volcano plots of differential gene expression between the NT + anti-PD1 group and the TPST2 knock-down + anti-PD1 group. DEGs were identified using a threshold of absolute |Log2FoldChange| > 0.5 and Padj < 0.05. **b**, The STRING analysis, focused on genes upregulated in the TPST2 knock-down + anti-PD1 group compared to the NT + anti-PD1 group, is illustrated. The top panel displays the protein-protein interaction network within main cluster 2, while the bottom panel outlines the pathways predominantly formed by these interactions. The x-axis signifies the strength of interaction, and the dot size reflects the -log10FDR values. **c**, Cellular profiling via UMAP in 12 lung adenocarcinoma patients. UMAP analysis illustrates the segregation of 74,888 cells into 13 unique clusters via unsupervised clustering. **d**, Annotation of unsupervised clusters based on the most distinct markers for each cluster. **e**, The distribution of TPST2 expression within all clusters. **f**. Gene scoring for tumor and non-tumor cells in TPST2-negative non-immune cells. It was performed using representative tumor markers (EPCAM, KRT8, KRT18, MUC1, TP53, CEACAM5, SOX2) and lung cancer-specific markers (TTF1, NAPSA, CDH1). Cells with a score above 0 were classified as tumor cells, while those with a score below 0 were classified as non-tumor cells. **g**, Proportion of tumor and non-tumor cells within the TPST2-negative non-immune cell population. TPST2-negative non-immune cells consist of 6,743 (46.86%) tumor cells and 7,648 (53.14%) non-tumor cells. **h**. Significant interactions between tumor and non-tumor cells in the TPST2-negative non-immune cell population analyzed using CellChat, highlighting communication probabilities.

Supplementary Figure S17

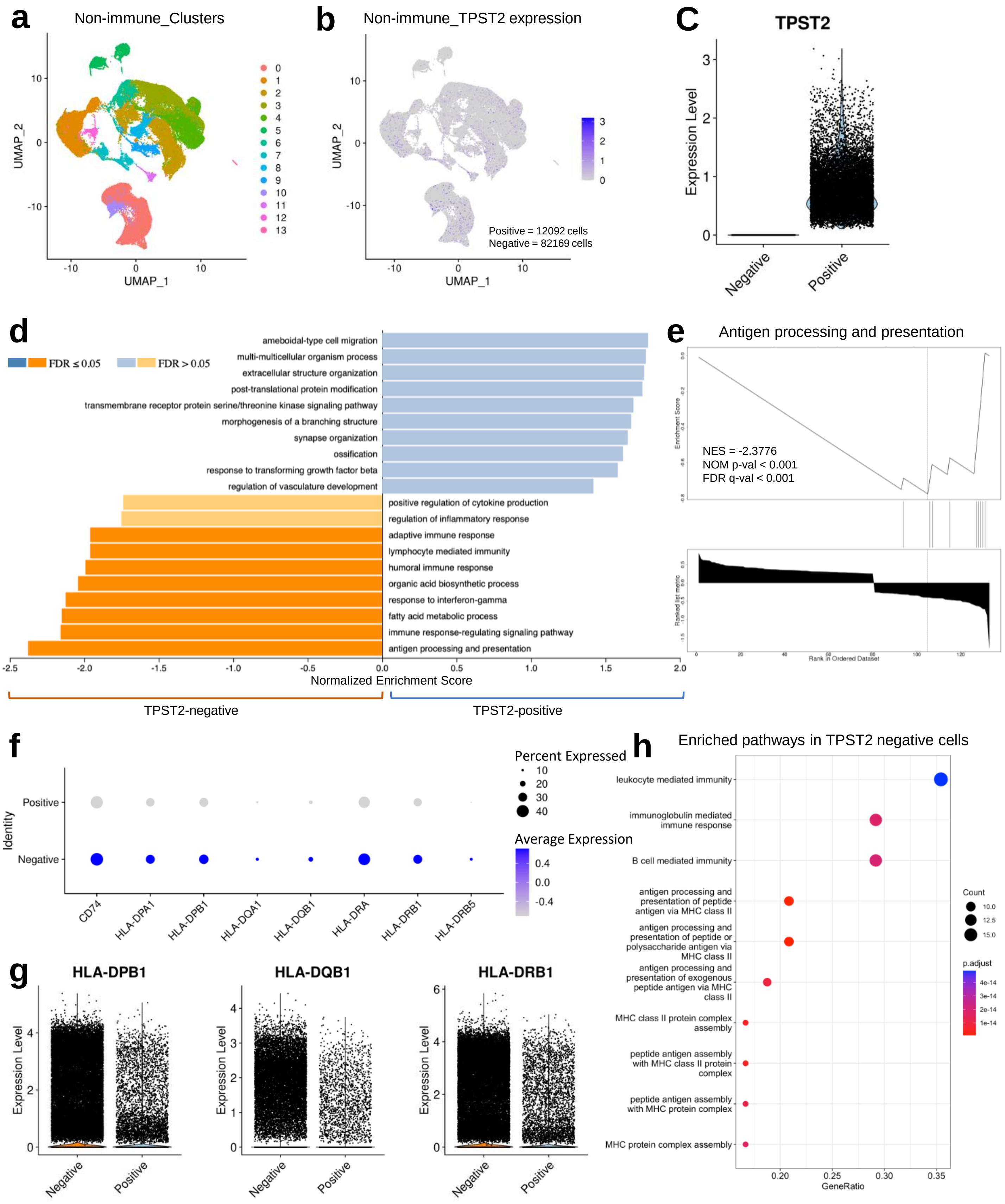

Supplementary Fig. S17. Single-cell analysis of TPST2 expression in human glioma. a, Non-immune cellular profiling via UMAP in 18 glioma patients. UMAP analysis illustrates the segregation of 94,261 non-immune cells into 14 unique clusters via unsupervised clustering. **b**, The expression of TPST2 across 14 distinct clusters. **c**, A violin plot compares the TPST2 expression between TPST2-negative and TPST2-positive non-immune cells. **d**, GSEA of TPST2-associated genomic alterations. GSEA, conducted with the WebGestalt tool, identifies significant pathways among 53 upregulated and 81 downregulated genes in TPST2-negative non-immune cells compared to TPST2-positive non-immune cells. **e**, A specific GSEA plot highlights the enrichment of antigen processing and presentation pathway in TPST2-negative cells. **f**, Comparative gene expression related to antigen processing and presentation pathway. This analysis compares the expression of genes, as identified in panel (**e**), between TPST2-positive and TPST2-negative non-immune cells. Dot size represents the percentage of cells expressing each gene, while dot color indicates the average expression level. **g**, Single-cell violin plots for antigen processing and presentation-related genes. Violin plots compare the expression of genes, as identified in panel (**e**), between TPST2-positive and TPST2-negative non-immune cells. Each dot within the plots represents an individual cell. **h**, Over-representation analysis for TPST2-negative non-immune cells compared to TPST2-positive non-immune cells.

Supplementary Figure S18

a

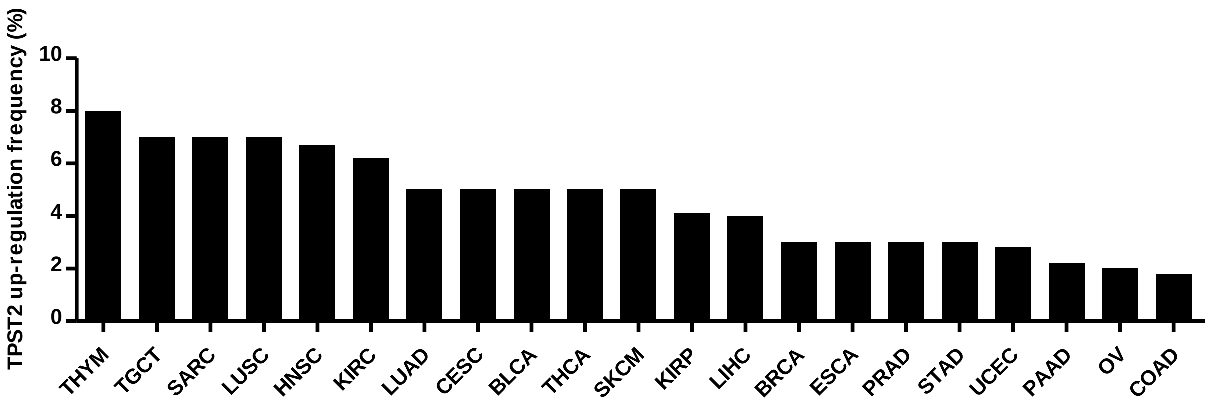

b

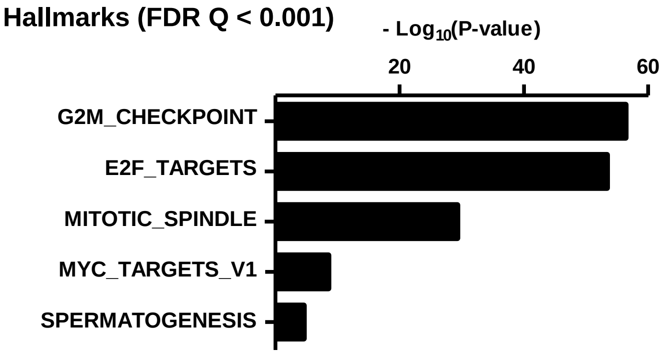

c

NAME	SIZE	ES	NES	NOM p-val	FDR q-val	FWER p-val
HALLMARK_E2F_TARGETS	168	-0.60361	-3.13382	0	0	0
HALLMARK_G2M_CHECKPOINT	164	-0.52767	-2.80226	0	0	0
HALLMARK_MYC_TARGETS_V1	164	-0.5172	-2.75489	0	0	0
HALLMARK_MYC_TARGETS_V2	44	-0.52145	-2.16599	0	0	0
HALLMARK_MITOTIC_SPINDLE	178	-0.31555	-1.69157	0	0.004044	0.017

d

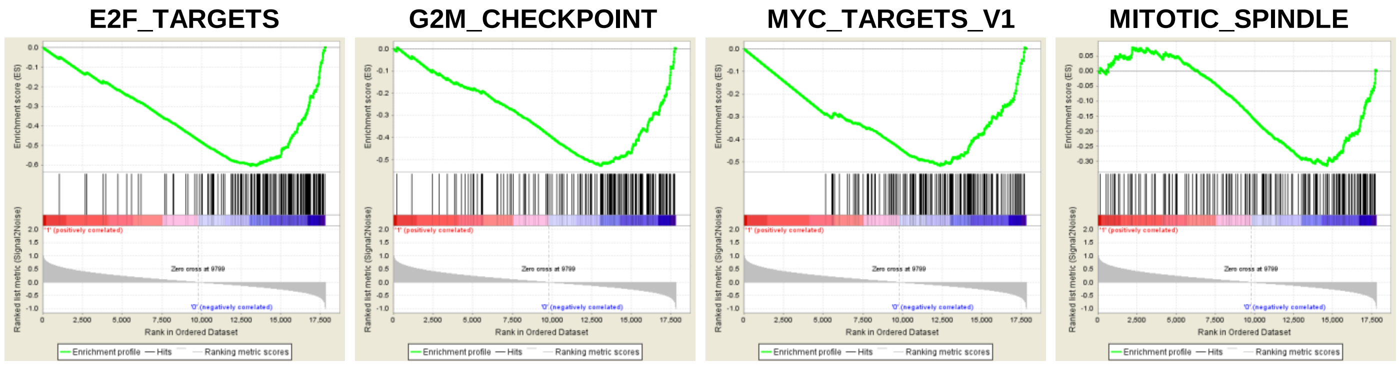

Supplementary Fig. S18. Enriched gene sets in breast cancer samples with low TPST2 expression. a, The proportion of patients with up-regulation of TPST2 mRNA (z-score threshold of 2) in tumor samples across the cancer types. Data were extracted from RNA seq V2 RSEM of The Cancer Genome Atlas (TCGA) PanCancer Atlas studies in cBioPortal (https://www.cbioportal.org/). **b**, Hallmark gene set analysis of genes with negative expression correlation with TPST2 in breast cancer. Hallmark gene set analysis was performed using 323 genes, of which expressions were negatively correlated with TPST2 expression (Spearman's correlation coefficient $\rho \leq -0.3$), and enriched gene sets are demonstrated ($Q < 0.001$). **c**, Gene set enrichment analysis (GSEA) for breast cancer tissue microarray data according to the TPST2 expression. Microarray data of 238 triple-negative breast cancer patients (GSE103091) were downloaded from the Gene Expression Omnibus (GEO) database (https://www.ncbi.nlm.nih.gov/geo). GSEA was performed between 20 samples with highest expression levels of TPST2 (TPST2_H) and 20 samples with lowest expression levels of TPST2 (TPST2_L). The significantly enriched gene sets in TPST2_L ($Q < 0.001$) were listed. ES: enrichment score, NES: normalized enrichment score, NOM p-val: nominal P-value, FDR q-val: false discovery rate Q-value. **d**, Enrichment plots of representative gene sets that were significantly enriched in TPST2_L group. On the x-axis, genes are ranked from the most upregulated to the most downregulated between TPST2_H (left end) and TPST2_L (right end) groups. The y-axis shows a running enrichment score for TPST2 expression.

Supplementary Figure S19

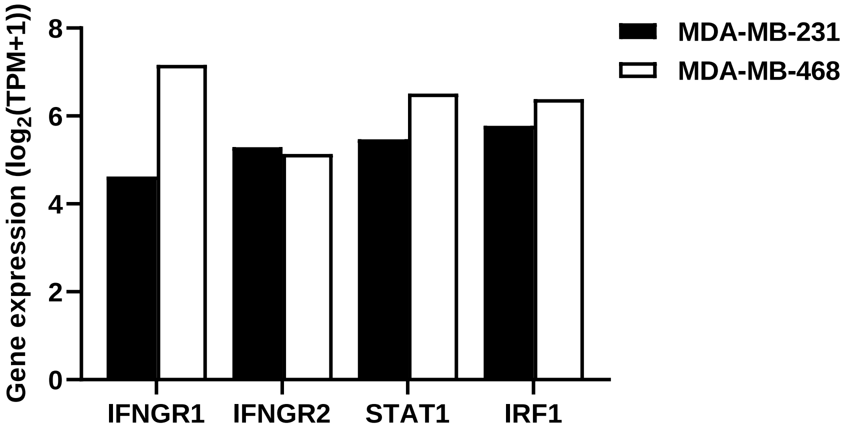

Supplementary Fig. S19. Expression of genes in the IFN-γ receptor-mediated pathway in human breast cancer cell lines. The expression levels of IFNGR1, IFNGR2, STAT1, and IRF1 in human breast cancer cell lines, MDA-MB-231 and MDA-MB-468, were estimated using log_2(TPM+1) values derived from RNA sequencing data in DepMap cell line database (https://depmap.org/portal/).
