# Supplementary material for: Genome-wide CRISPR screening identifies tyrosylprotein sulfotransferase-2 as a target for augmenting anti-PD1 efficacy
Source: Mol Cancer. 2024 Aug 2;23:155. doi: 10.1186/s12943-024-02068-x (PMC11295332; doi:10.1186/s12943-024-02068-x)
Supplement: Supplementary file 3 — Supplementary Material 3 [file 12943_2024_2068_MOESM3_ESM.pdf]

Supplementary Table S1. Sequences of sgRNAs and primers for real-time PCR.

1) Sequences of sgRNAs

Gene	Sequence
Human TPST1	5'-TACGTTCCTCTATCCGGTGA-3'
Human TPST2-1	5'-CTCCTCGCCGCAGCGCACCT'-3'
Human TPST2-2	5'-CCCCAGGATCGAGCGGTCCA-3'
Mouse Tpst2	5'-GATGCTCGGCGCCGACCACG-3'

2) Primers for real-time PCR

Gene	Forward primer	Reverse primer
Human IRF1	5'-CTCTGAAGCTACAACAGATGAG-3'	5'-GTAGACTCAGCCCAATATCCC-3'
Human TAP1	5'-AGGTACTGCTCTCCATCTAC-3'	5'-AGTGTAAGGGAGTCAACAGA-3'
Human TAP2	5'-ACGGCTGAGCTCGGATACCAC-3'	5'-CCTCGGCCCCAAAACTGC-3'
Human TAPBP	5'-ACCCTGGAGGTAGCAGGTCTTT-3'	5'-AATCCTTGCAGGTGGACAGGTAG-3'
Human TPST2	5'-TTTGACCTCAGCAGCTACCGT-3'	5'-AGGAAGTCGAGGATGAGCTTGA-3'
Human TPST1	5’-ATGTGGTCACGGTCAAGTAAAGAG-3’	5’-GCTCCCCATGCTTAACGATAAT-3’
Mouse Tpst2	5'-TGCCCGTGTACTATGAGCAG-3'	5'-GCTCGATCTTGGACAAGGAG-3'
Mouse Tap1	5'-CTGGCAACCAGCTACGGGT-3'	5'-TGAGAAAGAGGATGTGGTGGG-3'
Mouse Tapbp	5'-ACAAGGCCCCCAGAGTGT-3'	5'-GGAAGAAGTGGGATGCAAGA-3'
Mouse Cxcl9	5'-GGAACCCTAGTGATAAGGAATGCA-3'	5'-TGAGGTCTTTGAGGGATTTGTAGTG-3'
Mouse Cxcl10	5'-TCCTTGTCCTCCCTAGCTCA-3'	5'-ATAACCCCTTGGGAAGATGG-3'

Supplementary Table S2. Markers for cluster annotation in single cell analysis

Cluster	Cell type	Gene	Log_2FC (ave)	Adjusted p_val
0	T cells / NK cells	IL7R	3.1475	< 0.0001
		CCL5	1.9453	< 0.0001
		CD69	1.8035	< 0.0001
1	Monocytes / macrophages	APOC1	4.2866	< 0.0001
		C1QA	3.8489	< 0.0001
		C1QB	3.8218	< 0.0001
2	Epithelial cells (1)	WFDC2	4.7104	< 0.0001
		SCGB3A1	3.7211	< 0.0001
		SFTPB	3.7073	< 0.0001
3	Inflammatory monocytes / macrophages	S100A8	3.2833	< 0.0001
		IL1B	2.7318	< 0.0001
		FCN1	2.6911	< 0.0001
4	Cytotoxic T cells / NK cells	NKG7	3.8178	< 0.0001
		GZMB	2.5400	< 0.0001
		PRF1	2.4985	< 0.0001
5	B cells	IGLC2	8.2827	< 0.0001
		IGKC	8.1265	< 0.0001
		IGHA1	7.5555	< 0.0001
6	Endothelial cells	SPARCL1	4.8241	< 0.0001
		IGFBP7	4.3074	< 0.0001
		CLDN5	3.7557	< 0.0001
7	Dendritic cells	S100B	3.5266	< 0.0001
		HLA-DQB1	2.6891	< 0.0001
		HLA-DPB1	2.6238	< 0.0001
8	B cells / Germinal center B cells	MS4A1	2.8943	< 0.0001
		CD79A	2.0327	< 0.0001
		IGHM	1.8286	< 0.0001
9	Proliferating cells	STMN1	2.5723	< 0.0001
		HMGB2	2.3420	< 0.0001
		TOP2A	2.1468	< 0.0001
10	Fibroblasts	MGP	4.9371	< 0.0001
		LUM	4.2890	< 0.0001
		DCN	4.2764	< 0.0001
11	Mast cells	TPSB2	7.3396	< 0.0001
		TPSAB1	6.2630	< 0.0001
		CPA3	5.9213	< 0.0001
12	Epithelial cells (2)	CAPS	4.9333	< 0.0001
		SLPI	4.5096	< 0.0001
		C9orf24	4.1869	< 0.0001

Supplementary Table S3. The 482 genes, of which expressions were positively correlated with TPST2 expression.

Correlated Gene	Cytoband	Spearman's Correlation	p-Value	q-Value
TFIP11	22q12.1	0.503872582	9.91E-71	2.00E-66
SELENOM	22q12.2	0.496483555	2.06E-68	2.08E-64
EFEMP2	11q13.1	0.465282483	3.17E-59	2.13E-55
UBTD1	10q24.1-q24.2	0.462786428	1.57E-58	7.91E-55
RARRES2	7q36.1	0.457365881	4.83E-57	1.95E-53
IFFO1	12p13.31	0.457059282	5.86E-57	1.97E-53
PLPP7	9q34.13	0.451675545	1.65E-55	4.77E-52
COPZ2	17q21.32	0.450070176	4.43E-55	1.12E-51
EMP3	19q13.33	0.446514887	3.85E-54	8.63E-51
GAS2L1	22q12.2	0.442478933	4.34E-53	8.77E-50
PCOLCE	7q22.1	0.437481804	8.35E-52	1.53E-48
SERPINF1	17p13.3	0.435127437	3.30E-51	5.56E-48
MFRP	11q23.3	0.434748728	4.12E-51	6.39E-48
CHRD	3q27.1	0.432910061	1.19E-50	1.66E-47
TGFB1	19q13.2	0.432850417	1.24E-50	1.66E-47
TNFSF12	17p13.1	0.431581198	2.57E-50	3.18E-47
HTRA3	4p16.1	0.431508371	2.68E-50	3.18E-47
ROR2	9q22.31	0.430282925	5.41E-50	6.07E-47
LOXL1	15q24.1	0.426390408	4.96E-49	5.00E-46
HTRA1	10q26.13	0.4262274	5.43E-49	5.22E-46
PODNL1	19p13.12	0.425508646	8.15E-49	7.48E-46
TREM2	6p21.1	0.423979327	1.93E-48	1.69E-45
JDP2	14q24.3	0.423877907	2.04E-48	1.71E-45
PPM1M	3p21.2	0.420971139	1.03E-47	8.32E-45
MMP2	16q12.2	0.419828567	1.94E-47	1.51E-44
TOM1	22q12.3	0.419616018	2.18E-47	1.63E-44
MVP	16p11.2	0.417558023	6.78E-47	4.89E-44
CLEC11A	19q13.33	0.416956397	9.42E-47	6.56E-44
SYNDIG1	20p11.21	0.416804429	1.02E-46	6.89E-44
VENTX	10q26.3	0.414673256	3.27E-46	2.13E-43
SMPD1	11p15.4	0.412907776	8.52E-46	5.21E-43
GAS6	13q34	0.412510515	1.06E-45	6.26E-43
LILRA2	19q13.42	0.411597974	1.72E-45	9.95E-43
APOBR	16p12.1	0.410663957	2.85E-45	1.60E-42
COX7A1	19q13.12	0.410577093	2.98E-45	1.63E-42
NAALADL1	11q13.1	0.410225852	3.60E-45	1.91E-42
CTSK	1q21.3	0.409842719	4.42E-45	2.29E-42
PDLIM7	5q35.3	0.409278138	5.97E-45	2.94E-42
LAT2	7q11.23	0.409035227	6.80E-45	3.27E-42
ADAMTSL2	9q34.2	0.407119503	1.88E-44	8.83E-42
CAMK1	3p25.3	0.406931197	2.08E-44	9.53E-42
CYS1	2p25.1	0.406244412	2.99E-44	1.34E-41
PTGIR	19q13.32	0.406112297	3.20E-44	1.38E-41
SCARF2	22q11.21	0.406096256	3.23E-44	1.38E-41
NTNG2	9q34.13	0.406065252	3.28E-44	1.38E-41
CD99L2	Xq28	0.405518301	4.38E-44	1.80E-41
THY1	11q23.3	0.404584891	7.16E-44	2.83E-41
ALOX5AP	13q12.3	0.404208389	8.72E-44	3.38E-41
DOK1	2p13.1	0.403919455	1.01E-43	3.86E-41
C4A	6p21.33	0.403655908	1.16E-43	4.35E-41
PTH1R	3p21.31	0.399894031	8.23E-43	2.97E-40
CLEC2B	12p13.31	0.399287839	1.13E-42	3.99E-40
GLIS2	16p13.3	0.39917223	1.19E-42	4.12E-40
NNMT	11q23.2	0.399159269	1.20E-42	4.12E-40
COL1A1	17q21.33	0.398176133	1.99E-42	6.60E-40
LY86	6p25.1	0.39770447	2.54E-42	8.27E-40
MSC	8q13.3	0.397601494	2.68E-42	8.45E-40
NME8	7p14.1	0.397341708	3.06E-42	9.50E-40
CILP2	19p13.11	0.396498674	4.71E-42	1.40E-39
LYL1	19p13.13	0.395431037	8.11E-42	2.31E-39
FBLN1	22q13.31	0.395025647	9.97E-42	2.79E-39
SQOR	15q21.1	0.394725497	1.16E-41	3.17E-39
AEBP1	7p13	0.39389901	1.77E-41	4.63E-39

CMTM3	16q22.1	0.393671875	1.98E-41	5.12E-39
GPR1	2q33.3	0.392959282	2.84E-41	7.25E-39
COL6A2	21q22.3	0.392308075	3.94E-41	9.94E-39
DACT3	19q13.32	0.390871793	8.10E-41	1.97E-38
COL8A2	1p34.3	0.389957931	1.28E-40	3.07E-38
SCT	11p15.5	0.389364056	1.72E-40	4.04E-38
MXRA8	1p36.33	0.388710734	2.38E-40	5.53E-38
ILK	11p15.4	0.388168317	3.12E-40	7.15E-38
WBP1L	10q24.32	0.388045376	3.31E-40	7.52E-38
NDN	15q11.2	0.387821682	3.70E-40	8.30E-38
TYROBP	19q13.12	0.386830849	6.05E-40	1.34E-37
FGD3	9q22.31	0.386412371	7.43E-40	1.61E-37
CYP4Z1	1p33	0.386312277	7.81E-40	1.68E-37
MGAT1	5q35.3	0.385932215	9.42E-40	1.98E-37
FEZ1	11q24.2	0.385923751	9.45E-40	1.98E-37
EMILIN1	2p23.3	0.385915602	9.49E-40	1.98E-37
KIAA0930	22q13.31	0.385740371	1.03E-39	2.13E-37
TSPAN4	11p15.5	0.383920438	2.53E-39	5.10E-37
OLFML3	1p13.2	0.38326448	3.48E-39	6.89E-37
MRGPRF	11q13.3	0.382970696	4.02E-39	7.87E-37
02-Mar	19p13.2	0.382782925	4.40E-39	8.46E-37
C1QTNF3	5p13.2	0.38235011	5.43E-39	1.03E-36
DCN	12q21.33	0.382122743	6.07E-39	1.14E-36
COL6A1	21q22.3	0.381662378	7.59E-39	1.42E-36
CPZ	4p16.1	0.381589458	7.86E-39	1.44E-36
ASPHD2	22q12.1	0.381168625	9.64E-39	1.75E-36
FST	5q11.2	0.38080757	1.15E-38	2.05E-36
PDGFRL	8p22	0.380184474	1.55E-38	2.75E-36
F13A1	6p25.1	0.380122282	1.60E-38	2.80E-36
LTC4S	5q35.3	0.379824648	1.84E-38	3.21E-36
GNAI2	3p21.31	0.379191808	2.50E-38	4.28E-36
C1QTNF6	22q12.3	0.378333167	3.78E-38	6.30E-36
HPGDS	4q22.3	0.377722316	5.06E-38	8.31E-36
TMEM204	16p13.3	0.377605081	5.35E-38	8.65E-36
THPO	3q27.1	0.37752231	5.57E-38	8.92E-36
ARSI	5q32	0.377221724	6.43E-38	1.02E-35
CCN5	20q13.12	0.376517117	9.00E-38	1.41E-35
GGT5	22q11.23	0.375901009	1.21E-37	1.86E-35
TBC1D2	9q22.33	0.375527813	1.44E-37	2.20E-35
AZIN2	1p35.1	0.374713166	2.12E-37	3.15E-35
RASGRP4	19q13.2	0.374661562	2.17E-37	3.18E-35
CCDC157	22q12.2	0.373843083	3.20E-37	4.64E-35
CERCAM	9q34.11	0.373710775	3.40E-37	4.91E-35
IGSF21	1p36.13	0.372259365	6.74E-37	9.45E-35
TMEM173	5q31.2	0.372085344	7.31E-37	1.01E-34
SPI1	11p11.2	0.371827915	8.25E-37	1.13E-34
ARSA	22q13.33	0.37065396	1.43E-36	1.91E-34
P4HA3	11q13.4	0.37046315	1.56E-36	2.07E-34
GRP	18q21.32	0.370402787	1.61E-36	2.12E-34
TBC1D22A	22q13.31	0.370378932	1.62E-36	2.13E-34
TGFB1I1	16p11.2	0.370225308	1.75E-36	2.26E-34
PODN	1p32.3	0.369618441	2.31E-36	2.94E-34
ITGB5	3q21.2	0.369600475	2.33E-36	2.94E-34
SYDE1	19p13.12	0.369486517	2.46E-36	3.07E-34
PLD4	14q32.33	0.369357561	2.61E-36	3.22E-34
OLFML2B	1q23.3	0.369328308	2.65E-36	3.24E-34
MYL3	3p21.31	0.369158212	2.87E-36	3.49E-34
COL1A2	7q21.3	0.36845348	3.97E-36	4.78E-34
TSPAN15	10q22.1	0.368249814	4.37E-36	5.22E-34
TMEM240	1p36.33	0.368014862	4.87E-36	5.75E-34
TOR4A	9q34.3	0.367784089	5.42E-36	6.36E-34
PYCARD	16p11.2	0.367120164	7.36E-36	8.54E-34
LUM	12q21.33	0.366614692	9.29E-36	1.07E-33
LMCD1	3p25.3	0.366566453	9.50E-36	1.09E-33
CYBRD1	2q31.1	0.366249495	1.10E-35	1.25E-33
COL16A1	1p35.2	0.366177603	1.14E-35	1.28E-33

ID3	1p36.12	0.365671414	1.43E-35	1.61E-33
IGFBP6	12q13.13	0.365553193	1.51E-35	1.69E-33
MMP28	17q12	0.365027288	1.92E-35	2.13E-33
PLAAT4	11q12.3	0.3649687	1.98E-35	2.18E-33
ST6GALNAC6	9q34.11	0.364240956	2.76E-35	3.01E-33
PROC	2q14.3	0.363770208	3.42E-35	3.69E-33
LRRC15	3q29	0.363696394	3.53E-35	3.76E-33
CYTH4	22q13.1	0.36360507	3.68E-35	3.89E-33
PRR5L	11p13-p12	0.363584608	3.72E-35	3.91E-33
PLPP4	10q26.12	0.363423734	4.00E-35	4.18E-33
A4GALT	22q13.2	0.36323797	4.35E-35	4.51E-33
NFAM1	22q13.2	0.362630574	5.73E-35	5.82E-33
TMEM119	12q23.3	0.361926465	7.89E-35	7.81E-33
PLEKHO2	15q22.31	0.361417645	9.93E-35	9.78E-33
PDLIM2	8p21.3	0.36116551	1.11E-34	1.08E-32
RRAS	19q13.33	0.360815933	1.30E-34	1.25E-32
ASPN	9q22.31	0.360600273	1.44E-34	1.37E-32
CNRIP1	2p14	0.360597314	1.44E-34	1.37E-32
LSP1	11p15.5	0.360447128	1.54E-34	1.46E-32
MILR1	17q23.3	0.360324299	1.63E-34	1.53E-32
PLK3	1p34.1	0.360196671	1.72E-34	1.60E-32
SELPLG	12q24.11	0.359840725	2.02E-34	1.85E-32
LST1	6p21.33	0.359633668	2.22E-34	2.03E-32
HCFC1R1	16p13.3	0.358903313	3.08E-34	2.77E-32
INKA1	3p21.31	0.358675077	3.41E-34	3.04E-32
HLX	1q41	0.358247332	4.12E-34	3.64E-32
ABI3	17q21.32	0.358093444	4.42E-34	3.88E-32
COL5A1	9q34.3	0.357429898	5.94E-34	5.15E-32
LRRC25	19p13.11	0.35738823	6.05E-34	5.22E-32
OSCAR	19q13.42	0.357212689	6.54E-34	5.62E-32
NBL1	1p36.13	0.35697988	7.26E-34	6.21E-32
MRC2	17q23.2	0.356883845	7.57E-34	6.44E-32
PEBP4	8p21.3	0.356589415	8.63E-34	7.29E-32
B4GALT7	5q35.3	0.356435523	9.24E-34	7.77E-32
PMP22	17p12	0.356378807	9.48E-34	7.94E-32
TBXAS1	7q34	0.356137684	1.05E-33	8.72E-32
COMP	19p13.11	0.356036614	1.10E-33	9.09E-32
CALHM2	10q24.33	0.355980103	1.13E-33	9.28E-32
PLA2G5	1p36.13	0.355948009	1.15E-33	9.37E-32
UNC5B	10q22.1	0.355247109	1.56E-33	1.27E-31
TYMP	22q13.33	0.354873995	1.84E-33	1.49E-31
PARP3	3p21.2	0.354765091	1.93E-33	1.56E-31
TMEM91	19q13.2	0.354734388	1.96E-33	1.56E-31
ATP10A	15q12	0.354576129	2.10E-33	1.66E-31
SMIM3	5q33.1	0.354456934	2.22E-33	1.74E-31
ODF3B	22q13.33	0.354387211	2.29E-33	1.79E-31
AIF1	6p21.33	0.353984379	2.73E-33	2.11E-31
TNFRSF1A	12p13.31	0.353952874	2.77E-33	2.13E-31
IFI27L2	14q32.12	0.353901584	2.83E-33	2.17E-31
ELFN1	7p22.3	0.353830384	2.92E-33	2.22E-31
PDPN	1p36.21	0.353798756	2.96E-33	2.25E-31
IFITM2	11p15.5	0.353151879	3.93E-33	2.94E-31
HIC1	17p13.3	0.352999561	4.21E-33	3.13E-31
PPP1R3G	6p25.1	0.352901069	4.39E-33	3.25E-31
KCTD11	17p13.1	0.352299284	5.71E-33	4.21E-31
TNFAIP8L2	1q21.3	0.352155262	6.08E-33	4.45E-31
SLC1A7	1p32.3	0.352109808	6.21E-33	4.52E-31
DENND6B	22q13.33	0.351594611	7.77E-33	5.60E-31
ITPRIPL2	16p12.3	0.351578197	7.83E-33	5.62E-31
MIR22HG	17p13.3	0.350728754	1.13E-32	8.08E-31
RNF215	22q12.2	0.350360899	1.33E-32	9.38E-31
SERPING1	11q12.1	0.350195168	1.43E-32	1.00E-30
GLT8D2	12q23.3	0.350175818	1.44E-32	1.01E-30
TLL2	10q24.1	0.348890616	2.51E-32	1.72E-30
IGFBP7	4q12	0.348600258	2.85E-32	1.94E-30
RGS14	5q35.3	0.348510182	2.96E-32	2.01E-30

VWA1	1p36.33	0.347991557	3.70E-32	2.49E-30
FIBIN	11p14.2	0.347592443	4.40E-32	2.93E-30
SLC49A3	4p16.3	0.347343997	4.89E-32	3.22E-30
SLC39A13	11p11.2	0.347246869	5.10E-32	3.33E-30
SFRP4	7p14.1	0.347045817	5.56E-32	3.62E-30
C5ORF38	5p15.33	0.346781433	6.23E-32	4.04E-30
CYP21A2	6p21.33	0.34621232	7.94E-32	5.11E-30
SORCS2	4p16.1	0.34619913	7.99E-32	5.12E-30
PRAM1	19p13.2	0.345297497	1.17E-31	7.40E-30
SLC8B1	12q24.13	0.34465998	1.54E-31	9.59E-30
CHPF	2q35	0.344651268	1.55E-31	9.60E-30
SPARC	5q33.1	0.344491654	1.65E-31	1.02E-29
MSX1	4p16.2	0.344176635	1.89E-31	1.16E-29
ZBTB47	3p22.1	0.343906205	2.12E-31	1.30E-29
SSC5D	19q13.42	0.343751412	2.27E-31	1.38E-29
SFRP2	4q31.3	0.343493012	2.53E-31	1.54E-29
EVA1B	1p34.3	0.343377142	2.65E-31	1.61E-29
ZMAT5	22q12.2	0.342491361	3.86E-31	2.30E-29
CRYBB1	22q12.1	0.341988714	4.77E-31	2.82E-29
BORCS6	17p13.1	0.341379402	6.16E-31	3.62E-29
SPEF1	20p13	0.340969003	7.32E-31	4.27E-29
EHD2	19q13.33	0.340424525	9.20E-31	5.35E-29
DKK3	11p15.3	0.340390596	9.33E-31	5.41E-29
HSCB	22q12.1	0.340288773	9.74E-31	5.60E-29
OMD	9q22.31	0.340154055	1.03E-30	5.89E-29
PTRH1	9q34.11	0.340096782	1.06E-30	6.00E-29
CD300C	17q25.1	0.339908581	1.14E-30	6.47E-29
RAB20	13q34	0.33988027	1.16E-30	6.53E-29
SH3TC1	4p16.1	0.339644485	1.27E-30	7.13E-29
SLC38A5	Xp11.23	0.339539375	1.33E-30	7.43E-29
PKIG	20q13.12	0.339225815	1.52E-30	8.40E-29
PDE4A	19p13.2	0.339175933	1.55E-30	8.55E-29
RIN2	20p11.23	0.338955832	1.70E-30	9.34E-29
TMEM255B	13q34	0.338899973	1.74E-30	9.54E-29
THBD	20p11.21	0.338533101	2.03E-30	1.11E-28
SLC15A3	11q12.2	0.338290879	2.24E-30	1.22E-28
CD33	19q13.41	0.338012871	2.51E-30	1.36E-28
APOL2	22q12.3	0.33794402	2.59E-30	1.40E-28
MMP11	22q11.23	0.337844914	2.70E-30	1.45E-28
SNED1	2q37.3	0.337733339	2.82E-30	1.51E-28
PRSS36	16p11.2	0.337644052	2.93E-30	1.55E-28
CAVIN1	17q21.2	0.337502058	3.11E-30	1.64E-28
INAFM1	19q13.32	0.337217194	3.50E-30	1.83E-28
AXL	19q13.2	0.337163844	3.57E-30	1.87E-28
RAB3IL1	11q12.2-q12.3	0.337162149	3.58E-30	1.87E-28
COL5A3	19p13.2	0.337119631	3.64E-30	1.89E-28
LGALS1	22q13.1	0.336604424	4.50E-30	2.32E-28
KLKP1	19q13.33	0.336283272	5.14E-30	2.63E-28
MYO1F	19p13.2	0.335876564	6.08E-30	3.08E-28
P3H3	12p13.31	0.335654918	6.66E-30	3.36E-28
CSF1	1p13.3	0.33521488	7.98E-30	4.01E-28
S100A4	1q21.3	0.335111024	8.33E-30	4.17E-28
PIK3IP1	22q12.2	0.335073686	8.46E-30	4.23E-28
NINJ1	9q22.31	0.335012817	8.67E-30	4.32E-28
MYL9	20q11.23	0.334880965	9.16E-30	4.55E-28
ITGBL1	13q33.1	0.334685859	9.92E-30	4.92E-28
NTN4	12q22	0.334323009	1.15E-29	5.69E-28
LRP1	12q13.3	0.334000274	1.31E-29	6.45E-28
DEGS2	14q32.2	0.333993936	1.32E-29	6.45E-28
CYB5A	18q22.3	0.333865749	1.39E-29	6.77E-28
ISLR	15q24.1	0.333629173	1.53E-29	7.43E-28
NCF4	22q12.3	0.333508612	1.61E-29	7.79E-28
CRTAP	3p22.3	0.333399183	1.68E-29	8.13E-28
RILPL2	12q24.31	0.332907898	2.05E-29	9.86E-28
MEG3	14q32.2	0.332542749	2.38E-29	1.14E-27
TRIM8	10q24.32	0.332409363	2.51E-29	1.20E-27

CAVIN3	11p15.4	0.332229143	2.70E-29	1.28E-27
BGN	Xq28	0.332021772	2.94E-29	1.39E-27
KCNE4	2q36.1	0.332010451	2.95E-29	1.40E-27
MITF	3p13	0.331896935	3.09E-29	1.45E-27
FAM20C	7p22.3	0.331843316	3.16E-29	1.48E-27
LIMS2	2q14.3	0.33169037	3.36E-29	1.57E-27
FSTL3	19p13.3	0.33141536	3.76E-29	1.75E-27
KLK4	19q13.41	0.331337901	3.88E-29	1.81E-27
APOL1	22q12.3	0.330882703	4.67E-29	2.15E-27
GLIS1	1p32.3	0.330646248	5.13E-29	2.35E-27
CSF1R	5q32	0.330634972	5.16E-29	2.36E-27
LIPC	15q21.3	0.330459791	5.54E-29	2.52E-27
UBA7	3p21.31	0.330430568	5.60E-29	2.55E-27
CTSD	11p15.5	0.33023842	6.05E-29	2.74E-27
ITGA11	15q23	0.330002109	6.66E-29	3.00E-27
CD74	5q33.1	0.329752884	7.36E-29	3.31E-27
NUDT18	8p21.3	0.32959108	7.86E-29	3.52E-27
NOG	17q22	0.329409354	8.45E-29	3.77E-27
ALOX5	10q11.21	0.329373871	8.57E-29	3.81E-27
FGF14	13q33.1	0.329234092	9.07E-29	4.02E-27
PHLDB1	11q23.3	0.329000127	9.96E-29	4.41E-27
SYNC	1p35.1	0.328868869	1.05E-28	4.63E-27
ARHGEF40	14q11.2	0.328770382	1.09E-28	4.80E-27
LINC00922	16q21	0.328509538	1.21E-28	5.32E-27
NUPR1	16p11.2	0.328345081	1.30E-28	5.67E-27
MMP14	14q11.2	0.327764345	1.63E-28	7.11E-27
C1R	12p13.31	0.327731831	1.66E-28	7.19E-27
NOX4	11q14.3	0.327708034	1.67E-28	7.24E-27
SERPINA1	14q32.13	0.327660222	1.70E-28	7.36E-27
CCDC159	19p13.2	0.327544772	1.78E-28	7.69E-27
TANGO2	22q11.21	0.32739483	1.89E-28	8.15E-27
GALNT15	3p25.1	0.326820654	2.38E-28	1.02E-26
MIAT	22q12.1	0.326484211	2.72E-28	1.16E-26
PHPT1	9q34.3	0.326401964	2.81E-28	1.19E-26
LRRN4CL	11q12.3	0.326041189	3.25E-28	1.36E-26
SMOC2	6q27	0.325741222	3.66E-28	1.53E-26
CAMK2A	5q32	0.325633918	3.82E-28	1.60E-26
TPM1	15q22.2	0.325516281	4.00E-28	1.66E-26
STX4	16p11.2	0.325427013	4.14E-28	1.72E-26
CSDC2	22q13.2	0.325344408	4.28E-28	1.77E-26
ZNF385D	3p24.3	0.3253245	4.31E-28	1.78E-26
RNH1	11p15.5	0.325169767	4.59E-28	1.89E-26
SLIT3	5q34-q35.1	0.325133598	4.65E-28	1.92E-26
TWIST2	2q37.3	0.325103174	4.71E-28	1.94E-26
PRDM6	5q23.2	0.32504347	4.82E-28	1.98E-26
ZCCHC24	10q22.3	0.324840154	5.22E-28	2.13E-26
RAB31	18p11.22	0.324400032	6.22E-28	2.52E-26
SH3RF3	2q13	0.324396769	6.22E-28	2.52E-26
CXCL12	10q11.21	0.32374656	8.04E-28	3.24E-26
HHIPL1	14q32.2	0.323735051	8.08E-28	3.25E-26
SFI1	22q12.2	0.323634155	8.40E-28	3.36E-26
FERMT3	11q13.1	0.323606536	8.50E-28	3.39E-26
SLC46A2	9q32	0.323567024	8.63E-28	3.44E-26
TMEM218	11q24.2	0.323434207	9.09E-28	3.61E-26
HLA-DPB1	6p21.32	0.323134071	1.02E-27	4.04E-26
GRAP	17p11.2	0.323003577	1.08E-27	4.25E-26
TBC1D10A	22q12.2	0.322605969	1.26E-27	4.92E-26
FCGR1CP	1q21.1	0.321812811	1.72E-27	6.68E-26
CD300A	17q25.1	0.32176084	1.75E-27	6.80E-26
TWF2	3p21.2	0.321597257	1.87E-27	7.23E-26
GPX1	3p21.31	0.321428631	1.99E-27	7.70E-26
PTGS1	9q33.2	0.321422474	2.00E-27	7.70E-26
ADAP2	17q11.2	0.321251486	2.14E-27	8.22E-26
CYP4X1	1p33|1	0.321183956	2.19E-27	8.40E-26
SIL1	5q31.2	0.321141128	2.23E-27	8.53E-26
DPT	1q24.2	0.320989425	2.37E-27	9.03E-26

ADAM33	20p13	0.320919532	2.43E-27	9.26E-26
PLAC9	10q22.3	0.32075878	2.59E-27	9.84E-26
LAYN	11q23.1	0.3207499	2.60E-27	9.86E-26
ITGAM	16p11.2	0.320705505	2.64E-27	1.00E-25
VAMP5	2p11.2	0.320644554	2.71E-27	1.02E-25
TPRN	9q34.3	0.320463731	2.90E-27	1.09E-25
OLFML1	11p15.4	0.320181264	3.24E-27	1.22E-25
OAF	11q23.3	0.320143834	3.29E-27	1.23E-25
RCN3	19q13.33	0.320023101	3.45E-27	1.29E-25
SAMD14	17q21.33	0.319915739	3.59E-27	1.34E-25
CCDC71	3p21.31	0.319843971	3.69E-27	1.38E-25
PLAU	10q22.2	0.319594275	4.07E-27	1.50E-25
MS4A7	11q12.2	0.319057746	5.01E-27	1.85E-25
STAB1	3p21.1	0.3190424	5.04E-27	1.85E-25
DPEP2	16q22.1	0.319037814	5.05E-27	1.85E-25
GPC1	2q37.3	0.319022185	5.08E-27	1.86E-25
KCNK13	14q32.11	0.318843244	5.44E-27	1.98E-25
LY96	8q21.11	0.318400899	6.46E-27	2.34E-25
HOXA11-AS	7p15.2	0.318303003	6.70E-27	2.43E-25
PLIN3	19p13.3	0.318255096	6.83E-27	2.47E-25
MAF	16q23.2	0.318226094	6.91E-27	2.49E-25
HOXA11	7p15.2	0.318224097	6.91E-27	2.49E-25
MIR4435-2HG	2q13	0.318166067	7.07E-27	2.53E-25
ARHGDIB	12p12.3	0.318015573	7.49E-27	2.67E-25
PDZRN4	12q12	0.317966409	7.63E-27	2.72E-25
GPR162	12p13.31	0.317962671	7.64E-27	2.72E-25
OTOA	16p12.2|16p12.2	0.317940313	7.71E-27	2.74E-25
CEBPE	14q11.2	0.317755862	8.28E-27	2.91E-25
FCGR1B	1p11.2	0.317711394	8.42E-27	2.96E-25
COL3A1	2q32.2	0.317610109	8.76E-27	3.07E-25
RFX8	2q11.2	0.317189743	1.03E-26	3.58E-25
FCGBP	19q13.2	0.316938548	1.13E-26	3.93E-25
RASL10A	22q12.2	0.316568614	1.31E-26	4.50E-25
SNAI3	16q24.2	0.316346186	1.42E-26	4.87E-25
IGFL2	19q13.32	0.316335213	1.43E-26	4.88E-25
TAGLN	11q23.3	0.316233411	1.48E-26	5.07E-25
GLRX	5q15	0.316160377	1.53E-26	5.21E-25
MMP19	12q13.2	0.31614178	1.54E-26	5.23E-25
MAFB	20q12	0.315997754	1.62E-26	5.50E-25
CATSPER1	11q13.1	0.315963126	1.65E-26	5.57E-25
NHLRC4	16p13.3	0.315862603	1.71E-26	5.77E-25
BMERB1	16p13.11	0.315733369	1.80E-26	6.06E-25
TXNDC15	5q31.1	0.315653169	1.85E-26	6.23E-25
OXT	20p13	0.315582641	1.90E-26	6.39E-25
CERKL	2q31.3	0.315454087	2.00E-26	6.70E-25
VEGFB	11q13.1	0.315200484	2.20E-26	7.36E-25
TSPAN9	12p13.33-p13.32	0.314978484	2.40E-26	8.00E-25
TNFRSF14	1p36.32	0.314929553	2.44E-26	8.13E-25
BMP1	8p21.3	0.314864073	2.50E-26	8.32E-25
SLC27A1	19p13.11	0.314730812	2.63E-26	8.74E-25
PHYHD1	9q34.11	0.31466431	2.70E-26	8.95E-25
RILP	17p13.3	0.314606893	2.76E-26	9.14E-25
FPR1	19q13.41	0.314500736	2.87E-26	9.50E-25
HOXB6	17q21.32	0.314475716	2.90E-26	9.57E-25
TM6SF2	19p13.11	0.314395572	2.99E-26	9.83E-25
SALL1	16q12.1	0.31438729	3.00E-26	9.85E-25
ENOX1	13q14.11	0.314292645	3.11E-26	1.02E-24
CPXM1	20p13	0.314157907	3.27E-26	1.07E-24
MMP17	12q24.33	0.314018404	3.45E-26	1.12E-24
C8ORF58	8p21.3	0.313948421	3.55E-26	1.15E-24
CD99	Xp22.33 and Yp11.2	0.313892345	3.62E-26	1.17E-24
HSPA12B	20p13	0.313796375	3.76E-26	1.21E-24
HS3ST3A1	17p12	0.313353737	4.44E-26	1.43E-24
GNG11	7q21.3	0.313151529	4.79E-26	1.54E-24
PRR34	22q13.31	0.313091932	4.90E-26	1.57E-24
LAMB2	3p21.31	0.312887778	5.30E-26	1.69E-24

FTH1P3	2p23.3	0.312871645	5.33E-26	1.70E-24
RASGRF2	5q14.1	0.312709398	5.67E-26	1.80E-24
ADAMTS14	10q22.1	0.312673281	5.74E-26	1.81E-24
CFB	6p21.33	0.312337192	6.52E-26	2.05E-24
NKX3-2	4p15.33	0.312280557	6.66E-26	2.09E-24
SIGLEC9	19q13.41	0.312254468	6.73E-26	2.11E-24
MFAP5	12p13.31	0.312230476	6.79E-26	2.13E-24
NPTX2	7q22.1	0.312123335	7.07E-26	2.20E-24
SHROOM1	5q31.1	0.31205379	7.25E-26	2.26E-24
KCTD17	22q12.3	0.312052315	7.26E-26	2.26E-24
SRPX2	Xq22.1	0.311993871	7.42E-26	2.30E-24
CHKB	22q13.33	0.311824747	7.91E-26	2.45E-24
CCDC80	3q13.2	0.311424965	9.19E-26	2.84E-24
SLC40A1	2q32.2	0.311127787	1.03E-25	3.16E-24
EGLN3	14q13.1	0.310888512	1.12E-25	3.45E-24
RRAD	16q22.1	0.310674015	1.22E-25	3.73E-24
MS4A14	11q12.2	0.310340386	1.38E-25	4.22E-24
SERTAD1	19q13.2	0.310266261	1.42E-25	4.33E-24
C1QTNF7	4p15.32	0.310173867	1.47E-25	4.48E-24
ANGPTL2	9q33.3	0.310021709	1.55E-25	4.72E-24
CDC42EP5	19q13.42	0.309990172	1.57E-25	4.77E-24
MMP23B	1p36.33	0.309834024	1.67E-25	5.05E-24
FGF18	5q35.1	0.309729163	1.73E-25	5.24E-24
TNFSF13	17p13.1	0.309660781	1.78E-25	5.37E-24
PIP	7q34	0.309652722	1.78E-25	5.37E-24
C10ORF55	10q22.2	0.309627093	1.80E-25	5.42E-24
JUNB	19p13.13	0.309557635	1.85E-25	5.54E-24
ADORA3	1p13.2	0.309525092	1.87E-25	5.60E-24
ZNF688	16p11.2	0.309524831	1.87E-25	5.60E-24
VOPP1	7p11.2	0.308970412	2.30E-25	6.85E-24
HLA-DMA	6p21.32	0.30882713	2.43E-25	7.18E-24
C16ORF86	16q22.1	0.308814361	2.44E-25	7.20E-24
RNASE6	14q11.2	0.308622455	2.62E-25	7.71E-24
NUDT22	11q13.1	0.308512651	2.73E-25	8.00E-24
TIMP1	Xp11.3	0.308299421	2.95E-25	8.65E-24
ZNF668	16p11.2	0.308287883	2.96E-25	8.67E-24
ADRA2A	10q25.2	0.308264606	2.99E-25	8.73E-24
DLG4	17p13.1	0.30819378	3.07E-25	8.95E-24
TCTEX1D4	1p34.1	0.308000781	3.30E-25	9.60E-24
DACT1	14q23.1	0.307925131	3.39E-25	9.82E-24
LGALS3	14q22.3	0.307756432	3.61E-25	1.04E-23
HOXB5	17q21.32	0.307486524	3.99E-25	1.14E-23
RGCC	13q14.11	0.307360913	4.18E-25	1.19E-23
SHISAL1	22q13.31	0.307330313	4.23E-25	1.20E-23
MPG	16p13.3	0.307308183	4.26E-25	1.21E-23
MN1	22q12.1	0.307143133	4.53E-25	1.28E-23
HLA-DRB1	6p21.32	0.307125635	4.56E-25	1.29E-23
ACKR4	3q22.1	0.307096722	4.61E-25	1.30E-23
TIMP2	17q25.3	0.307040157	4.70E-25	1.33E-23
HABP2	10q25.3	0.306996735	4.78E-25	1.34E-23
POLD4	11q13.2	0.306553285	5.63E-25	1.58E-23
IL4R	16p12.1	0.306335252	6.10E-25	1.70E-23
TMSB4XP8	4q22.1	0.306220546	6.36E-25	1.77E-23
ITGA5	12q13.13	0.306210882	6.39E-25	1.77E-23
COL18A1	21q22.3	0.306202427	6.41E-25	1.77E-23
SHISAL2A	1p32.3	0.306127342	6.59E-25	1.82E-23
CD248	11q13.2	0.305859993	7.27E-25	2.00E-23
FAP	2q24.2	0.305832193	7.34E-25	2.02E-23
REEP6	19p13.3	0.305717596	7.66E-25	2.10E-23
CACNA2D4	12p13.33	0.305298238	8.93E-25	2.44E-23
NTM	11q25	0.305176927	9.34E-25	2.55E-23
SLC37A2	11q24.2	0.305134231	9.49E-25	2.58E-23
EMX2	10q26.11	0.305047988	9.79E-25	2.66E-23
RTN1	14q23.1	0.304903799	1.03E-24	2.80E-23
CNN2	19p13.3	0.304848649	1.05E-24	2.86E-23
CLEC5A	7q34	0.304601518	1.15E-24	3.11E-23

PARVG	22q13.31	0.304283626	1.30E-24	3.47E-23
NEK10	3p24.1	0.303655674	1.63E-24	4.31E-23
LAPTM5	1p35.2	0.303648979	1.63E-24	4.32E-23
CFD	19p13.3	0.303588506	1.67E-24	4.41E-23
DRD4	11p15.5	0.303506782	1.72E-24	4.54E-23
VMO1	17p13.2	0.303302543	1.85E-24	4.87E-23
RFTN1	3p24.3	0.303164052	1.95E-24	5.12E-23
ADAMTSL5	19p13.3	0.302856042	2.18E-24	5.70E-23
ACOX2	3p14.3	0.302820362	2.21E-24	5.76E-23
ENG	9q34.11	0.302612265	2.38E-24	6.20E-23
UBXN6	19p13.3	0.302574721	2.41E-24	6.27E-23
HOXD9	2q31.1	0.30242815	2.54E-24	6.60E-23
COL10A1	6q22.1	0.302293923	2.67E-24	6.92E-23
STARD5	15q25.1	0.302147575	2.82E-24	7.28E-23
MOB3C	1p33	0.302009275	2.96E-24	7.64E-23
MOB3A	19p13.3	0.301961365	3.01E-24	7.76E-23
CYSTM1	5q31.3	0.301235725	3.92E-24	1.00E-22
PDLIM4	5q31.1	0.30118969	3.98E-24	1.02E-22
SLC12A4	16q22.1	0.30093348	4.37E-24	1.11E-22
SNX21	20q13.12	0.300865358	4.48E-24	1.14E-22
RASAL3	19p13.12	0.300450126	5.20E-24	1.31E-22
TREML1	6p21.1	0.300441727	5.22E-24	1.31E-22
ABCD4	14q24.3	0.30037163	5.35E-24	1.34E-22

Supplementary Table S4. The 323 genes, of which expressions were negatively correlated with TPST2 expression.

Correlated Gene	Cytoband	Spearman's Correlation	p-Value	q-Value
TMPO	12q23.1	-0.429043205	1.10E-49	1.17E-46
U2SURP	3q23	-0.413642028	5.73E-46	3.61E-43
MSH2	2p21-p16.3	-0.409284873	5.95E-45	2.94E-42
ATL2	2p22.2-p22.1	-0.405275071	4.98E-44	2.01E-41
MAD2L1	4q27	-0.402256266	2.42E-43	8.87E-41
TOP2A	17q21.2	-0.399006476	1.30E-42	4.38E-40
KNTC1	12q24.31	-0.397649694	2.61E-42	8.37E-40
CPSF6	12q15	-0.397106825	3.45E-42	1.06E-39
ARHGAP11A	15q13.3	-0.396627165	4.41E-42	1.33E-39
PPAT	4q12	-0.396117782	5.72E-42	1.67E-39
MTMR4	17q22	-0.395430357	8.12E-42	2.31E-39
ATAD5	17q11.2	-0.395000998	1.01E-41	2.79E-39
PARPBP	12q23.2	-0.394564687	1.26E-41	3.39E-39
MBTD1	17q21.33	-0.39442551	1.35E-41	3.59E-39
XRCC2	7q36.1	-0.391283175	6.59E-41	1.64E-38
BRIP1	17q23.2	-0.391097415	7.24E-41	1.78E-38
NOL11	17q24.2	-0.38939517	1.69E-40	4.02E-38
CKAP5	11p11.2	-0.38646908	7.23E-40	1.59E-37
RFWD3	16q23.1	-0.384448967	1.95E-39	3.98E-37
NEMP1	12q13.3	-0.383478382	3.14E-39	6.27E-37
BLM	15q26.1	-0.382794777	4.38E-39	8.46E-37
CIP2A	3q13.13	-0.381588684	7.86E-39	1.44E-36
CENPE	4q24	-0.380904206	1.10E-38	1.97E-36
CASP2	7q34	-0.379224312	2.46E-38	4.25E-36
LARP4B	10p15.3	-0.379082195	2.64E-38	4.47E-36
MMS22L	6q16.1	-0.378435193	3.60E-38	6.05E-36
CIT	12q24.23	-0.377827459	4.81E-38	7.97E-36
BUB1B	15q15.1	-0.377618474	5.32E-38	8.65E-36
TOPBP1	3q22.1	-0.376763436	8.00E-38	1.26E-35
NAA25	12q24.13	-0.376134332	1.08E-37	1.68E-35
PLK4	4q28.1	-0.375519738	1.45E-37	2.20E-35
NUP205	7q33	-0.375413445	1.52E-37	2.29E-35
ZGRF1	4q25	-0.375162065	1.71E-37	2.56E-35
UBA2	19q13.11	-0.374670769	2.16E-37	3.18E-35
PRR11	17q22	-0.373183702	4.36E-37	6.25E-35
MTBP	8q24.12	-0.372628472	5.67E-37	8.06E-35
STIL	1p33	-0.372570389	5.82E-37	8.22E-35
TDG	12q23.3	-0.372237724	6.81E-37	9.48E-35
RAD51AP1	12p13.32	-0.37174888	8.56E-37	1.17E-34
BUB1	2q13	-0.371421587	9.98E-37	1.35E-34
SASS6	1p21.2	-0.370719145	1.39E-36	1.87E-34
WDHD1	14q22.2-q22.3	-0.370274659	1.71E-36	2.22E-34
KIF20B	10q23.31	-0.370202453	1.76E-36	2.27E-34
DCAF7	17q23.3	-0.369729823	2.20E-36	2.81E-34
ASPM	1q31.3	-0.369588917	2.35E-36	2.94E-34
SGO1	3p24.3	-0.369360129	2.61E-36	3.22E-34
RRP1B	21q22.3	-0.368538048	3.82E-36	4.62E-34
KIF11	10q23.33	-0.368031714	4.83E-36	5.74E-34
ARHGAP11B	15q13.2	-0.367237834	6.97E-36	8.13E-34
KIF18A	11p14.1	-0.366257572	1.09E-35	1.25E-33
ENOPH1	4q21.22	-0.364853962	2.08E-35	2.29E-33
ATAD2	8q24.13	-0.364076669	2.97E-35	3.22E-33
TTK	6q14.1	-0.363707031	3.52E-35	3.76E-33
WDR43	2p23.2	-0.363694328	3.54E-35	3.76E-33
LRPPRC	2p21	-0.363314573	4.20E-35	4.37E-33
XK	Xp21.1	-0.363205148	4.42E-35	4.55E-33
FANCI	15q26.1	-0.362950091	4.96E-35	5.08E-33
CABLES2	20q13.33	-0.362730632	5.48E-35	5.59E-33
DNA2	10q21.3	-0.36261533	5.77E-35	5.83E-33
POLQ	3q13.33	-0.362589423	5.84E-35	5.87E-33
BTBD3	20p12.2	-0.362373157	6.44E-35	6.44E-33
DEPDC1	1p31.3	-0.362292758	6.68E-35	6.65E-33
TICRR	15q26.1	-0.361386654	1.01E-34	9.87E-33
PRC1	15q26.1	-0.361225774	1.08E-34	1.06E-32
NKRF	Xq24	-0.360982691	1.21E-34	1.17E-32
RAVER2	1p31.3	-0.360292824	1.65E-34	1.55E-32

TET3	2p13.1	-0.360241891	1.69E-34	1.58E-32
SRSF1	17q22	-0.360187144	1.73E-34	1.60E-32
DBF4	7q21.12	-0.36011754	1.78E-34	1.64E-32
CKAP2L	2q14.1	-0.359326687	2.54E-34	2.31E-32
GTF3C2	2p23.3	-0.35924792	2.64E-34	2.39E-32
MTPAP	10p11.23	-0.358807562	3.21E-34	2.88E-32
CCNA2	4q27	-0.358656198	3.44E-34	3.06E-32
TLK2	17q23.2	-0.358579868	3.56E-34	3.15E-32
SELENOI	2p23.3	-0.357833336	4.96E-34	4.34E-32
E2F7	12q21.2	-0.357674612	5.33E-34	4.63E-32
SRSF10	1p36.11	-0.356876527	7.60E-34	6.44E-32
CEP152	15q21.1	-0.356303707	9.80E-34	8.17E-32
RACGAP1	12q13.12	-0.356176143	1.04E-33	8.61E-32
ZBTB39	12q13.3	-0.355489351	1.41E-33	1.14E-31
ZDHHC13	11p15.1	-0.354754752	1.94E-33	1.56E-31
XPO5	6p21.1	-0.354635498	2.05E-33	1.63E-31
RAD54B	8q22.1	-0.354592067	2.09E-33	1.65E-31
ESPL1	12q13.13	-0.354312325	2.36E-33	1.84E-31
CENPF	1q41	-0.354310089	2.36E-33	1.84E-31
DIAPH3	13q21.2	-0.353875477	2.86E-33	2.19E-31
GEN1	2p24.2	-0.353534851	3.32E-33	2.51E-31
NCAPG2	7q36.3	-0.353519817	3.35E-33	2.52E-31
NEIL3	4q34.3	-0.353199064	3.85E-33	2.89E-31
ZFP69B	1p34.2	-0.352923062	4.35E-33	3.23E-31
THOC2	Xq25	-0.352156996	6.08E-33	4.45E-31
BAZ1B	7q11.23	-0.351713576	7.38E-33	5.36E-31
NSD2	4p16.3	-0.351598383	7.76E-33	5.60E-31
SF3B3	16q22.1	-0.351555965	7.90E-33	5.66E-31
NUP153	6p22.3	-0.350534661	1.23E-32	8.76E-31
TRIM24	7q33-q34	-0.350527574	1.24E-32	8.76E-31
MCM10	10p13	-0.349951637	1.59E-32	1.11E-30
NCAPG	4p15.31	-0.349930206	1.60E-32	1.12E-30
CCNJ	10q24.1	-0.349040356	2.36E-32	1.63E-30
XPO1	2p15	-0.349014216	2.38E-32	1.65E-30
DTL	1q32.3	-0.348937814	2.46E-32	1.70E-30
SPIN4	Xq11.1	-0.34889228	2.51E-32	1.72E-30
FAM72B	1p11.2	-0.348668541	2.77E-32	1.89E-30
FBXO45	3q29	-0.348305498	3.23E-32	2.18E-30
SKA1	18q21.1	-0.347866135	3.91E-32	2.62E-30
PSMD12	17q24.2	-0.347639024	4.31E-32	2.88E-30
KIF15	3p21.31	-0.347585708	4.41E-32	2.93E-30
E2F8	11p15.1	-0.347456445	4.66E-32	3.08E-30
TCERG1	5q32	-0.347451704	4.67E-32	3.08E-30
CENPI	Xq22.1	-0.347248633	5.10E-32	3.33E-30
CDC25A	3p21.31	-0.346600364	6.73E-32	4.35E-30
FIGNL1	7p12.2	-0.346309542	7.62E-32	4.91E-30
KIF23	15q23	-0.345999816	8.70E-32	5.56E-30
KIF18B	17q21.31	-0.345764671	9.62E-32	6.12E-30
DHTKD1	10p14	-0.345569206	1.05E-31	6.64E-30
METAP1	4q23	-0.345461727	1.09E-31	6.93E-30
LIN9	1q42.12	-0.345291729	1.18E-31	7.40E-30
POLA1	Xp22.11-p21.3	-0.344815951	1.44E-31	9.03E-30
DEPDC1B	5q12.1	-0.34475798	1.48E-31	9.23E-30
MKI67	10q26.2	-0.344591387	1.59E-31	9.82E-30
C4ORF46	4q32.1	-0.343974105	2.06E-31	1.26E-29
KPNA2	17q24.2	-0.343259247	2.79E-31	1.69E-29
BEND3	6q21	-0.343055724	3.04E-31	1.83E-29
TPX2	20q11.21	-0.342924216	3.21E-31	1.93E-29
LIN54	4q21.22	-0.342794452	3.40E-31	2.03E-29
GAS2L3	12q23.1	-0.342307435	4.17E-31	2.48E-29
NUSAP1	15q15.1	-0.342268192	4.24E-31	2.52E-29
ABCE1	4q31.21	-0.342011333	4.72E-31	2.80E-29
HSPA14	10p13	-0.341926856	4.90E-31	2.88E-29
ZNF507	19q13.11	-0.341121321	6.87E-31	4.02E-29
TMEM209	7q32.2	-0.34038523	9.35E-31	5.41E-29
ASAP2	2p25.1|2p24	-0.340354668	9.47E-31	5.46E-29
IQGAP3	1q22	-0.340274637	9.80E-31	5.62E-29
GPSM2	1p13.3	-0.34012531	1.04E-30	5.95E-29

RMND5A	2p11.2	-0.339872716	1.16E-30	6.53E-29
ZNF473	19q13.33	-0.339774331	1.21E-30	6.79E-29
QTRT2	3q13.31	-0.339768074	1.21E-30	6.79E-29
RFC3	13q13.2	-0.339327841	1.45E-30	8.09E-29
BPTF	17q24.2	-0.339296172	1.47E-30	8.18E-29
PRELID3B	20q13.32	-0.33879079	1.82E-30	9.95E-29
KIF14	1q32.1	-0.338317915	2.22E-30	1.21E-28
FAM72A	1q32.1	-0.337811924	2.73E-30	1.47E-28
FOXM1	12p13.33	-0.337803646	2.74E-30	1.47E-28
SGO2	2q33.1	-0.337703886	2.86E-30	1.52E-28
NCAPH	2q11.2	-0.337694024	2.87E-30	1.52E-28
CDCA8	1p34.3	-0.337466753	3.15E-30	1.66E-28
KIF4A	Xq13.1	-0.337391563	3.25E-30	1.71E-28
DKC1	Xq28	-0.336880089	4.02E-30	2.09E-28
SS18L1	20q13.33	-0.336856691	4.06E-30	2.10E-28
KNSTRN	15q15.1	-0.33683038	4.10E-30	2.12E-28
GINS3	16q21	-0.33649472	4.71E-30	2.42E-28
ANLN	7p14.2	-0.335971846	5.85E-30	2.99E-28
TTC21B	2q24.3	-0.335961846	5.87E-30	2.99E-28
KNL1	15q15.1	-0.335946189	5.91E-30	3.00E-28
PAICS	4q12	-0.335741718	6.43E-30	3.25E-28
CLSPN	1p34.3	-0.335381988	7.45E-30	3.75E-28
POLR1B	2q14.1	-0.334305464	1.16E-29	5.72E-28
MELK	9p13.2	-0.334265468	1.18E-29	5.80E-28
CENPO	2p23.3	-0.333910667	1.36E-29	6.66E-28
SPAG5	17q11.2	-0.333303989	1.75E-29	8.43E-28
ANKIB1	7q21.2	-0.332924728	2.04E-29	9.81E-28
CKAP2	13q14.3	-0.332593787	2.33E-29	1.12E-27
CEP76	18p11.21	-0.332256629	2.67E-29	1.27E-27
DSCC1	8q24.12	-0.332002068	2.96E-29	1.40E-27
RBMX	Xq26.3	-0.331930544	3.05E-29	1.44E-27
UBA6	4q13.2	-0.331184326	4.13E-29	1.92E-27
ATF7IP	12p13.1	-0.33107958	4.31E-29	1.99E-27
NAA15	4q31.1	-0.331070069	4.33E-29	2.00E-27
FAM72D	1q21.1	-0.330888532	4.66E-29	2.15E-27
ECT2	3q26.31	-0.330723686	4.98E-29	2.28E-27
TRIP13	5p15.33	-0.330329155	5.83E-29	2.65E-27
GINS1	20p11.21	-0.330196047	6.16E-29	2.78E-27
SCML2	Xp22.13	-0.32974735	7.38E-29	3.31E-27
TAF4B	18q11.2	-0.329537844	8.03E-29	3.58E-27
C18ORF54	18q21.2	-0.328911366	1.03E-28	4.56E-27
HSPA4L	4q28.1	-0.328288745	1.32E-28	5.79E-27
HJURP	2q37.1	-0.328050076	1.46E-28	6.35E-27
TTF2	1p13.1	-0.327029001	2.19E-28	9.41E-27
TTI1	20q11.23	-0.326834305	2.37E-28	1.01E-26
SKA3	13q12.11	-0.326617605	2.58E-28	1.10E-26
RFC5	12q24.23	-0.326611931	2.59E-28	1.10E-26
UBAP2	9p13.3	-0.326401231	2.81E-28	1.19E-26
KIF20A	5q31.2	-0.326390833	2.82E-28	1.19E-26
AURKA	20q13.2	-0.326123907	3.14E-28	1.32E-26
CDK1	10q21.2	-0.325579255	3.90E-28	1.63E-26
E2F3	6p22.3	-0.325550129	3.94E-28	1.64E-26
MCM4	8q11.21	-0.32498367	4.94E-28	2.02E-26
AHCTF1	1q44	-0.32493747	5.03E-28	2.05E-26
HSP90AB1	6p21.1	-0.324870313	5.16E-28	2.10E-26
FANCB	Xp22.2	-0.32479966	5.31E-28	2.16E-26
NCAPD2	12p13.31	-0.324021906	7.21E-28	2.91E-26
HAUS6	9p22.1	-0.323688469	8.23E-28	3.30E-26
KIF4B	5q33.2	-0.323654292	8.34E-28	3.34E-26
ZNF367	9q22.32|9q22.32	-0.323290238	9.62E-28	3.82E-26
TOMM70	3q12.2	-0.32316816	1.01E-27	4.00E-26
EPCAM	2p21	-0.322935525	1.11E-27	4.35E-26
NUF2	1q23.3	-0.322748214	1.19E-27	4.67E-26
OTUD6B	8q21.3	-0.322724159	1.20E-27	4.71E-26
DHX9	1q25.3	-0.32216442	1.50E-27	5.84E-26
PBK	8p21.1	-0.321993446	1.60E-27	6.23E-26
MSH6	2p16.3	-0.321497635	1.94E-27	7.51E-26
ZFR	5p13.3	-0.321237726	2.15E-27	8.25E-26

KIAA1549	7q34	-0.320471992	2.89E-27	1.09E-25
FAM217B	20q13.33	-0.319829232	3.72E-27	1.38E-25
ORC6	16q11.2	-0.319803057	3.75E-27	1.40E-25
YWHAQ	2p25.1	-0.319658448	3.97E-27	1.47E-25
ZNF711	Xq21.1	-0.319598046	4.06E-27	1.50E-25
SOS1	2p22.1	-0.319500803	4.22E-27	1.56E-25
DOCK7	1p31.3	-0.319002599	5.12E-27	1.87E-25
TRRAP	7q22.1	-0.318974027	5.17E-27	1.89E-25
CEP55	10q23.33	-0.318853305	5.42E-27	1.98E-25
TAF2	8q24.12	-0.31843684	6.37E-27	2.31E-25
MND1	4q31.3	-0.318197949	6.98E-27	2.51E-25
ERCC6L	Xq13.1	-0.318149247	7.11E-27	2.55E-25
SMG7	1q25.3	-0.318034397	7.44E-27	2.66E-25
DLGAP5	14q22.3	-0.317889693	7.86E-27	2.78E-25
PRPF40A	2q23.3	-0.317879529	7.89E-27	2.79E-25
KLHL23	2q31.1	-0.317867089	7.93E-27	2.80E-25
FMR1	Xq27.3	-0.317806208	8.12E-27	2.86E-25
CENPJ	13q12.12-q12.13	-0.31742929	9.39E-27	3.28E-25
SIKE1	1p13.2	-0.317338724	9.72E-27	3.39E-25
PTPN11	12q24.13	-0.317272161	9.97E-27	3.48E-25
CCNB2	15q22.2	-0.317158243	1.04E-26	3.62E-25
OPA1	3q29	-0.316936869	1.13E-26	3.93E-25
NFXL1	4p12	-0.316904823	1.15E-26	3.97E-25
CENPL	1q25.1	-0.316872946	1.16E-26	4.01E-25
UPF2	10p14	-0.316517774	1.33E-26	4.58E-25
SENP1	12q13.11	-0.316442041	1.37E-26	4.71E-25
SENP5	3q29	-0.316051381	1.59E-26	5.41E-25
ZBTB10	8q21.13	-0.316043301	1.60E-26	5.42E-25
ZNF567	19q13.12	-0.315333537	2.09E-26	7.01E-25
PUM2	2p24.1	-0.314471825	2.91E-26	9.57E-25
NUDT21	16q13	-0.314368887	3.02E-26	9.90E-25
HMMR	5q34	-0.314328208	3.07E-26	1.00E-24
SMIM13	6p24.2	-0.313993168	3.49E-26	1.13E-24
MASTL	10p12.1	-0.313870393	3.65E-26	1.18E-24
MCM3AP-AS1	21q22.3	-0.313792637	3.76E-26	1.21E-24
MARS2	2q33.1	-0.31372047	3.86E-26	1.24E-24
NCOA6	20q11.22	-0.313347826	4.45E-26	1.43E-24
USP1	1p31.3	-0.313066579	4.95E-26	1.58E-24
SMC1A	Xp11.22	-0.312848088	5.38E-26	1.71E-24
MYO19	17q12	-0.312734008	5.61E-26	1.78E-24
BORA	13q21.33	-0.312689163	5.71E-26	1.81E-24
GTPBP4	10p15.3	-0.312542538	6.03E-26	1.90E-24
PROSER1	13q13.3	-0.312178641	6.92E-26	2.17E-24
KIFC1	6p21.32	-0.31217483	6.93E-26	2.17E-24
CENPU	4q35.1	-0.311576774	8.68E-26	2.69E-24
RTKN2	10q21.2	-0.311567745	8.71E-26	2.69E-24
NDC80	18p11.32	-0.311326519	9.53E-26	2.94E-24
SETD5	3p25.3	-0.311120873	1.03E-25	3.16E-24
INCENP	11q12.3	-0.310121878	1.50E-25	4.56E-24
PIGW	17q12	-0.31009217	1.51E-25	4.60E-24
SNRNP200	2q11.2	-0.30959643	1.82E-25	5.47E-24
DNMT3B	20q11.21	-0.309273869	2.05E-25	6.13E-24
TROAP	12q13.12	-0.309102621	2.19E-25	6.53E-24
ZNF525	19q13.42	-0.308939784	2.33E-25	6.92E-24
URB2	1q42.13	-0.308886456	2.37E-25	7.04E-24
MCM8	20p12.3	-0.30888177	2.38E-25	7.05E-24
TMEM170A	16q23.1	-0.308641027	2.60E-25	7.67E-24
GRHL2	8q22.3	-0.308584685	2.66E-25	7.81E-24
POM121	7q11.23	-0.308552737	2.69E-25	7.89E-24
ATAD2B	2p24.1-p23.3	-0.307964291	3.34E-25	9.72E-24
SRPK1	6p21.31	-0.307958155	3.35E-25	9.73E-24
PDS5A	4p14	-0.307926836	3.39E-25	9.82E-24
PTK2	8q24.3	-0.30790951	3.41E-25	9.86E-24
STAM	10p12.33	-0.307903272	3.42E-25	9.87E-24
ZNF765	19q13.42	-0.307833241	3.51E-25	1.01E-23
ZNF121	19p13.2	-0.307811254	3.54E-25	1.02E-23
ZNF146	19q13.12	-0.307664872	3.73E-25	1.07E-23
PPM1D	17q23.2	-0.307484047	3.99E-25	1.14E-23

HASPIN	17p13.2	-0.30742769	4.08E-25	1.17E-23
TAF4	20q13.33	-0.307400975	4.12E-25	1.18E-23
MZT1	13q21.33	-0.307299877	4.27E-25	1.21E-23
TRIM37	17q22	-0.307026862	4.73E-25	1.33E-23
HELZ	17q24.2	-0.30700807	4.76E-25	1.34E-23
WDR12	2q33.2	-0.30685036	5.05E-25	1.42E-23
THAP9	4q21.22	-0.306662008	5.41E-25	1.52E-23
GXYLT1	12q12	-0.306541289	5.66E-25	1.58E-23
TRIM33	1p13.2	-0.306405076	5.95E-25	1.66E-23
USP13	3q26.33	-0.306345975	6.08E-25	1.69E-23
LRRC8B	1p22.2	-0.306320829	6.13E-25	1.71E-23
CDC6	17q21.2	-0.306041544	6.80E-25	1.88E-23
PTCD3	2p11.2	-0.305858226	7.27E-25	2.00E-23
ANKRD17	4q13.3	-0.305794945	7.44E-25	2.04E-23
CASP8AP2	6q15	-0.305717992	7.66E-25	2.10E-23
POMK	8p11.21	-0.30557271	8.08E-25	2.21E-23
ANKRD27	19q13.11	-0.304765199	1.09E-24	2.94E-23
MDC1	6p21.33	-0.304748618	1.09E-24	2.96E-23
SPDL1	5q35.1	-0.304729121	1.10E-24	2.97E-23
BIRC5	17q25.3	-0.304474446	1.21E-24	3.26E-23
PRIM2	6p11.2	-0.304404483	1.24E-24	3.33E-23
CDC5L	6p21.1	-0.304381645	1.25E-24	3.36E-23
SINHCAF	12p11.21	-0.304336013	1.27E-24	3.41E-23
ADNP	20q13.13	-0.304236866	1.32E-24	3.53E-23
PPP4R3B	2p16.1	-0.304219384	1.33E-24	3.54E-23
PRKCI	3q26.2	-0.30419718	1.34E-24	3.57E-23
FBXO5	6q25.2	-0.30415706	1.36E-24	3.62E-23
ANKS1A	6p21.31	-0.30413793	1.37E-24	3.64E-23
SUV39H2	10p13	-0.304045312	1.41E-24	3.76E-23
CENPA	2p23.3	-0.303825082	1.53E-24	4.07E-23
TASOR2	10p15.1	-0.3036765	1.62E-24	4.29E-23
MIR924HG	18q12.2-q12.3	-0.303346866	1.82E-24	4.80E-23
QSER1	11p13	-0.303022915	2.05E-24	5.38E-23
ORC1	1p32.3	-0.302898066	2.15E-24	5.62E-23
ITPR3	6p21.31	-0.302856257	2.18E-24	5.70E-23
ZDHHC23	3q13.31	-0.302667051	2.33E-24	6.09E-23
SMC2	9q31.1	-0.302584094	2.40E-24	6.26E-23
CENPQ	6p12.3	-0.30221677	2.75E-24	7.11E-23
MRS2	6p22.3	-0.302087605	2.88E-24	7.43E-23
FOXK2	17q25.3	-0.30185138	3.14E-24	8.06E-23
C18ORF25	18q21.1	-0.301815451	3.18E-24	8.16E-23
XPOT	12q14.2	-0.301756113	3.25E-24	8.32E-23
RBL1	20q11.23	-0.30175329	3.25E-24	8.32E-23
C17ORF80	17q25.1	-0.301215926	3.95E-24	1.01E-22
FANCA	16q24.3	-0.301129956	4.07E-24	1.04E-22
DNM1L	12p11.21	-0.301075129	4.15E-24	1.06E-22
FANCD2	3p25.3	-0.300735647	4.69E-24	1.19E-22
CDC25C	5q31.2	-0.300690102	4.77E-24	1.21E-22
DSC2	18q12.1	-0.300689274	4.77E-24	1.21E-22
RBM12B	8q22.1	-0.300659671	4.82E-24	1.22E-22
ATP6V0A2	12q24.31	-0.30050167	5.11E-24	1.29E-22
PIMREG	17p13.2	-0.300471725	5.16E-24	1.30E-22
NUP160	11p11.2	-0.300315077	5.46E-24	1.37E-22
